# β-Trioxopyrrocorphins: pyrrocorphins of graded aromaticity†

**DOI:** 10.1039/d1sc03403k

**Published:** 2021-08-20

**Authors:** Nivedita Chaudhri, Matthew J. Guberman-Pfeffer, Ruoshi Li, Matthias Zeller, Christian Brückner

**Affiliations:** Department of Chemistry, University of Connecticut Storrs CT 06269-3060 USA nivedita.chaudhri@uconn.edu c.bruckner@uconn.edu; Department of Chemistry, Purdue University 560 Oval Drive West Lafayette IN 47907-2084 USA

## Abstract

Octaethyltrioxopyrrocorphins unexpectedly show macrocycle-aromatic properties, even though they contain the macrocyclic π-system of the non-aromatic pyrrocorphins (hexahydroporphyrins). Two of the four possible triketone regioisomers were first reported in 1969 by one-pot oxidation of octaethylporphyrin but remained essentially unexplored since. We detail here the targeted preparation of the remaining two triketone isomers and the optical and NMR spectroscopic properties of all isomers. All four regioisomers possess unique electronic properties, including broadly varying degrees of diatropicity that were experimentally determined using ^1^H NMR spectroscopy and computationally verified. Structural patterns modulating the aromaticity were recognized. These differences highlight the regioisomerically differentiated influences of the three β-oxo-functionalities. We also present the solid state structure of the two most common isomers (in their free base form or as zinc complexes), allowing further conclusions to be made about the resonance structures present in these triketones. Remarkably, also, the halochromic properties of the triketones differ sharply from those of regular (hydro)porphyrins, providing further support for the proposed 16-membered, 18 π-electron aromatic ring-current. The work conceptually expands the understanding of tris-modified hydroporphyrinoid analogues and the factors that enable and control porphyrinoid aromaticity.

## Abstract 2

The four possible pyrrocorphin isomers were prepared. Remarkably, they show regiochemically dependent aromaticity attributed to the presence of a 16-membered, 18 π-electron aromatic ring-current.

## Graphical abstract

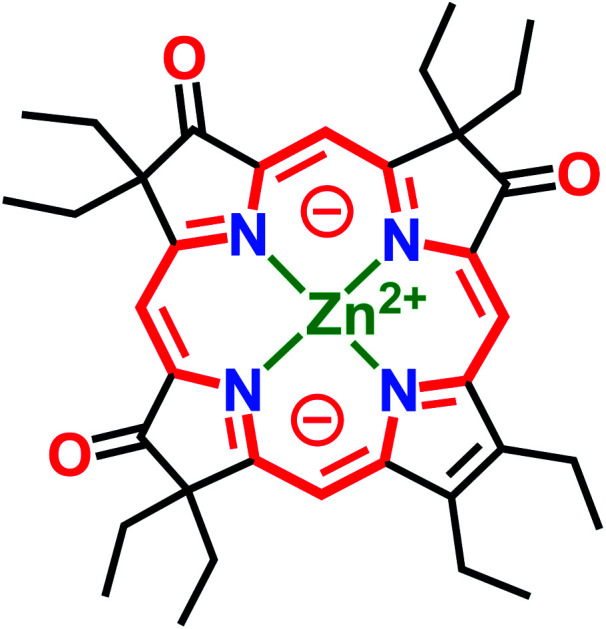

## Introduction

**Fig. 1:**
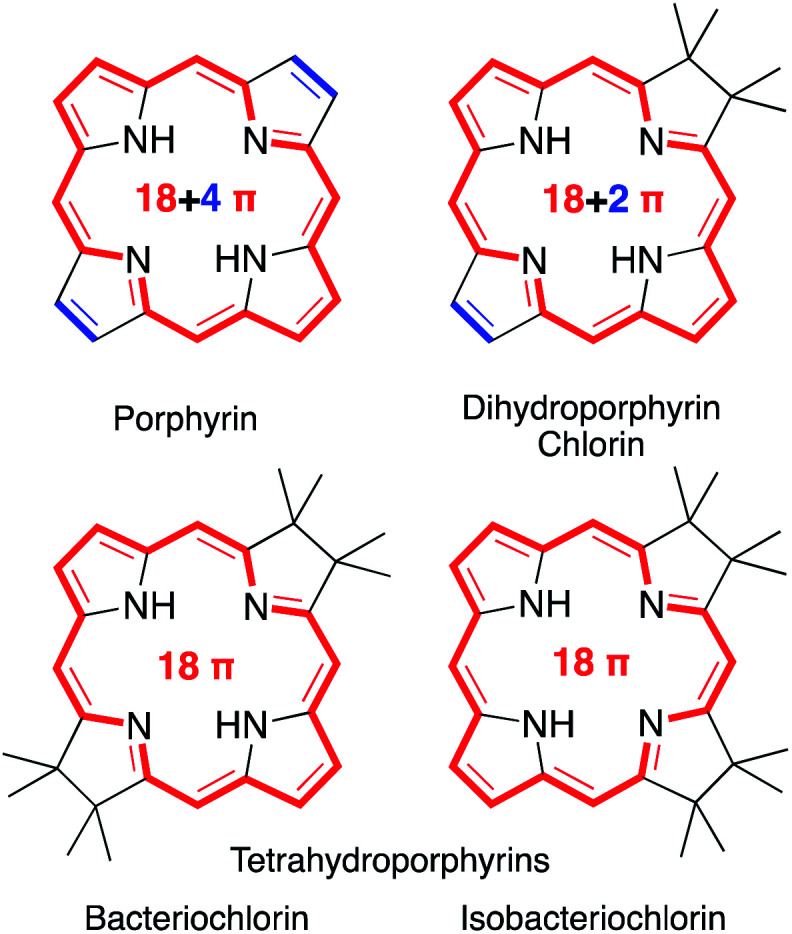
Framework structures of the hydroporphyrin classes indicated. Aromatic π-systems highlighted in red, cross-conjugated double bonds in blue.

Porphyrins are characterized by the presence of a Hückel-aromatic 18 π electron system, cross-conjugated with two additional double bonds, resulting in what is typically described as the porphyrinic 18 + 4 π-system (Fig. 1).

Reactions that convert one or both cross-conjugated β,β′-double bonds to single bonds generate chlorins, bacteriochlorins or isobacteriochlorins, respectively.^1^ The aromatic 18 + 4, 18 + 2, or 18 π-electron systems of the (hydro)porphyrins are primarily responsible for their characteristic electronic properties.^2^ Their resonance structure preferably takes the so-called ‘inner–outer–inner–outer’ pathways indicated.^3^

**Scheme 1:**
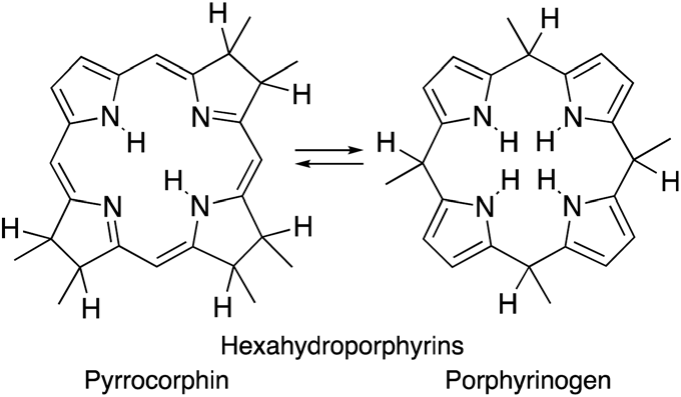
Pyrrocorphin–porphyrinogen tautomeric equilibrium.

Importantly, removal of a third cross-conjugated double bond from the tetrahydroporphyrins results in the interruption of the classic Hückel-aromatic 18 π electron system. Thus, reduction of (hydro)porphyrins may lead to the formation of their leuco-form, the porphyrinogens.^4^ While their free bases are preferentially in the porphyrinogen tautomeric form (5,10,15,20,22,24-hexahydroporphyrin), their nickel(ii) complexes are in the pyrrocorphin form (2,3,7,8,12,13-hexahydroporphyrin) (Scheme 1).^4*a*^

Eschenmoser and co-workers pioneered the studies of synthetic β-alkylpyrrocorphins, such as compounds **1Mg**, in the context of their vitamin B_12_ synthesis.^4*a*,5^ Later, pyrrocorphins were included in studies by the groups of Battersby, Smith, and Stolzenberg.^4*b*,6^ Examples of pyrrocorphins (or pyrrocorphin-like analogues)^7^ of the *meso*-pentafluorophenylporphyrin series emerged more recently:^4*b*,7,8^ For instance, triple-cycloaddition formed pyrrocorphins **2**/**2Ni**;^8*b*^ the reduction of a bacteriochlorin-type bis-adduct realized pyrrocorphin **3**.^8*c*^ Often these pyrrocorphins formed as fortuitous products next to the target compounds. Notably, the electronic properties of the pyrrocorphins did not receive closer scrutiny.
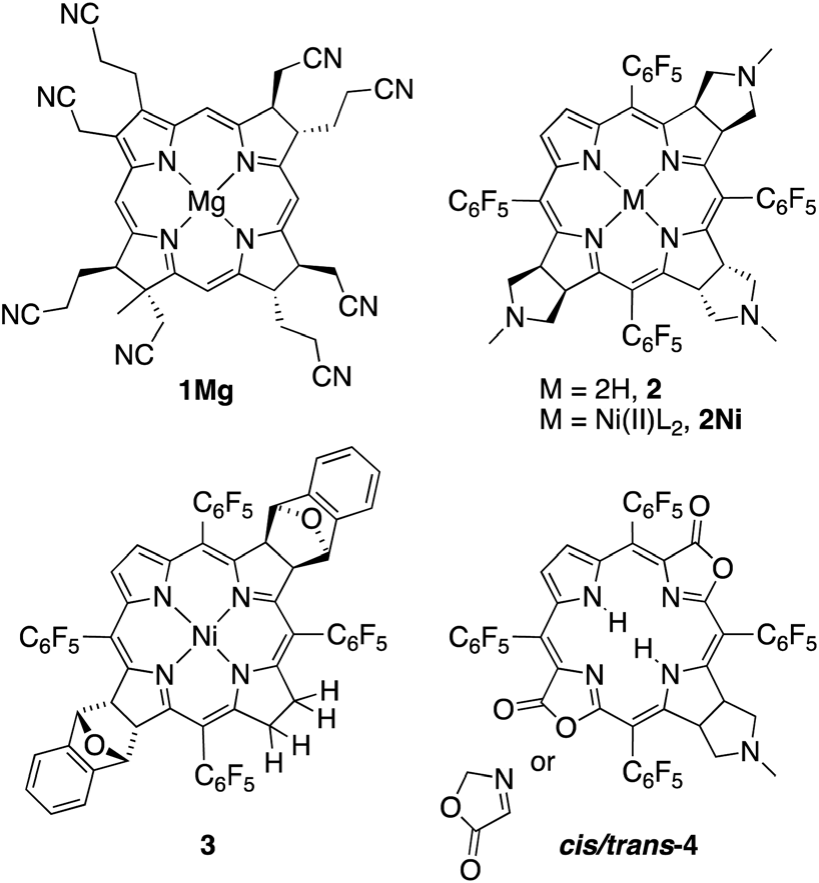


Better studied are the porpholactone-based *meso*-arylporphyrin derivatives prepared by Zhang and co-workers that contain two pyrrolines and one oxazolone or one pyrroline and two oxazolones (such as **cis/trans-4**); they exhibited intermediate aromaticity and strong regioisomeric influences with respect to the two possible relative orientations of the lactone moieties.^7,9^

**Scheme 2:**
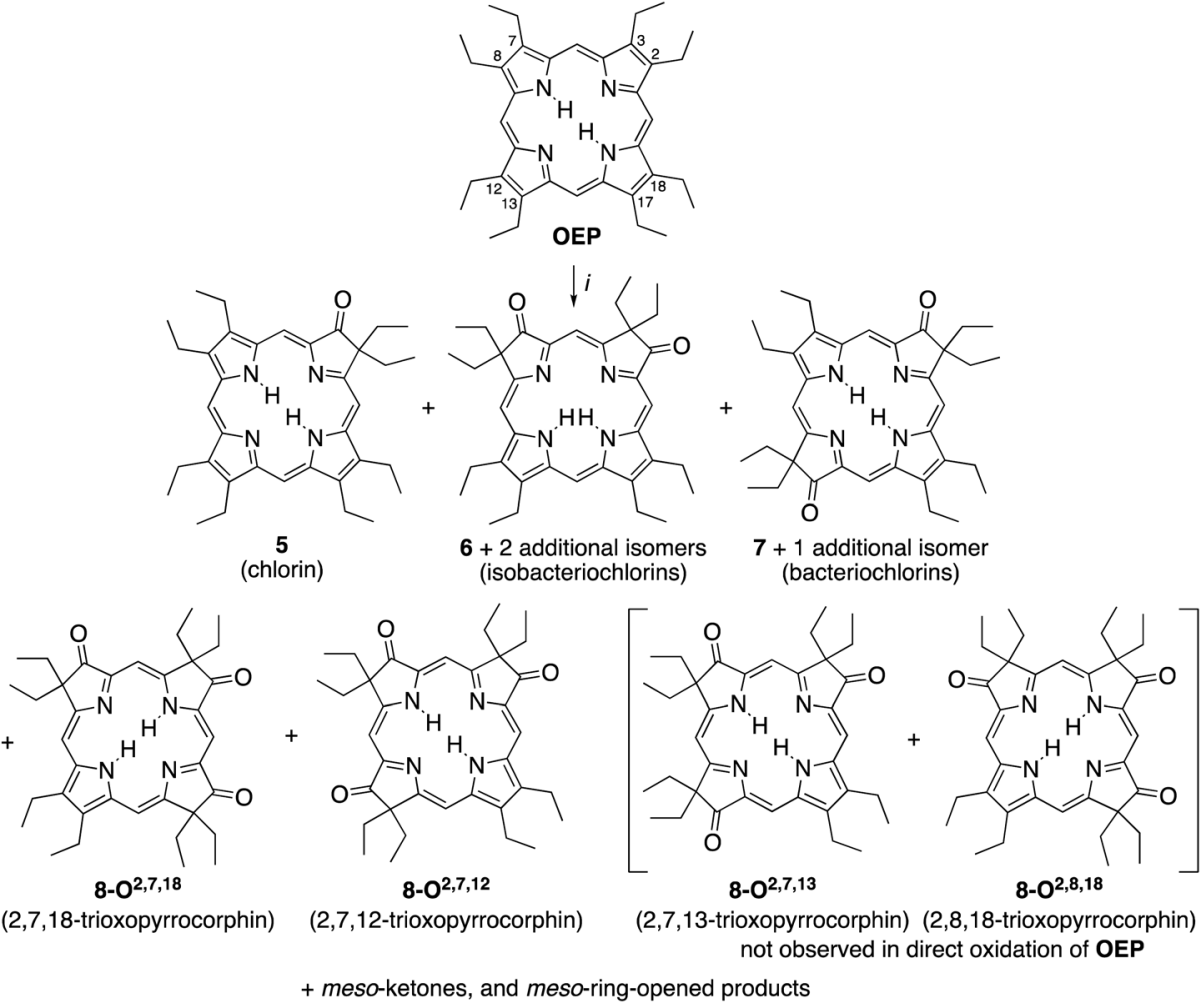
Oxidation of octaethylporphyrin **OEP** to the corresponding β-oxoderivatives.^10*a*,15^ Reaction conditions: (i) (1) H_2_O_2_ (3%)/H_2_SO_4_ (96%), ∼15 min, 0 °C. (2) NaOAc, H_2_O. (3) Chromatography. The atom numbering in octaethylporphyrin **OEP** shows the numbering system used, the basis for the β-ketone names.

A simple method to introduce β-oxo functionalities into β-alkylporphyrins is their treatment with H_2_O_2_ in conc. H_2_SO_4_ (Scheme 2). The major product resulting from the treatment of octaethylporphyrin (**OEP**) with H_2_O_2_/H_2_SO_4_ is oxochlorin **5**.^10^ The chromatographic separation and identification of all products from this reaction – three isomers of the dioxoisobacteriochlorin series **6**, two isomers of the dioxobacteriochlorin series **7**, two (out of the four possible) triketone isomers **8**, as well as *meso*-oxo-substituted phlorins and ring-opened products – were described not long after, in 1969.^11^

While the chemical, coordination, and physical properties of oxochlorin **5**^11*b*,12^ and the dioxo-derivatives **6** and **7 **^12*h,p*,13^ were subject of several studies, the triketones **8** were largely ignored. Nonetheless, their nickel and copper complexes were prepared and their structure, axial binding properties, and electrochemical properties studied.^13*a*,*b*,14^ However, their electronic nature was not further elucidated.

We present here the targeted syntheses of the two ‘missing’ triketone isomers and the description of the unique electronic structure of all four triketones isomers, as probed by UV-vis, fluorescence, and NMR spectroscopy, as well as DFT computations. The solid-state structures of two members of the triketones confirm their connectivity and conformational assignment. They also support the formulation of a 16-membered, 18 π-electron aromatic system present in these compounds. We also describe the halochromic properties of all four triketones, again setting this compound class much apart from regular (hydro)porphyrins or pyrrocorphins.

The work provides new concepts of how three β-oxo functionalities modulate the chemical and electronic properties of porphyrinoids to the extent that the usually nonaromatic π-systems of pyrrocorphins assume the spectroscopic and structural characteristics of an aromatic macrocycle, whereby stark regiochemical differences are noted.

## Results and discussion

### Formation of the known and novel β-trioxopyrrocorphin isomers

The syntheses of the two previously known trioxopyrrocorphins **8-O2,7,12** and **8-O2,7,18** by oxidation of **OEP** proceeded as described by Inhoffen and Nolte, and later Chang (Scheme 2):^11^ Multi-gram batches of **OEP** were reacted in 96% H_2_SO_4_ with 3% H_2_O_2_ at ice temperatures over a period of 15 to 20 min. The reaction was neutralized, and the product cocktail extracted with an organic solvent and chromatographed (using multiple columns; select fractions required further separations by preparative plate chromatography). This allowed us to isolate, among all the other products formed, the two isomers of trioxopyrrocorphin title compounds **8-O2,7,18** (in 2% yield) and **8-O2,7,12** (in 5.5% yield).‡‡All experimental procedures and spectroscopic data are presented in the ESI.^11^

It is reasonable to assume that **OEP** was converted in three distinct and sequential pyrrole-to-pyrrolinone steps in this one-pot reaction, with each step constituting a series of reactions (epoxidation, epoxide ring opening, followed by a pinacol–pinacolone rearrangement). Some regioselectivity in the sequential reactions is expressed as only two of the four theoretically possible isomers of the trioxopyrrocorphins **8** were reported to form.^10*b*,11*b*,15^ In fact, our own careful screening (by ESI + MS) of the many minor products formed did not reveal any indication that the two other possible triketone isomers were among them.

When we oxidized ketone **5** under the same conditions as **OEP**, we found that the absolute yields for the di- and triketones improved. However, we found the relative product profile was otherwise similar to that of the oxidation of **OEP**.

An analysis of which dioxoisomer (**6** or **7**) could theoretically form which triketone upon further oxidation reveals that all dioxoisomers are not equally suited to form the missing triketone isomers (Table 1). We then proceeded to oxidize diketone isomers **6** or **7** individually using harsher reactions conditions (using twice the stoichiometric ratio of H_2_O_2_; for details, see ESI†). The product outcomes relative to the theoretical possible outcomes were tabulated. The diketones that were predicted to be theoretically suitable to form the two known triketones **8-O2,7,18** and **8-O2,7,12** indeed formed them also experimentally. However, some reactions that theoretically could have formed the hitherto unknown triketones **8-O2,7,13** and **8-O2,8,18** failed to produce them. More importantly, however, some of the reactions theoretically capable of forming the latter actually generated them. The novel products were identified based on their compositions (as per ESI + HR-MS), NMR and UV-vis spectroscopic properties (see below).‡ They are also, like the previously known congeners, chemically robust and no special conditions were needed for their chromatographic isolation and purification, recrystallization, handling, or storage in solid form.

**Table 1:** Theoretical and practical outcomes of the oxidation of a given dioxochlorin with respect to the formation of a specific trioxopyrrocorphin isomer. The coloured arrows on the diketone starting materials indicate the position of the oxo-groups that leads to the formation of the triketone isomer of the same colour. The black crosses indicate the reactions could not theoretically form the trioxopyrrocorphins indicated. The checkmarks indicate successful reactions; the parentheses indicate the experimentally observed % isolated yield. The red crosses indicate the reactions that could have led to the formation of the trioxopyrrocorphins indicated, but did not. For the reaction conditions, see ESI

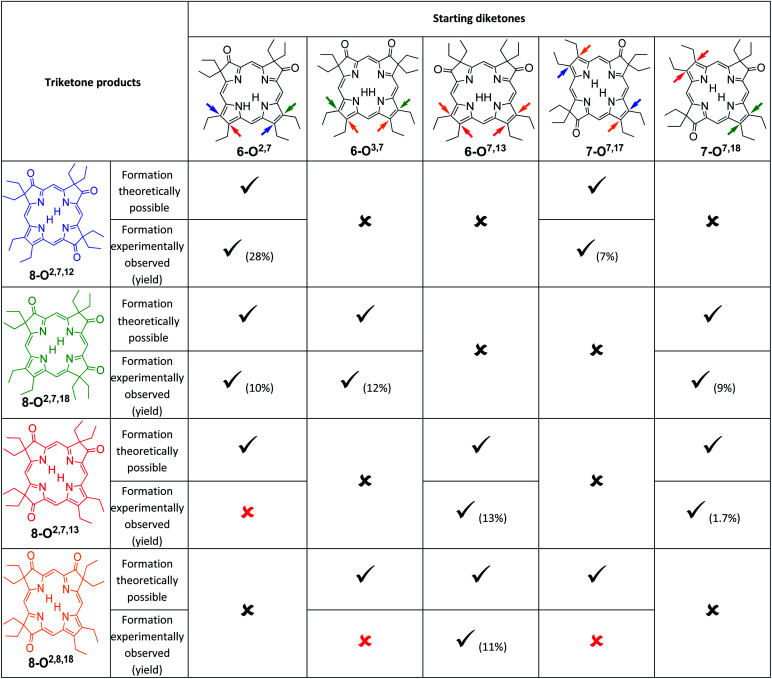

We note that the most successful reactions start from the dioxoisobacteriochlorin isomer **6-O7,13** containing already a set of *gem*-diethyl groups facing each other. Unfortunately, this isomer is also the most rare of the diketones, forming in nearly an order of magnitude lower yields than the next least common isomer.^11^ Inversely, the formation of the two triketones containing sets of *gem*-diethyl groups facing each other (**8-O2,7,13** and **8-O2,8,18**) from diketone precursors not already containing this feature failed or were very inefficient. We assume kinetic barriers to play a role during the formation of the products under the harshly acidic conditions as the computed heats of formation of the four triketone isomers provide no indication for the existence of major thermodynamic constraints (such as steric inhibitions) in the isomers containing *gem*-diethyl groups facing each other: With the heat of formation of **8-O2,7,13** set to be 0.0 kJ mol^−1^, that of **8-O2,7,12** is 2.2 kJ mol^−1^ higher, that of **8-O2,8,18** is 3.1 kJ mol^−1^ higher, and the highest, for **8-O2,7,18**, is computed to be only 5.3 kJ mol^−1^ higher.

### NMR spectroscopic properties of the trioxopyrrocorphins

**Fig. 2:**
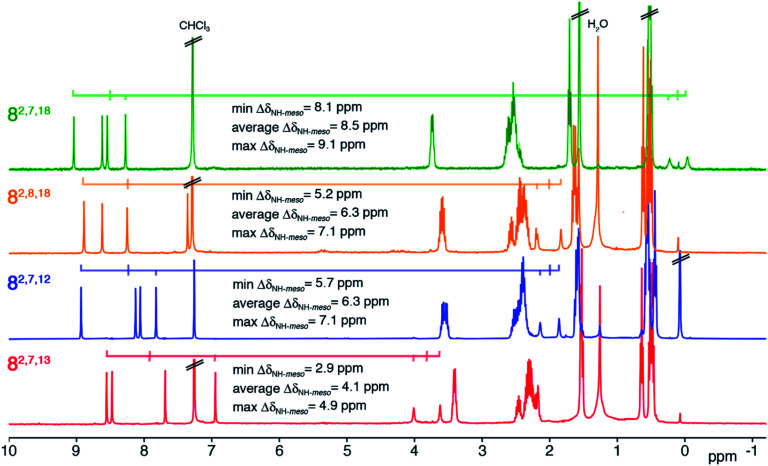
^1^H NMR spectra (400 MHz, CDCl_3_, 25 °C) of the trioxopyrrocorphin isomers indicated. For details, see ESI.†

The ^1^H NMR spectra of all four trioxopyrrocorphin isomers show, next to the multiple signals for the ethyl groups, the presence of four distinct low-field singlets (1H each), assigned to the four *meso*-CH hydrogens, and two broader singlets (1H each) assigned to the inner NH hydrogen atoms (Fig. 2). The *meso*-CH peaks are generally slightly upfield shifted compared to the corresponding signals in oxochlorin **5** or dioxochlorins **6** and **7**, while the NH signals are considerably shifted downfield.^11,16^ The ^13^C spectra of all isomers show three distinct signals for carbonyl carbon atoms (between 204 to 209 ppm). HMBC and HSQC spectra allowed unambiguous assignments of most hydrogen and carbon signals of the chromophores (see ESI†).

The diatropic shifts of the hydrogens inside and outside the aromatic macrocyclic π-system and particularly the spread of shifts between them (Δ*δ*_NH-*meso*_) was taken as one measure for the degree of aromaticity of the macrocyclic π-system.^9,17^ The average Δ*δ*_NH-*meso*_ values found in the four trioxopyrrocorphins vary widely, ranging from 8.5 ppm for isomers **8-O2,7,18** – a value indicative of a system of substantial aromatic character, down to 4.1 ppm for isomer **8-O2,7,13** – a value indicative of a system with much reduced diatropic ring current. The spread for isomers **8-O2,8,18** and **8-O2,7,12** (both 6.3 ppm) falls between the two extremes. The corresponding Δ*δ*_NH-*meso*_ values of 13.8 ppm for **OEP**, 12.5 ppm for **5,** 12.0, 11.0 ppm for dioxo-bacteriochlorins **7-O7,17** and **7-O7,18** and the average Δ*δ*_NH-*meso*_ values of 9.0, 10.8, and 6.75 ppm for dioxo-isobacteriochlorins **6-O,2,76-O,3,7** and **6-O,7,13** respectively, serve as benchmark values for fully aromatic macrocycles.^11,16^ Computations, structural, and chemical findings for the triketones presented below shed further insight into the prevalent ring currents.

### UV-vis and fluorescence spectra of the trioxopyrrocorphins

**Fig. 3:**
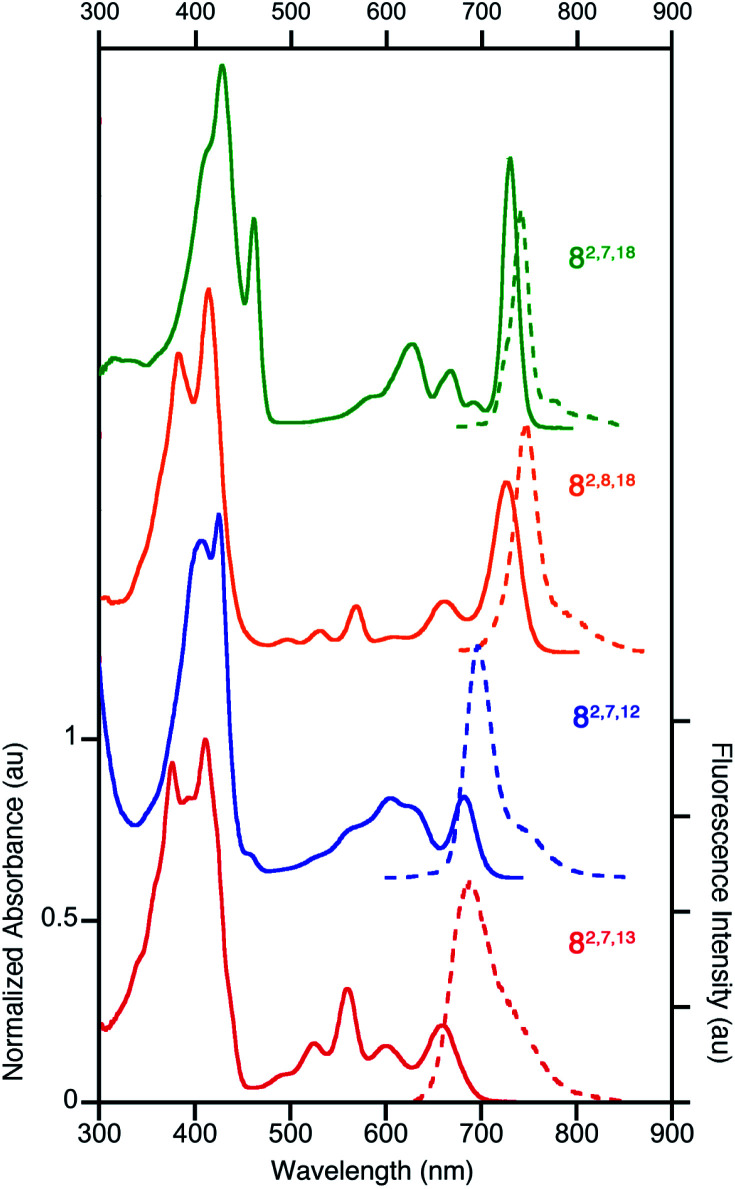
Normalized UV-vis absorption (solid trace) and fluorescence emission (broken trace) spectra of the trioxopyrrocorphins indicated (all in CH_2_Cl_2_); *λ*_excitation_ = *λ*_Soret-max_. For their extinction coefficients, see ESI.†

All four triketone isomers possess distinct UV-vis spectra of aromatic porphyrinoid character (Fig. 3):^11,16^ a (split) Soret band feature in the blue region and well-defined side bands. In some cases, four side bands are clearly distinguishable, as for any typical porphyrin or chlorin. Isomer **8-O2,7,18** possesses a chlorin-type absorption spectrum, the spectrum of **8-O2,8,18** resembles more that of a bacteriochlorin, while those of **8-O2,7,12** and **8-O2,7,13** are dissimilar to any hydroporphyrin archetype. The regioisomeric influence of the auxochromic β-oxo-substituents was noted also for other β-oxo-derivatives,^1*c*,18^ including the porphodilactones.^7,9,19^

The *λ*_max_ bands of the isomers span a range of ∼70 nm (from 659 nm for **8-O2,7,13** to 727 nm for **8-O2,7,18**), whereby the isomers of the longest wavelength *λ*_max_ band position broadly possess the largest Δ*δ*_NH-*meso*_ values (or other aromaticity parameters, see below), and *vice versa*. Such a correlation between *λ*_max_ band position and aromaticity parameters was also suggested in recent work on porpholactones.^9^ However, there is a notable difference in the *λ*_max_ band positions (727 nm and 682 nm for **8-O2,8,18** and **8-O2,7,12**, respectively) of the isomers of intermediate and near-identical aromaticity parameters.

The fluorescence spectra of all triketone isomers are, as typical for hydroporphyrins, primarily single band spectra (with a shoulder) with a small Stoke's shift. The fluorescence emission yields are relatively high and range between 8.5% (for **8-O2,7,18**) and 17.8% (for **8-O2,8,18**) (see ESI† for details).

### Computed aromaticity parameters

**Fig. 4:**
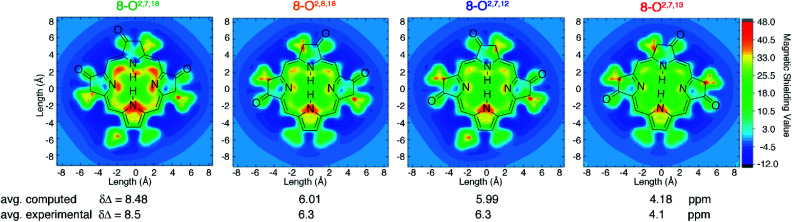
Visualization of differential diatropicity in the triketone isomers: (top) isochemical magnetic shielding heat maps at a height of 1.0 Å above the macrocyclic plane and (bottom) average inner *versus* outer ^1^H NMR chemical shifts (Δ*δ*). All computed quantities were obtained at the BH and HLYP/def2-TZVP level of theory. The iso-chemical shielding surface approach (ICSS)^20^ a three-dimensional generalization of the nucleus independent chemical shift (NICS) technique,^21^ as implemented in MultiWFN^22^ was employed to visualize the spatial distribution of the *ZZ* component of the magnetic shielding tensor 1.0 Å above the macrocyclic plane. Note that the ethyl substituents exhibit significant diatropicity because the viewing plane bisects these groups. For further details, see ESI.†

DFT computations quantitatively reproduce the distinct Δ*δ*_NH-*meso*_ values distributed from 4.1 to 8.5 ppm for the trioxopyrrocorphin isomers to within 0.4 ppm. We therefore extended the analysis of aromaticity to visualize the spatial distribution of the perpendicular component of the magnetic shielding 1.0 Å above the macrocyclic plane (Fig. 4).

The most aromatic isomer, **8-O2,7,18**, is strongly diatropic, particularly in the vicinity of the pyrrolic nitrogen and, to a lesser degree, the imine nitrogen atoms and the *meso*-carbon atoms flanking the protonated pyrrolinone moiety.

Across the isomer series, the intensity of the diatropic character changes much more than its spatial distribution, but patterns in the variations can be recognized. The change from a single *cis*- to *trans*-orientation of an oxo group starting from the most aromatic isomer (**8-O2,7,18** → **8-O2,8,18** or **8-O2,7,12**) causes a 2.2 ppm reduction in Δ*δ*_NH-*meso*_. Inversely, starting from the least aromatic isomer, the same *cis*-to-*trans*-isomerism (**8-O2,7,13** → **8-O2,8,18** or **8-O2,7,12**) causes Δ*δ*_NH-*meso*_ to increase by 2.2 ppm. The consistently sized but oppositely directed shifts in Δ*δ*_NH-*meso*_ are related to the *syn*- or *anti*-dispositions of the oxo groups on the N–N axis relative to the oxo group on the NH–NH axis. Thus, the most (**8-O2,7,18**) and least (**8-O2,7,13**) aromatic isomers have Δ*δ*_NH-*meso*_ values differing by 4.4 ppm, because they have two *syn*- or two *anti*-oxo groups, respectively. This analysis delineates a quantitative structural basis for the grades of aromaticity among the trioxopyrrocorphin isomers that can be used to rationally design other chromophores of the β-oxopyrrocorphin type. The understanding expands the analysis of the aromaticity of bisporpholactone-based chromophores.^7,9^

### Solid state structures of the major trioxopyrrocorphins

**Fig. 5:**
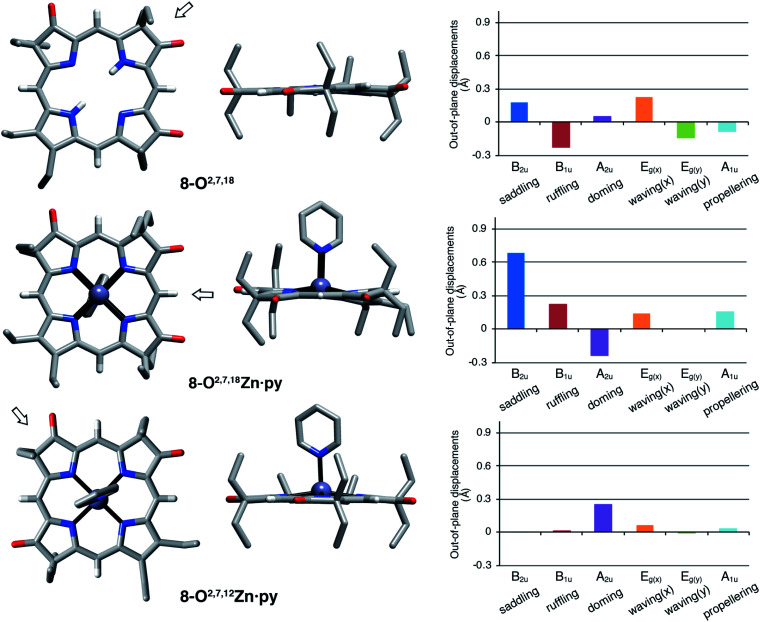
Stick representation of the X-ray single crystal structures of the trioxopyrrocorphin isomers indicated, top and side views; the arrow in the top view images shows the point of view of the corresponding side view. All disorder and solvents (if present) and all ethyl-hydrogen atoms were removed for clarity. NSD analysis as implemented by Kingsbury and Senge.^23^ For details to the structural determinations and structural analyses, see ESI.†

The solid state structures of free base **8-O2,7,18**, its zinc complex **8-O2,7,18Zn·py**, and the isomeric zinc complex **8-O2,7,12Zn·py** (zinc complexes prepared by insertion of zinc(ii) into the corresponding free bases)‡ could be solved (Fig. 5). The free base compound **8-O2,7,18** is characterized by the presence of only minor distortions distributed over all normal distortion modes. The doming and saddling conformational modes are enhanced in its zinc(ii) complex, as frequently observed in porphyrin zinc(ii) complexes with a single axial ligand.^23^ Its nickel(ii) complex was shown to be, as expected when porphyrinoids are coordinated to a relatively small diamagnetic d^8^-ion,^23,24^ non-planar, with significant ruffling modes.^13*b*^

Complex **8-O2,7,12Zn·py** is more planar than the other two compounds, with only a – likely metal-induced – minor doming deformation.^23,25^ The only other 2,7,12-trioxopyrrocorphin structure known is that of the copper π-cation radical [**8-O2,7,12Cu**]^+^, also of only modest distortions. We infer that the **8-O2,7,12** framework is intrinsically more planar than its isomer **8-O2,7,18**.

The general planarity of the macrocycles is an important parameter when assessing the electronic properties of porphyrinoids as deviations from planarity modulate their electronic structure to a significant degree.^26^ Here, because of the idealized planar structure of the compounds, we can assume conformational effects to play only a diminished role and we can attribute the electronic properties to the π-system decorated with β-oxo- and sp^3^-hybridized β-carbon atoms.

**Fig. 6:**
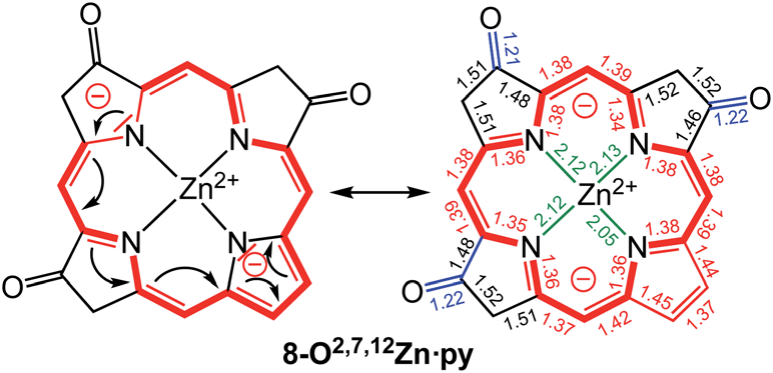
Proposed resonance structure based on the bond length analysis of the solid state structure of **8-O2,7,12Zn·py**. Axial pyridine group and all ethyl substituents omitted for clarity. Coordination bonds: green; isolated double bonds: red; single bonds (>1.44 Å): blue; conjugated double bonds (between 1.35 and 1.45 Å): red. For an equivalent analysis for the other structures, see ESI.† In comparison, bond lengths in [octaethylporphyrinato]Zn(ii) (CCDC code ALOKOB): coordination bonds: 2.04 Å; C_α_–N bonds: 1.36 Å; conjugated C=C double bonds: between 1.37 and 1.45 Å.

A macrocycle bond distance analysis of the solid state structure of **8-O2,7,12Zn·py** provides structural indications for the contribution of a surprising ‘inner–inner–inner–inner’ resonance structure that involves 16 heavy atoms and 18 π-electrons (Fig. 6) (an equivalent analysis can be performed for the zinc complex of the other isomer and the free base chromophore (see ESI† for details).

Computations provided further support for the existence of this conjugation pathway: based on the minimized triketones, we computed hypothetical structures in which the 16 members of the ‘inner–inner–inner–inner’ resonance pathway were maintained but all other carbon atoms were replaced by hydrogen atoms (for details, see ESI†). While such macrocycles returned reduced diatropic ring currents, they still carried a diatropic ring current that displayed the same regioisomer-dependent trends in their strengths as the parent compounds.

The group of Fowler previously showed, using an ipsocentric orbital-based model, that the 16-membered 18 π-electron ‘inner–inner–inner–inner’ conjugation path of the macrocycles proposed here also described the orbital π-current density maps of porphyrin dianions (in the absence of a metal or protons).^27^

### Halochromic properties of the trioxopyrrocorphins

Free base porphyrins are, by virtue of the presence of two inner imine nitrogen atoms, Brønsted-basic. But as free base porphyrins also contain two pyrrole-like NH protons, they can also act as Brønsted acids. However, deprotonation of free base porphyrins in the absence of a coordination metal requires very strong bases (such as alkaline/alkaline earth hydrides or lithium organyls).

**Fig. 7:**
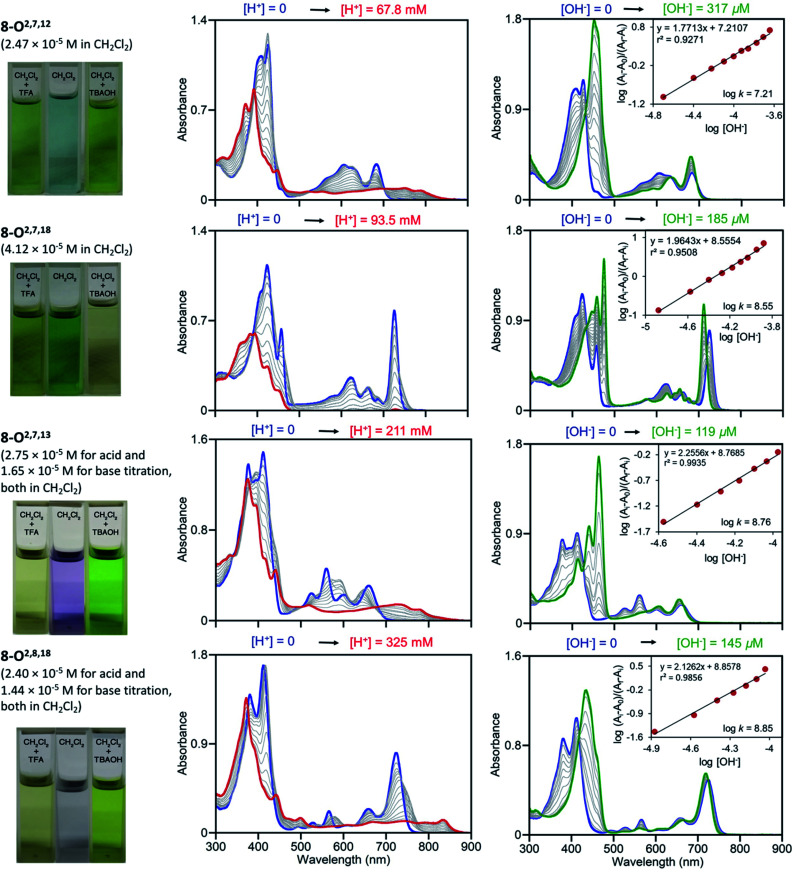
UV-vis titration of triketone isomers **8** indicated; acid titration using TFA (left column) and base titration using TBAOH (right column) in the [H^+^]/[OH^−^] ranges indicated. The photographic images on the left show the colours of the solutions at their start and end points, see ESI.†

We performed UV-vis titrations of all triketone isomers **8** with trifluoroacetic acid (TFA) and tetrabutylammonium hydroxide (TBAOH) in CH_2_Cl_2_ (Fig. 7). Surprisingly, all isomers show strong responses to acid and base, and all of them show quantitatively and qualitative different responses.

In light of the foregoing, the response to acid does not surprise *per se*, but the nature of the response is untypical for porphyrins. Upon protonation, the Soret bands of all triketone isomers blue-shift and lose intensity. The side-bands exhibit significant red-shifts, also at lower intensities; in case of isomer **8-O2,7,18** the side bands completely lose definition. Clear isosbestic points cannot be made out in any of the cases and the corresponding Hill plots are convoluted (for details, see ESI†). This indicates a complex protonation behavior, likely involving multiple binding sites (inner nitrogen atoms and oxo functionalities).^13*f*^ Accordingly, there is little match between the modeled spectra of the bis-imine nitrogen protonated species and the experimental spectra (see ESI†). Also, p*K*_b_ values or protonation stoichiometries could not be determined but relative basicities could be estimated as the [TFA] concentrations at the half end point value of the titration ([TFA]/2 value) (for details, see ESI†). All four pyrrocorphins require much higher TFA concentrations for half protonation (**8-O2,7,12**: [TFA]/2 = 3.80 × 10^−2^ M; **8-O2,7,18**: [TFA]/2 = 4.69 × 10^−2^ M; **8-O2,7,13**: [TFA]/2 = 7.66 × 10^−2^ M; **8-O2,8,18**:[TFA]/2 = 1.01 × 10^−1^ M). These values are much higher than needed for the half-protonation of monooxochlorin **5** ([TFA]/2 = 1.02 × 10^−4^ M), and comparable or slightly higher compared to the least basic dioxochlorins **7-O7,17** ([TFA]/2 = 4.27 × 10^−2^ M).^13*f*^ This characterizes the trioxopyrrocorphins as very weakly basic porphyrinic macrocycles. The low basicity of the inner nitrogen atoms in these triketones also explains why ketone oxygen protonation becomes competitive. Indirect evidence for the low basicity of pyrrocorphins was previously provided through axial ligand binding studies of the nickel(ii) complex **3Ni**.^8*a*^

Neither regular porphyrins nor the monooxo- and dioxoderivatives respond to TBAOH under the conditions applied here (see ESI†). All triketones however, show a strong halochromic response upon addition of base (for details, see ESI†): the Soret bands of all isomers intensify and exhibit significant red-shifts; the well-defined Q-bands retain their definition, but slightly blue-shift and intensify. The titration curves possess clear isosbestic points and the corresponding linear Hill plots suggest a single step, seemingly two-proton deprotonation; equilibrium constants could be derived (**8-O2,7,12**: *K* = 1.62 × 10^−7^ M^−1^; **8-O2,7,18**: *K* = 3.55 × 10^−8^ M^−1^; **8-O2,7,13**: *K* = 5.75 × 10^−8^ M^−1^; **8-O2,8,18**: *K* = 7.08 × 10^−8^ M^−1^). The relative facile deprotonation of both inner NH groups highlights a large increase of acidity of these compounds upon the introduction of three β-oxo-functionalities to the macrocycle. However, a simultaneous two-proton deprotonation of these dibasic chromophores is, in the absence of, for instance, a large conformational change – not deemed to be taking place here – unlikely. We could model the spectra of the mono- (either at the pyrrole or pyrrolinone NH) or bis-deprotonated species (see ESI†), but neither showed a good match with all experimental spectra under basic conditions. On the other hand, ^1^H NMR titrations for the two triketone isomers **8-O2,7,12** and **8-O2,7,18** with TBAOH clearly showed the gradual disappearance of both NH protons, with no other processes becoming evident (for details see ESI†).

In light of the formation of an 18 π-aromatic aromatic and thusly greatly stabilized dianionic conjugated base, the unusually high acidity of the NH protons in the triketones is readily rationalized.

Thus, the behaviour of the triketones in the presence of base is also much different compared to that of the base-sensing porpholactones, *meso*-aryl derivatives carrying a lactone functionality at their β,β′-position.^28^ In porpholactones, the β-carbonyl functionality is susceptible to nucleophilic attack by a range of nucleophiles, including OH^−^, leading to a strong halochromic response.^29^ More investigations to authenticate the nature of (de)protonation events in the triketones and, in general, the origin of their aromaticity are ongoing.

## Conclusions

In conclusion, all four possible β-trioxopyrrocorphins could be prepared. In stark contrast to regular pyrrocorphins, they exhibit macrocycle aromatic character, as seen by the diatropic shifts of the framework protons in their ^1^H NMR spectra and their computed aromaticity parameters. All isomers are believed to be essentially planar (this was confirmed for two isomers). Thus, their electronic properties could be solely attributed to the presence of the β-oxo-substituent, a known class of potent auxochromes.^1,18^ The α-*gem*-dialkylated β-oxo-substituents facilitate the expression of an ‘inner–inner–inner–inner’ resonance pathway involving 18 π-electron distributed over 16 heavy atoms. The X-ray crystal structures provided structural evidence for this conjugation pathway. The acid–base properties of trioxopyrrocorphins are also unusual as they are more weakly basic and much more strongly acidic than regular (hydro)porphyrins, or their mono- and di-β-oxo-analogues. As a result, the triketones show strong halochromic responses upon addition of acid or base. The high acidity could also be linked to the unusual aromatic ring current system of the conjugate base.

This works lays the foundation for the further study of these readily accessible β-trioxopyrrocorphins and contributes insights into the electronic structure of β-oxoporphyrinoids. The finding that the trioxopyrrocorphins enable a 16-membered, 18 π-electron aromatic ring current where none could have been expected to be present is a novel concept. The findings expand the toolset for the precise modulation of porphyrinoid optical properties and aromaticity by systematically varying the number and relative placement of three β-oxosubstituents around a (hydro)porphyrinoid framework. The trioxopyrrocorphins possess the potential to be used as novel chemosensing chromophores.

## Data availability

Crystallographic data for **8-O2,7,18Zn·pyridine**, **8-O2,7,18**, and **8-O2,7,18Zn·pyridine** have been deposited at the CCDC under CCDC 2064957–2064959.

## Author contributions

NC: lead of the investigation, data curation and analysis, wrote the original draft, review & editing of final manuscript, data visualization. RL: early investigations. MJG: performed all computations and contributed to data analysis and visualization. MZ performed all single crystal diffractometry analyses. CB: conceptualization, data analysis and visualization, funding acquisition, project administration, writing, review & editing of final manuscript with input from all authors. C-KC: provision of study materials.

## Conflicts of interest

There are no conflicts to declare.

## Supplementary Material

SC-012-D1SC03403K-s001

SC-012-D1SC03403K-s002
