# Supplementary material for: β-Trioxopyrrocorphins: pyrrocorphins of graded aromaticity
Source: Chem Sci. 2021 Aug 20;12(37):12292–301. doi: 10.1039/d1sc03403k (PMC8480330; doi:10.1039/d1sc03403k)
Supplement: SC-012-D1SC03403K-s001 [file SC-012-D1SC03403K-s001.pdf]

Electronic Supplementary Material (ESI) for Chemical Science.
This journal is © The Royal Society of Chemistry 2021

Electronic Supporting Information (ESI) to:

β-Trioxopyrrocorphins: Pyrrocorphins of graded aromaticity

Nivedita Chaudhri,[a] Matthew J. Guberman-Pfeffer[a], Ruoshi Li[a],*

*Matthias Zeller[b], and Christian Brückner[a]**

[a] *Department of Chemistry University of Connecticut, Unit 3060, Storrs, CT 06269-3060, U.S.A.*

[b] *Department of Chemistry Purdue University, 560 Oval Drive, West Lafayette, IN 47907-2084, U.S.A.*

* Corresponding authors: E-mail: Nivedita.chaudhri@uconn.edu (NC) and c.bruckner@uconn.edu (CB)

ESI Table of Contents

Reproduction of Spectra 10

Free Base Trioxopyrrocorphins 10

Figure S1. ^{1}H NMR spectrum (400 MHz, $CDCl_3$ at 25 °C) of **8-O^{2,7,12}**. 10

Figure S2. ^{13}C NMR spectrum (125 MHz, $CDCl_3$ at 25 °C) of **8-O^{2,7,12}** 11

Figure S3. HSQC NMR spectrum ($CDCl_3$, 25 °C) of **8-O^{2,7,12}**. 11

Figure S4. HMBC NMR spectrum ($CDCl_3$, 25 °C) of **8-O^{2,7,12}**. 12

Figure S5. UV-vis (CH_2Cl_2) absorption (solid line) and fluorescence emission (CH_2Cl_2) spectra (broken line) of **8-O^{2,7,12}**. 12

Figure S6. FT-IR Spectrum (neat, ATR) of **8-O^{2,7,12}** 13

Figure S7. HR-MS Spectrum (ESI+, 100% CH_3CN, TOF) of **8-O^{2,7,12}**. 13

Figure S8. ^{1}H NMR spectrum (400 MHz, $CDCl_3$ at 25 °C) of **8-O^{2,7,18}**. 14

Figure S9. ^{13}C NMR spectrum (125 MHz, $CDCl_3$ at 25 °C) of **8-O^{2,7,18}** 14

Figure S10. HSQC NMR spectrum ($CDCl_3$, 25 °C) of **8-O^{2,7,18}**. 15

Figure S11. HMBC NMR spectrum ($CDCl_3$, 25 °C) of **8-O^{2,7,18}**. 15

Figure S12. UV-vis (CH_2Cl_2) absorption (solid line) and fluorescence emission (CH_2Cl_2) spectra (broken line) of **8-O^{2,7,18}**. 16

Figure S13. HR-MS Spectrum (ESI+, 100% CH_3CN, TOF) of **8-O^{2,7,18}**. 16

Figure S14. FT-IR Spectrum (neat, ATR) of **8-O^{2,7,18}** 17

Figure S15. ^{1}H NMR spectrum (400 MHz, $CDCl_3$ at 25 °C) of **8-O^{2,7,13}**. 17

Figure S16. ^{1}H NMR spectrum (400 MHz, DMSO-D_6 at 25 °C) of **8-O^{2,7,13}** before (red color) and after (blue color) D_2O exchange. 18

Figure S17. ^{13}C NMR spectrum (125 MHz, $CDCl_3$ at 25 °C) of **8-O^{2,7,13}** 18

Figure S18. UV-vis (CH_2Cl_2) absorption (solid line) and fluorescence emission (CH_2Cl_2) spectra (broken line) of **8-O^{2,7,13}**. 19

Figure S19. HR-MS Spectrum (ESI+, 100% CH_3CN, TOF) of **8-O^{2,7,13}**. 19

Figure S20. ^{1}H NMR spectrum (400 MHz, $CDCl_3$ at 25 °C) of **8-O^{2,8,18}**. 20

Figure S21. ^{1}H NMR spectrum (400 MHz, DMSO-D_6 at 25 °C) of **8-O^{2,8,18}** before (red color) and after (blue color) D_2O exchange. 20

Figure S22. ^{13}C NMR spectrum (125 MHz, $CDCl_3$ at 25 °C) of **8-O^{2,8,18}** 21

Figure S23. HSQC NMR spectrum ($CDCl_3$, 25 °C) of **8-O^{2,8,18}**. 21

Figure S24. HMBC NMR spectrum ($CDCl_3$, 25 °C) of **8-O^{2,8,18}**. 22

Figure S25. UV-vis (CH_2Cl_2) absorption (solid line) and fluorescence emission (CH_2Cl_2) spectra (broken line) of **8-O^{2,8,18}**. 22

Figure S26. HR-MS Spectrum (ESI+, 100% CH_3CN, TOF) of **8-O^{2,8,18}**. 23

Zn(II)-Complexes of Trioxopyrrocorphins 24

Figure S27. ^{1}H NMR spectrum (400 MHz, DMSO-d$_6$ at 25 °C) of **Zn(II)-8-O^{2,7,12}**. 24

Figure S28. ^{13}C NMR spectrum (101 MHz, DMSO-d$_6$ at 25 °C) of **Zn-8-O^{2,7,12}**. 24

Figure S29. UV-vis (CH_2Cl_2) absorption (solid line) and fluorescence emission (CH_2Cl_2) spectra (broken line) of **Zn-8-O^{2,7,12}**. 25

Figure S30. FT-IR Spectrum (neat, ATR) of Zn-**8-O^{2,7,12}**. 25

Figure S31. HR-MS Spectrum (ESI+, 100% CH_3CN, TOF) of **Zn-8-O^{2,7,12}**. 26

Figure S32. ^{1}H NMR spectrum (400 MHz, methanol-d$_4$ at 25 °C) of **Zn(II)-8-O^{2,7,18}**. 26

Figure S33. ^{13}C NMR spectrum (101 MHz, methanol-d$_4$ at 25 °C) of **Zn(II)-8-O^{2,7,18}**. 27

Figure S34. UV-vis (CH_2Cl_2) absorption (solid line) and fluorescence emission (CH_2Cl_2) spectra (broken line) of **Zn(II)-8-O^{2,7,18}** 27

Figure S35. FT-IR Spectrum (neat, ATR) of **Zn(II)-8-O^{2,7,18}**. 28

Figure S36. HR-MS Spectrum (ESI+, 100% CH_3CN, TOF) of **Zn(II)-8-O^{2,7,18}** 28

Halochromic Properties of Trioxopyrrocorphins 29

Figure S37. Hill plots for the acid (TFA) titrations of (a) **8-O^{2,7,12}**, (b) **8-O^{2,7,18}**, (c) **8-O^{2,7,13}**, and (d) **8-O^{2,8,18}** in CH_2Cl_2.. 29

Figure S38. Plot showing total [TFA] to reach 50% protonation for (a) **8-O^{2,7,12}**, (b) for **8-O^{2,7,18}**, (c) **8-O^{2,7,13}** and (d) **8-O^{2,8,18}**. 30

Figure S39. UV-Vis (CH_2Cl_2, blue traces and CH_2Cl_2 + TBAOH, red traces) of the mono-oxo and dioxochlorins indicated 30

Figure S40. Photographs of the cuvettes containing the solutions indicated, before and after the addition of acid/base. 31

Figure S41. Comparison of the computed UV-vis spectra for the neutral (solid trace) and bisprotonated (broken trace) triketones indicated 32

Figure S42. Comparison of the computed UV-vis spectra for the neutral (solid trace) and de-protonated at pyrrole (dotted trace), deprotonated at pyrroline (broken trace), and bis-deprotonated (dashed trace) triketones indicated. 33

Figure S43. Comparison of the computed UV-vis spectrum (broken line) for the mono-deprotonated (at pyrrolone) species of the triketone indicated with the experimental spectrum at high [OH$^-$] (solid line). 34

Figure S44. Comparison of the computed UV-vis spectrum (broken line) for the bis-protonated species of the triketone indicated with the experimental spectrum at high [TFA] (solid line). 35

Figure S45. ^{1}H NMR spectral titration of **8-O^{2,7,12}** (0.024 M) with TBAOH (0.166 M). 36

Figure S46. ^{1}H NMR spectral titration of **8-O^{2,7,18}** (0.021 M) with TBAOH (0.091 M). 36

X-Ray Crystallography 37

Details to the X-Ray Crystal Structure of 8-O^{2,7,18} 38

Figure S47. Probability ellipsoid representation of triketone **8-O^{2,7,18}** oblique view. 39

Table S1. Experimental Details to the X-ray Diffraction Analyses of **8-O^{2,7,18}**. 39

Details to the X-Ray Crystal Structure of 8-O^{2,7,18}Zn·pyridine 41

Table S2. Experimental Details to the X-ray Diffraction Analyses of **8-O^{2,7,18}Zn·pyridine**. 42

Figure S48. Probability ellipsoid representation of **8-O^{2,7,18}Zn·pyridine** oblique views. 43

Details to the X-Ray Crystal Structure of 8-O^{2,7,12}Zn·pyridine 44

Table S3. Experimental Details to the X-ray Diffraction Analyses of **8-O^{2,7,12}Zn·pyridine**. 44

Figure S49. Probability ellipsoid representation of **8-O^{2,7,12}Zn·pyridine** top view. 46

Computational Methods 48

Table S4. Calculated NICS(0) and NICS(1) values for the trioxopyrrocorphins. 44

Figure S50. Overlay of the full computed structures of the triketones indicated and the 16-membered inner-inner-inner-inner resonance structure compound in which all atoms outside this macrocycle were replaced with hydrogen atoms. 51

Experimental Section

Materials: Solvents and reagents were used as received. Aluminum-backed, silica gel 60, 250 μm thickness analytical plates, 20 × 20 cm, glass-backed, silica gel 60, 500 μm thickness preparative TLC plates, and standard grade, 60 Å, 32-63 μm flash column silica gel were used for purifications.

Instrumentations: ^{1}H NMR and ^{13}C NMR spectra were recorded using Bruker AVANCE III 400 and 500 MHz spectrometers. IR spectra were recorded from neat material on a Bruker Alpha FTIR spectrometer using an attenuated total reflection (ATR) diamond crystal. Low- and high-resolution mass spectra were recorded using AB Sciex API 2000 Triple Quadrupole and AB Sciex QStar Elite Quadrupole-TOF MS instruments, respectively in CH_3CN.

UV-Vis and Fluorescence Measurements: UV-Vis data were obtained on Varian Cary 100 or Cary 50 spectrophotometers in CH_2Cl_2. The fluorescence spectra were recorded on a Cary Eclipse fluorimeter. Quantum yields (ϕ_f) were determined relative to that of *meso*-tetraphenylporphyrin H_2TPP ($\phi_f = 0.13$ in CH_2Cl_2);[1] $\lambda_{excitation} = \lambda_{Soret}$.

A Hill plot for acid-base titrations was constructed by plotting $\log[(A_i - A_0)/(A_f - A_i)]$ against $\log[OH^-]$, where A_0 and A_i are the absorbance values of the pyrrocorphin and the pyrrocorphin-base complex, respectively, at a given concentration of the base added. Note that A_f indicates the absorbance of fully deprotonated pyrrocorphin at a particular wavelength.

Procedures

[1] Taniguchi, M.; Lindsey, J. S.; Bocian, D. F.; Holten, D., *J. Photochem. Photobiol., C* **2021,** *46*, 100401.

Trioxopyrrocorphins 8-O^{2,7,12} and 8-O^{2,7,18}: Prepared from **OEP** (2 g, 3.74 × 10^{-3} mol) in H_2SO_4 (200 mL) with 3% H_2O_2 (36 mL) as described by Inhoffen and Nolte or Chang.[2]

3,3,8,8,13,13,17,18-Octaethyl-2,7,12-trioxopyrrocorphins (8-O^{2,7,12}): Chromatography condition: silica gel/hexanes-CH_2Cl_2 (40:60 v/v) followed by 100% CH_2Cl_2. The product was isolated as blue solid in 5.5% yield (119 mg, 2.04 × 10^{-4} mol). MW = 582.7754 g/mol. R_f = 0.62 (silica-CH_2Cl_2). ^{1}H NMR (400 MHz, $CDCl_3$): δ 8.93 (s, 1H, *meso*-H), 8.13 (s, 1H, *meso*-H), 8.06 (s, 1H, *meso*-H), 7.82 (s, 1H, *meso*-H), 3.63–3.50 (m, 4H, -CH_2), 2.56–2.35 (m, 12H, -CH_2), 2.14 (s, 1H, NH), 1.86 (s, 1H, NH), 1.64–1.58 (m, 6H, -CH_3), 0.60–0.52 (m, 12H, -CH_3), 0.44 (t, $^3J_{H,H}$ = 7.5 Hz, 6H) ppm. ^{13}C{^{1}H} NMR (101 MHz, $CDCl_3$): δ 209.6, 209.0, 205.5, 167.3, 157.5, 154.6, 150.6, 143.0, 138.3, 134.4, 134.2, 133.0, 130.4, 129.0, 102.0, 98.5, 90.7, 85.3, 71.9, 60.9, 59.3, 57.3, 31.8, 31.5, 30.5, 19.3, 18.8, 18.1, 8.8, 8.7, 8.5 ppm. UV-vis (CH_2Cl_2) λ_{max} (log ε) 408 (4.76), 426 (4.79), 561 (sh), (3.90), 604 (4.13), 630 (sh), (4.08), 682 (4.14) nm. Fluorescence (CH_2Cl_2, $\lambda_{excitation}$ = 426 nm) $\lambda_{max\text{-}emission}$ = 697, 756 (sh) nm; ϕ_f = 0.155. IR (diamond ATR, neat) 1705 ($\nu_{C=O}$), 3303, 3400 ($\nu_{N\text{-}H}$) cm^{-1}. HR-MS (ESI+, 100% CH_3CN, TOF): *m*/*z* calc'd for $C_{36}H_{46}N_4O_3$ [M]$^+$ 582.3570, found 582.3520; calc'd for $C_{36}H_{47}N_4O_3$ [M+H]$^+$ 583.3643, found 583.3588; calc'd for $C_{36}H_{46}N_4O_3Na$ [M+Na]$^+$ 605.3462, found 605.3366.

3,3,8,8,12,13,17,17-Octaethyl-2,7,18-trioxopyrrocorphin (8-O^{2,7,18}): Chromatography condition: silica gel/CH_2Cl_2. The product was isolated as green crystalline solid in 2% yield (43 mg, 7.37 × 10^{-5} mol). MW = 582.7754 g/mol. R_f = 0.21 (silica-CH_2Cl_2). ^{1}H NMR (400 MHz, $CDCl_3$): δ 9.02 (s, 1H, *meso*-H), 8.60 (s, 1H, *meso*-H), 8.52 (s, 1H, *meso*-H), 8.25 (s, 1H, *meso*-

[2] (a) Inhoffen, H. H.; Nolte, W., *Justus Liebigs Ann. Chem.* **1969,** *725*, 167–176. (b) Chang, C. K., *Biochemistry* **1980,** *19* (9), 1971-1976.

H), 3.76–3.68 (m, 4H, -CH_2), 2.63 –2.45 (m, 12H, -CH_2), 1.68 (t, $^3J_{H,H}$ = 7.5 Hz, 6H, -CH_3), 0.55–0.46 (m, 18H, -CH_3), 0.20 (s, 1H NH), -0.06 (s, 1H, NH) ppm. $^{13}C\{^1H\}$ NMR (101 MHz, $CDCl_3$): δ 209.9, 208.8, 204.6, 159.6, 158.4, 152.2, 149.1, 149.0, 137.7, 135.9, 135.3, 133.5, 130.1, 99.2, 96.5, 90.1, 86.1, 60.2, 59.1, 58.4, 31.9, 31.6, 31.5, 19.2, 19.1, 18.4, 18.2, 8.7, 8.4 ppm. UV-vis (CH_2Cl_2) λ_{max} (log ε): 425 (5.57), 458 (5.33), 623 (4.93), 665 (4.77), 689 (4.43), 727 (5.44) nm. Fluorescence (CH_2Cl_2, $\lambda_{excitation}$ = 458 nm) $\lambda_{max\text{-}emission}$ = 639, 778 (sh) nm; ϕ_f = 0.085. IR (diamond ATR, neat): 1698 ($\nu_{C=O}$), 3287, 3393 ($\nu_{N\text{-}H}$) cm^{-1}. HR-MS (ESI+, 100% CH_3CN, TOF): calc'd for $C_{36}H_{46}N_4O_3$ $[M]^+$ 582.3570, found 582.3515; calc'd for $C_{36}H_{47}N_4O_3$ $[M+H]^+$ 583.3643, found 583.3586; calc'd for $C_{36}H_{46}N_4O_3Na$ $[M+Na]^+$ 605.3462, found 605.3398.

3,3,8,8,12,12,17,18-Octaethyl-2,7,13-trioxopyrrocorphin (8-O^{2,7,13}): Prepared in 13% yield (3.4 mg, 6.0 × 10^{-6} mol) by oxidation of dioxoisobacteriochlorin isomer **6-O^{2,8}** (25 mg, 4.41 × 10^{-5} mol) in H_2SO_4/H_2O_2 using a similar procedure as for oxidation of **OEP**,[2] except that twice the amount of 3% H_2O_2 was used. The product was isolated as purple solid. Preparative TLC conditions: silica gel/hexanes-CH_2Cl_2 (20:80 v/v). MW = 582.7754 g/mol. R_f = 0.29 (silica-CH_2Cl_2). ^1H NMR (400 MHz, $CDCl_3$): δ 8.56 (s, 1H, *meso*-H), 8.48 (s, 1H, *meso*-H), 7.69 (s, 1H, *meso*-H), 6.95 (s, 1H, *meso*-H), 4.01 (s, 1H, -NH), 3.62 (s, 1H, -NH), 3.41 (q, $^3J_{H,H}$ = 7.1 Hz, 4H, -CH_2), 2.47 (dd, $^3J_{H,H}$ = 13.9, 7.3 Hz, 2H, -CH_2), 2.37–2.16 (m, 12H, -CH_2), 1.54 (d, $^3J_{H,H}$ = 7.2 Hz, 6H, -CH_3), 0.64 (t, $^3J_{H,H}$ = 7.3 Hz, 6H, -CH_3), 0.50 (dt, $^3J_{H,H}$ = 14.5, 7.4 Hz, 12H, -CH_3) ppm. $^{13}C\{^1H\}$ NMR (101 MHz, $CDCl_3$): δ 208.8, 208.7, 205.2, 171.8, 169.7, 151.4, 144.0, 143.4, 137.7, 136.3, 134.5, 132.3, 130.9, 103.3, 100.7, 90.5, 88.1, 60.9, 60.6, 58.4, 31.9, 30.5, 30.2, 29.8, 18.6, 18.5, 17.9, 17.8, 8.9, 8.8, 8.4 ppm. UV-vis (CH_2Cl_2) λ_{max} (log ε): 376 (4.94), 412 (4.97), 525 (4.18), 560 (4.47), 601 (4.17), 659 (4.29) nm. Fluorescence (CH_2Cl_2, $\lambda_{excitation}$ = 412

nm) $\lambda_{max-emission}$ = 688; ϕ_f = 0.112. HR-MS (ESI+, 100% CH_3CN, TOF): calc'd for $C_{36}H_{46}N_4O_3$ $[M]^+$ 582.3570, found 582.3800.

3,3,7,7,12,13,17,17-Octaethyl-2,8,18-trioxopyrrocorphin (8-O^{2,8,18}): Prepared as a brownish solid in 11% yield (3 mg, 5.14 × 10^{-6} mol) from an H_2SO_4/H_2O_2 oxidation of dioxoisobacteriochlorin isomer **6-O^{2,8}** (25 mg, 4.41 × 10^{-5} mol) using a similar procedure as for the oxidation of **OEP**,[2] except that twice the amount of 3% H_2O_2 was used. Preparative TLC conditions: silica gel/hexanes-CH_2Cl_2 (20:80 v/v). MW = 582.7754 g/mol. R_f = 0.12 (silica-CH_2Cl_2). ^{1}H NMR (400 MHz, $CDCl_3$): δ 8.86 (s, 1H, *meso*-H), 8.59 (s, 1H, *meso*-H), 8.22 (s, 1H, *meso*-H), 7.33 (s, 1H, *meso*-H), 3.56 (dq, $^3J_{H,H}$ = 15.2, 7.5 Hz, 4H, -CH_2), 2.55 (dd, $^3J_{H,H}$ = 13.9, 7.3 Hz, 2H, -CH_2), 2.51–2.24 (m, 12H, -CH_2), 2.15 (s, 1H, -NH), 1.80 (s, 1H, -NH), 1.60 (dt, $^3J_{H,H}$ = 11.2, 7.6 Hz, 6H, -CH_3), 0.59 (t, $^3J_{H,H}$ = 7.4 Hz, 6H, -CH_3), 0.49 (dt, $^3J_{H,H}$ = 15.4, 7.4 Hz, 12H, -CH_3) ppm. $^{13}C\{^1H\}$ NMR (101 MHz, $CDCl_3$): δ 209.1, 208.5, 204.4, 170.0, 157.2, 152.3, 150.5, 144.0, 137.0, 136.2, 133.1, 132.7, 132.3, 100.9, 99.1, 88.9, 88.0, 60.6, 59.1, 58.0, 32.0, 31.4, 30.7, 29.8, 18.9, 18.8, 18.2, 18.0, 8.7, 8.4 ppm. UV-vis (CH_2Cl_2) λ_{max} (log ε): 379 (5.08), 411 (5.17), 493 (3.72), 527 (3.95), 565 (4.27), 658 (4.32), 724 (4.84) nm. Fluorescence (CH_2Cl_2, $\lambda_{excitation}$ = 411 nm) $\lambda_{max-emission}$ = 742; ϕ_f = 0.178. HR-MS (ESI+, 100% CH_3CN, TOF): calc'd for $C_{36}H_{46}N_4O_3$ $[M]^+$ 582.3570, found 582.3492.

[3,3,8,8,13,13,17,18-Octaethyl-2,7,12-trioxopyrrocorphinato]zinc(II) (Zn-8-O^{2,7,12}): Free base trioxopyrrocorphin **8-O^{2,7,12}** (120 mg, 2.06 × 10^{-4} mol), $ZnCl_2$.·$2H_2O$ (281 mg, 2.06 × 10^{-3} mol, 10 equiv.) and Li_2CO_3 (60 mg, 8.12 × 10^{-4} mol, 4 equiv.) were dissolved in DMF (25 mL) and heated to reflux for 90 min. The solution was cooled to room temperature and then diluted with DI water (~200 mL) followed by microfiltration. The crude compound was purified by column

chromatography (CH_2Cl_2 followed by 7% acetone in CH_2Cl_2). The metal complex was isolated as a green powder in 94% (125 mg, 1.93×10^{-4} mol) yield. MW = 646.1396 g/mol. $R_f = 0.89$ (silica-10% acetone in CH_2Cl_2). ^{1}H NMR (400 MHz, DMSO-d$_6$): δ 8.94 (s, 1H, *meso*-H), 8.47 (s, 1H, *meso*-H), 8.20 (s, 1H, *meso*-H), 8.10 (s, 1H, *meso*-H), 3.60 (dd, $^3J_{H,H}$ = 7.0, 3.0 Hz, 4H, -CH_2), 2.62 - 2.51 (m, 4H, -CH_2), 2.48 - 2.43 (m, 2H, -CH_2), 2.36 (dt, $^3J_{H,H}$ = 12.0, 6.5 Hz, 6H, -CH_2), 1.57 (dt, $^3J_{H,H}$ = 11.5, 7.5 Hz, 6H, -CH_3), 0.42 (t, $^3J_{H,H}$ = 7.5 Hz, 6H, -CH_3), 0.32 (dd, $^3J_{H,H}$ = 17.0, 7.5 Hz, 12H, -CH_3) ppm. ^{13}C{^{1}H) NMR (101 MHz, DMSO-d$_6$): δ 209.2, 208.9, 207.2, 161.5, 159.3, 153.7, 146.5, 145.8, 144.1, 141.3, 139.7, 138.0, 137.9, 98.1, 95.9, 88.4, 85.9, 59.5, 58.2, 57.4, 30.4, 30.3, 30.2, 18.6, 18.5, 8.2, 8.1, 8.0 ppm. UV-vis (CH_2Cl_2) λ_{max} (log ε): 432 (4.72), 486 (3.70), 660 (3.95), 708 (4.45) nm. Fluorescence (CH_2Cl_2, $\lambda_{excitation}$ = 432 nm) $\lambda_{max\text{-}emission}$ = 717 nm; ϕ_f = 0.017. IR (diamond ATR, neat): 1705 ($\nu_{C=O}$) cm^{-1}. HR-MS (ESI+, 100% CH_3CN, TOF): calc'd for $C_{36}H_{44}N_4O_3Zn$ [M]$^+$ 644.2705, found 644.2747.

[3,3,8,8,12,13,17,17-Octaethyl-2,7,18-trioxopyrrocorphinato]zinc(II) (Zn-8-O^{2,7,18}): Prepared as a green crystalline solid in 86% yield (67 mg, 1.04×10^{-4} mol) from free base **8-O^{2,7,18}** (70 mg, 1.20×10^{-4} mol), $ZnCl_2{\cdot}2H_2O$ (164 mg, 1.20×10^{-3} mol, 10 equiv.) and Li_2CO_3 (36 mg, 4.80×10^{-4} mol, 4 equiv.) in 15 mL of DMF and using the same work-up protocol as described for **8-O^{2,7,18}**. Column chromatography condition: silica gel/CH_2Cl_2 followed by 10% acetone in CH_2Cl_2. MW = 646.1396 g/mol. $R_f = 0.7$ (silica-10% acetone in CH_2Cl_2). ^{1}H NMR (400 MHz, methanol-d$_4$): δ 9.02 (s, 1H, *meso*-H), 8.60 (s, 1H, *meso*-H), 8.54 (s, 1H, *meso*-H), 8.43 (s, 1H, *meso*-H), 3.70-3.65 (m, 4H, -CH_2), 2.71–2.45 (m, 12H, -CH_2), 1.69 (t, $^3J_{H,H}$ = 7.6 Hz, 6H, -CH_3), 0.48–0.39 (m, 18H, -CH_3) ppm. ^{13}C NMR (101 MHz, CD_3OD): δ 212.3, 210.9, 210.8, 161.8, 160.3, 159.1, 153.9, 151.2, 145.9, 144.3, 142.8, 140.5, 140.2, 97.2, 94.7, 94.3, 90.5, 62.2, 62.1,

61.0, 32.5, 32.4, 32.2, 20.2, 19.0, 18.8, 9.0, 8.9 ppm. UV-vis (CH_2Cl_2) λ_{max} (log ε): 434 (4.66), 477 (4.54), 663 (4.17), 718 (4.88) nm. Fluorescence (CH_2Cl_2, $\lambda_{excitation}$ = 434 nm) $\lambda_{max\text{-}emission}$ = 728, 762 (sh) nm; ϕ_f = 0.020. IR (diamond ATR, neat): 1695 ($\nu_{C=O}$) cm^{-1}. HR-MS (ESI+, 100% CH_3CN, TOF): calc'd for $C_{36}H_{44}N_4O_3Zn$ $[M]^+$ 644.2705, found 644.2558.

Reproduction of Spectra

Free Base Trioxopyrrocorphins

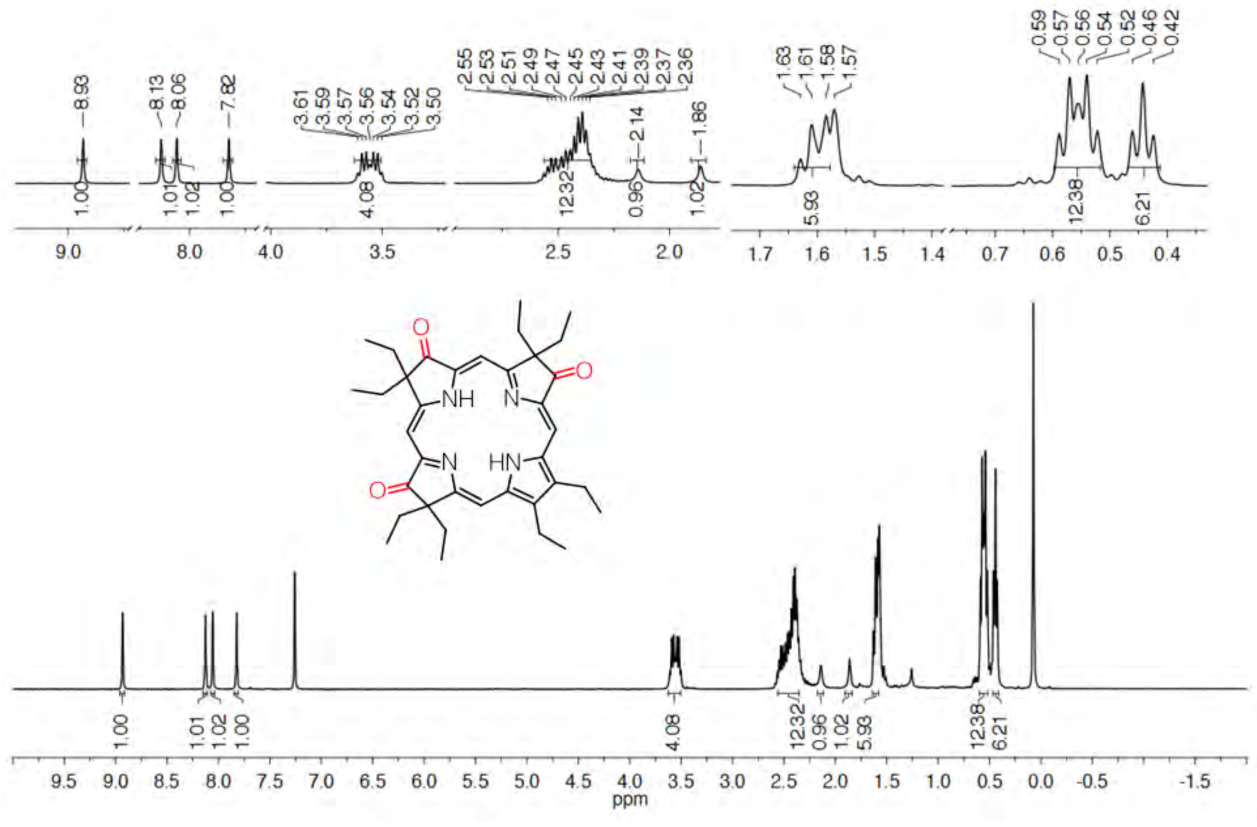

Figure S1. ^1H NMR spectrum (400 MHz, $CDCl_3$ at 25 °C) of **8-O^{2,7,12}**.

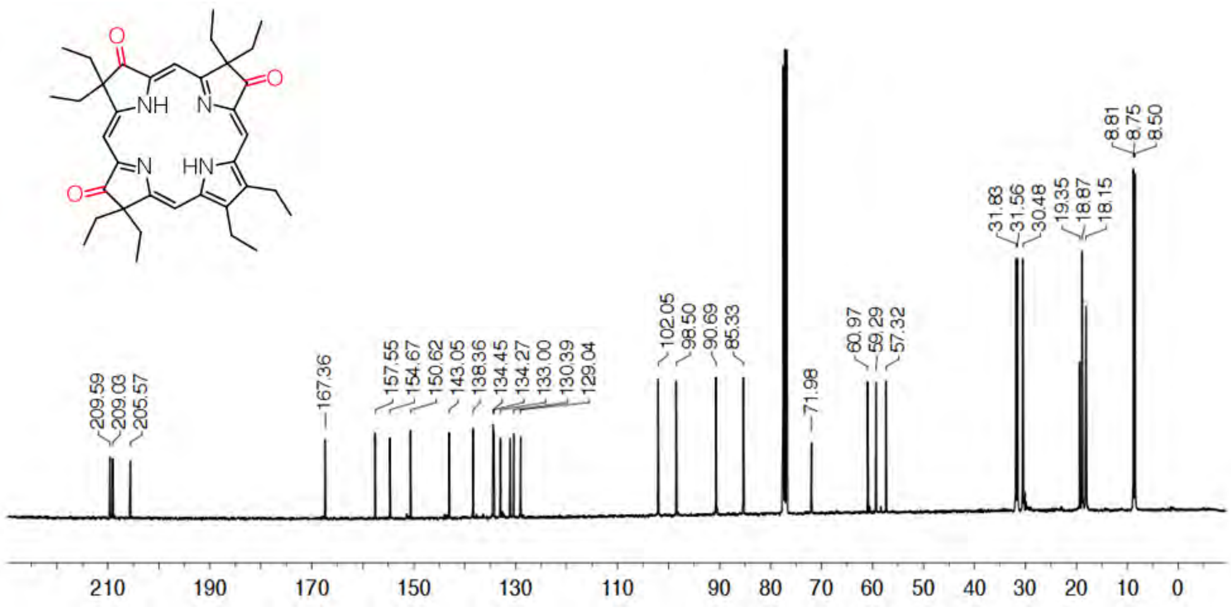

Figure S2. ^{13}C NMR spectrum (125 MHz, $CDCl_3$ at 25 °C) of **8-O^{2,7,12}**.

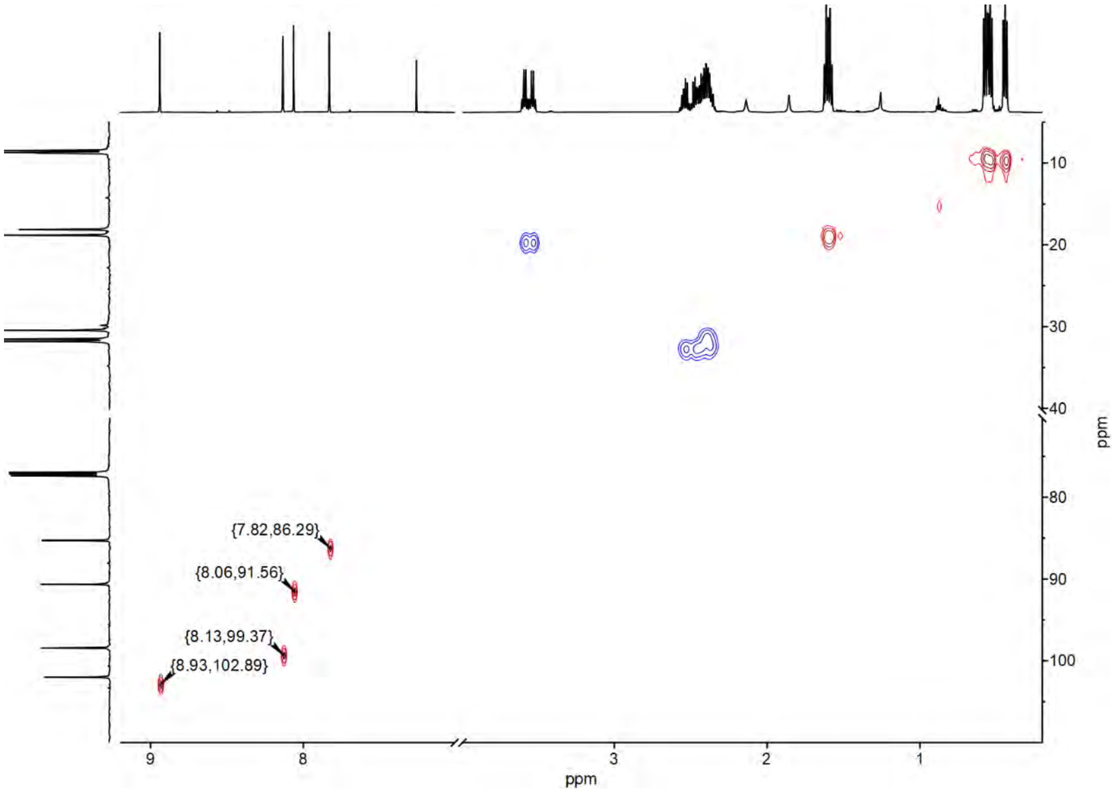

Figure S3. HSQC NMR spectrum ($CDCl_3$, 25 °C) of **8-O^{2,7,12}**.

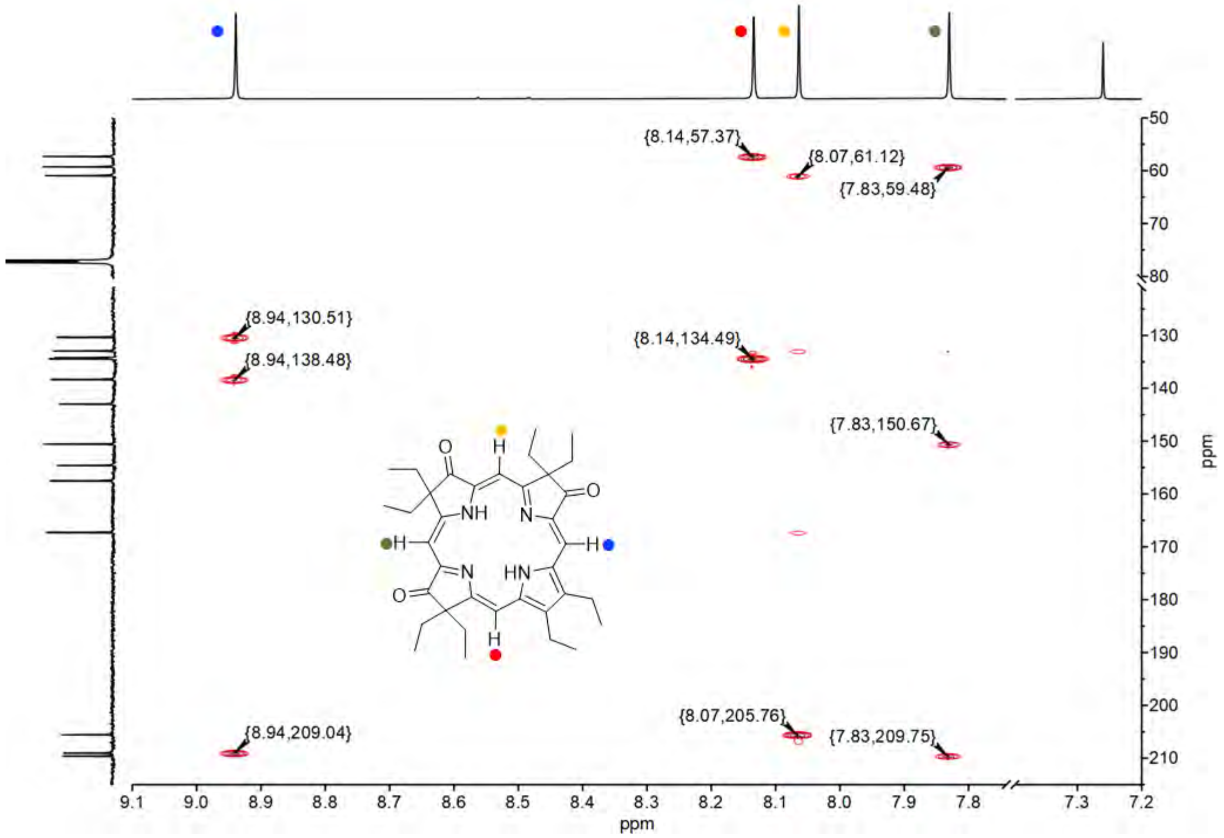

Figure S4. HMBC NMR spectrum ($CDCl_3$, 25 °C) of **8-O^{2,7,12}**.

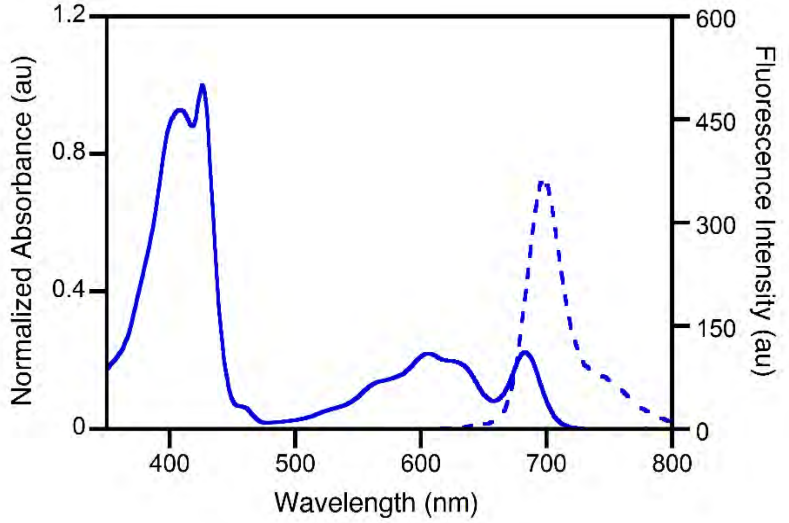

Figure S5. UV-vis (CH_2Cl_2) absorption (solid line) and fluorescence emission (CH_2Cl_2) spectra (broken line) of **8-O^{2,7,12}**.

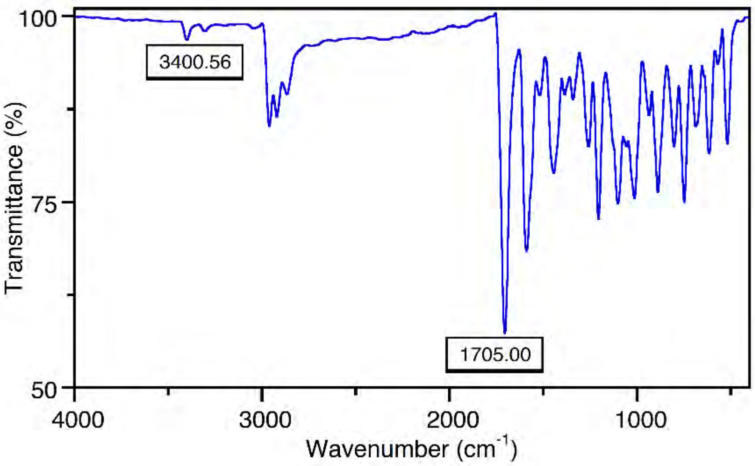

Figure S6. FT-IR Spectrum (neat, ATR) of **8-O**^{2,7,12}.

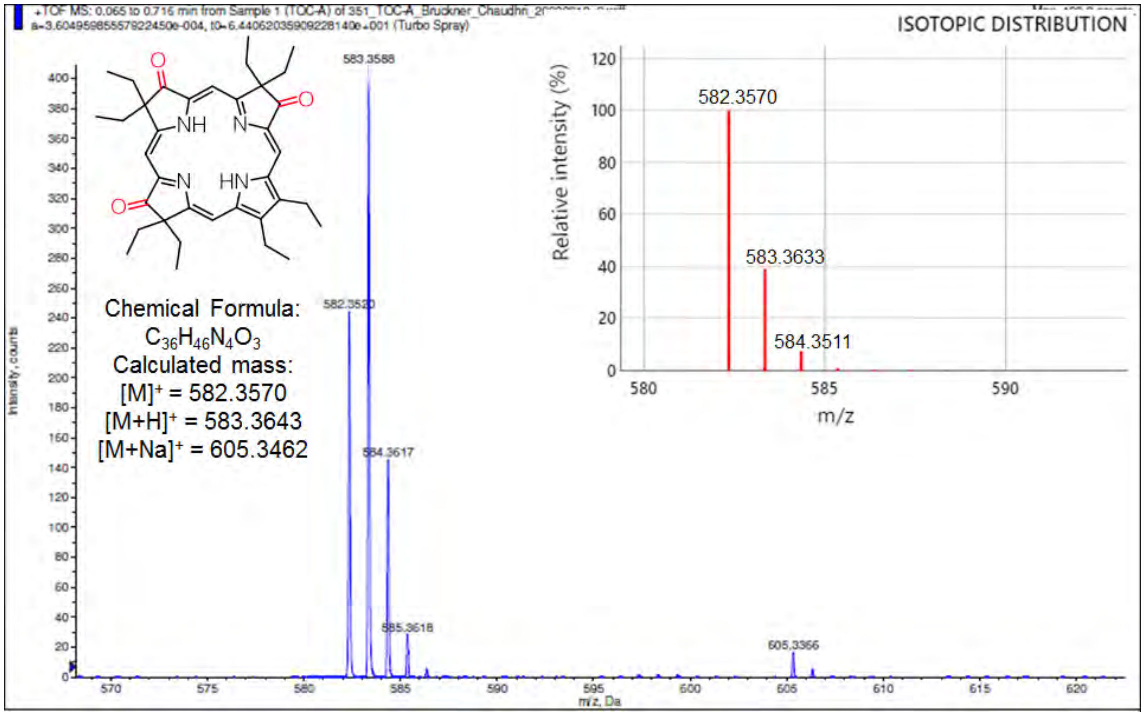

Figure S7. HR-MS Spectrum (ESI+, 100% CH_3CN, TOF) of **8-O**^{2,7,12}.

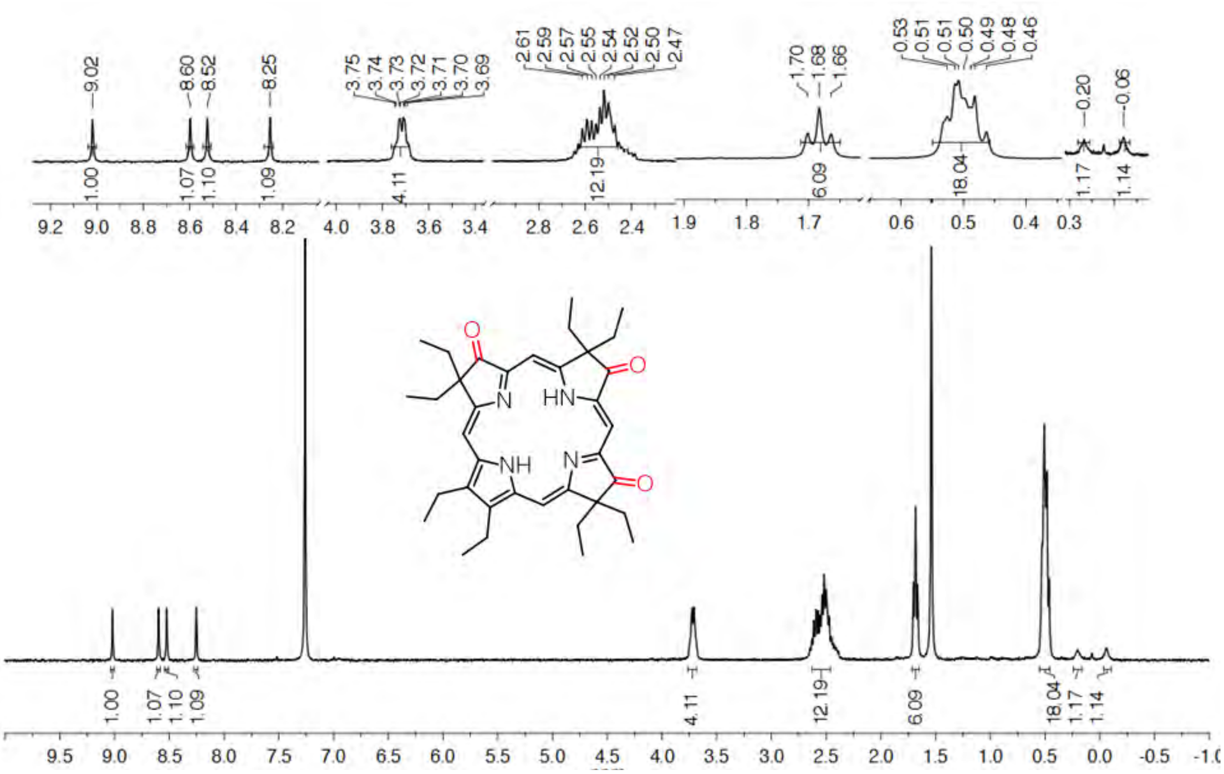

Figure S8. ^{1}H NMR spectrum (400 MHz, $CDCl_3$ at 25 °C) of **8-O^{2,7,18}**.

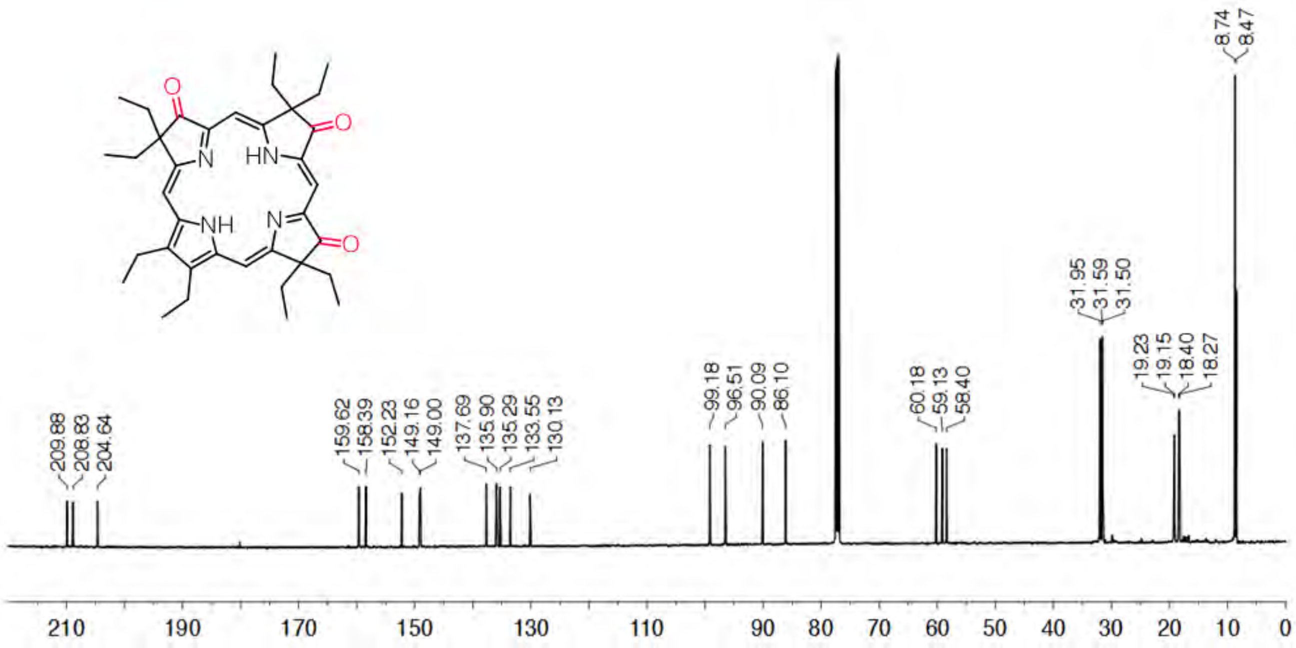

Figure S9. ^{13}C NMR spectrum (125 MHz, $CDCl_3$ at 25 °C) of **8-O^{2,7,18}**.

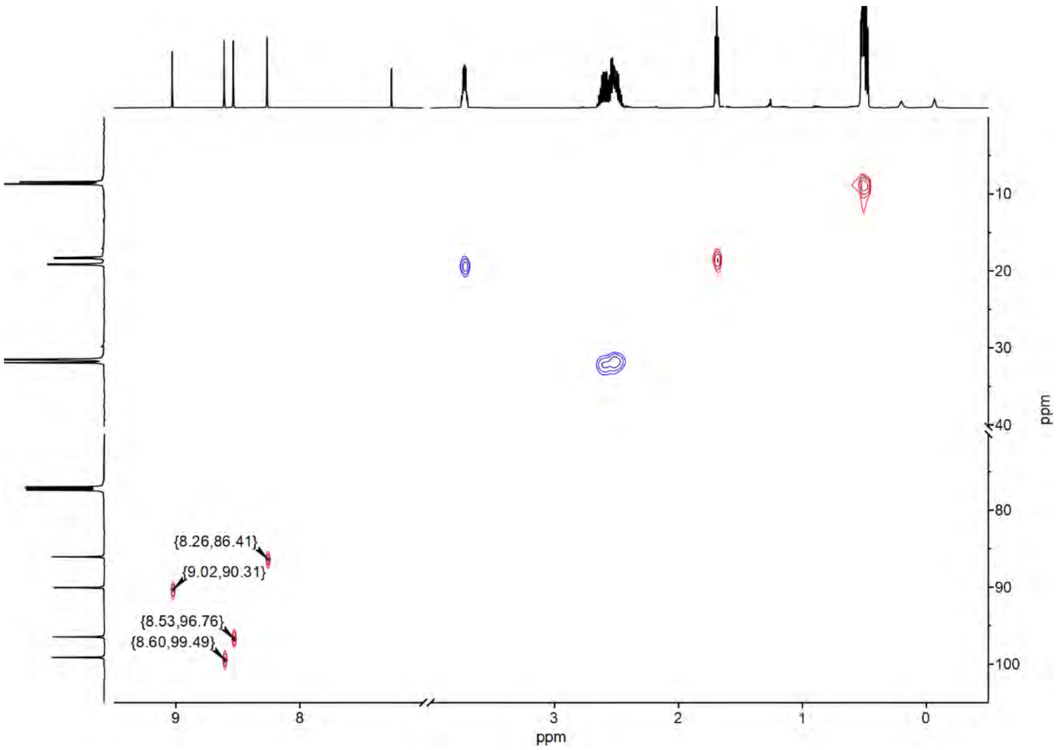

Figure S10. HSQC NMR spectrum ($CDCl_3$, 25 °C) of **8-O^{2,7,18}**.

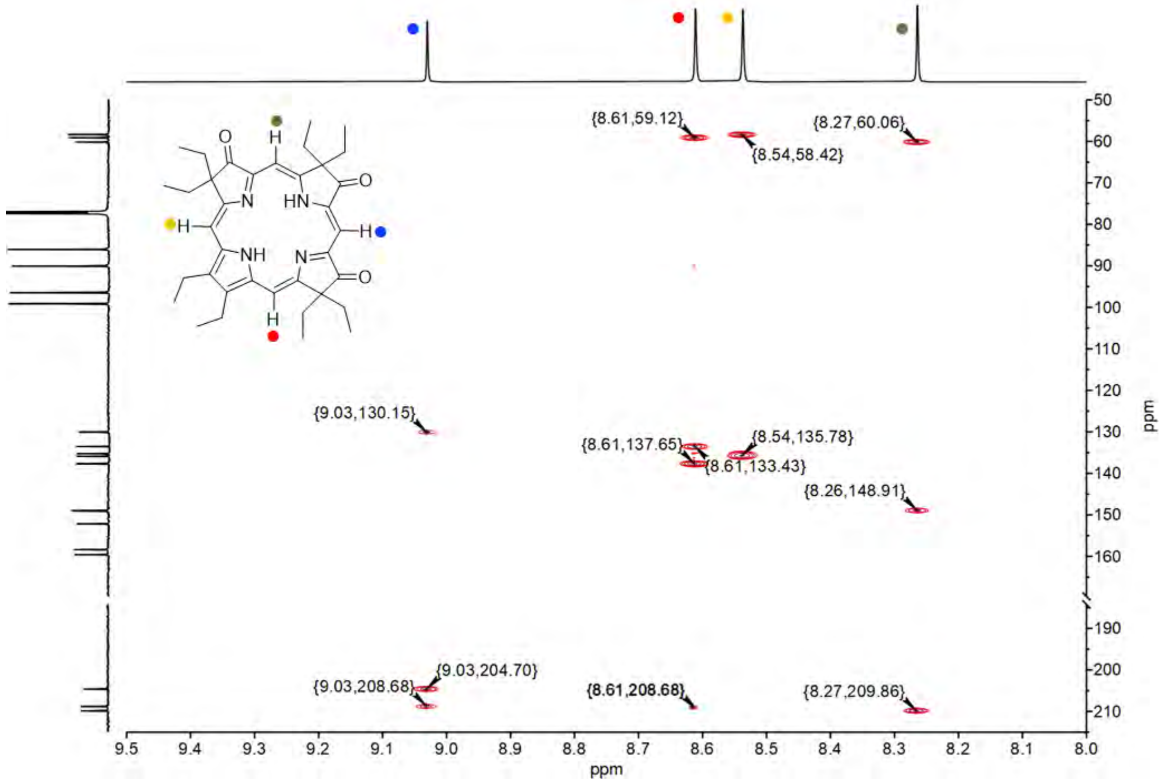

Figure S11. HMBC NMR spectrum ($CDCl_3$, 25 °C) of **8-O^{2,7,18}**.

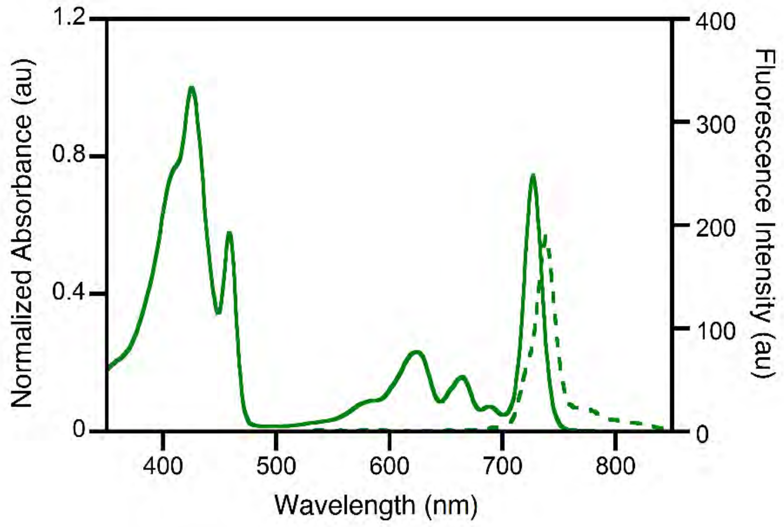

Figure S12. UV-vis (CH_2Cl_2) absorption (solid line) and fluorescence emission (CH_2Cl_2) spectra (broken line) of **8-O^{2,7,18}**.

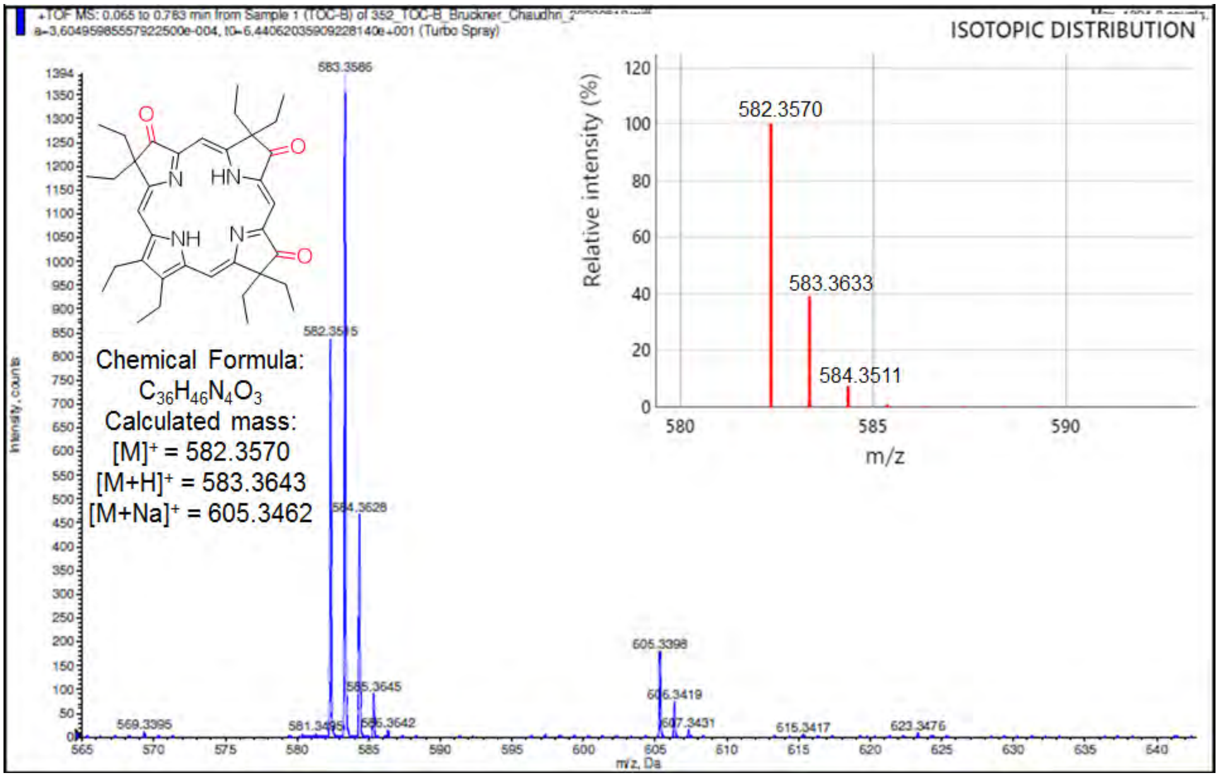

Figure S13. HR-MS Spectrum (ESI+, 100% CH_3CN, TOF) of **8-O^{2,7,18}**.

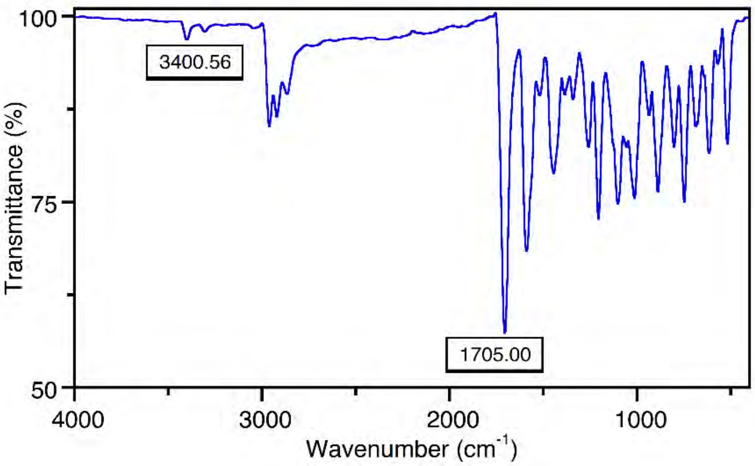

Figure S14. FT-IR Spectrum (neat, ATR) of **8-O^{2,7,18}**.

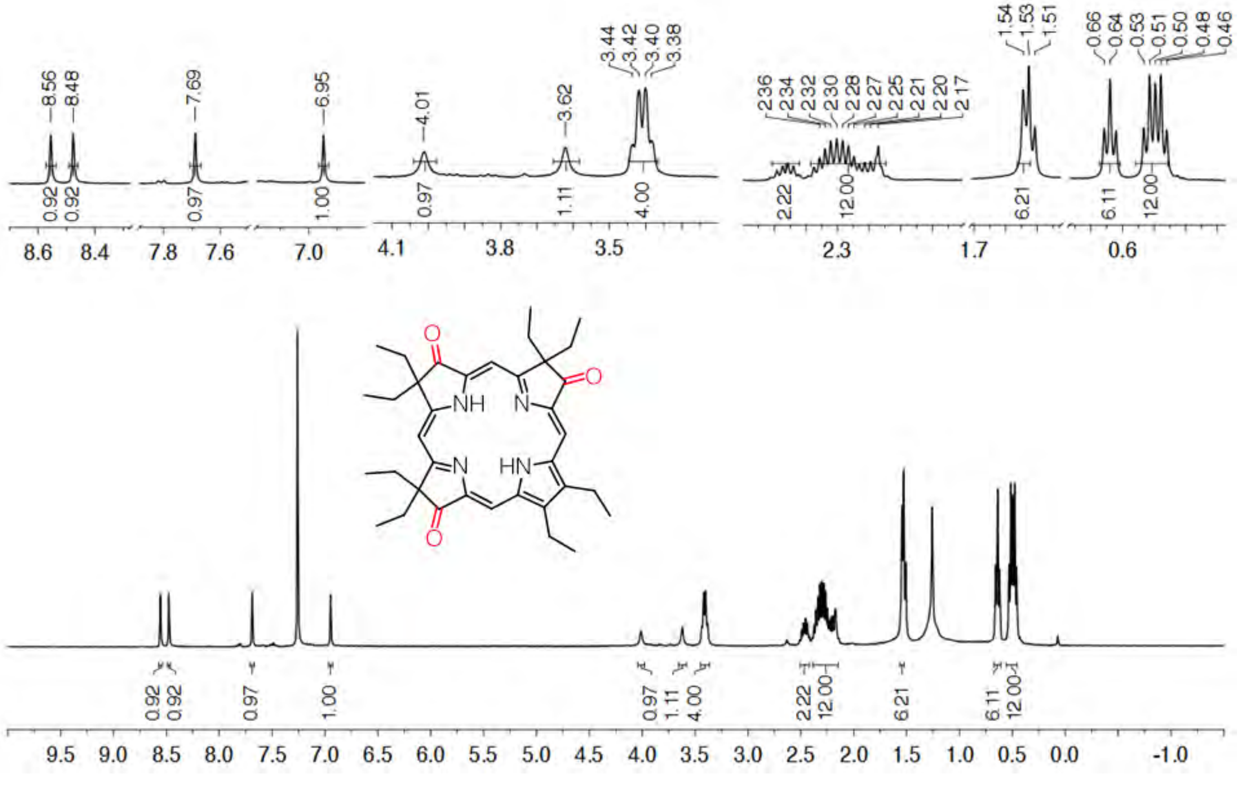

Figure S15. ^{1}H NMR spectrum (400 MHz, $CDCl_3$ at 25 °C) of **8-O^{2,7,13}**.

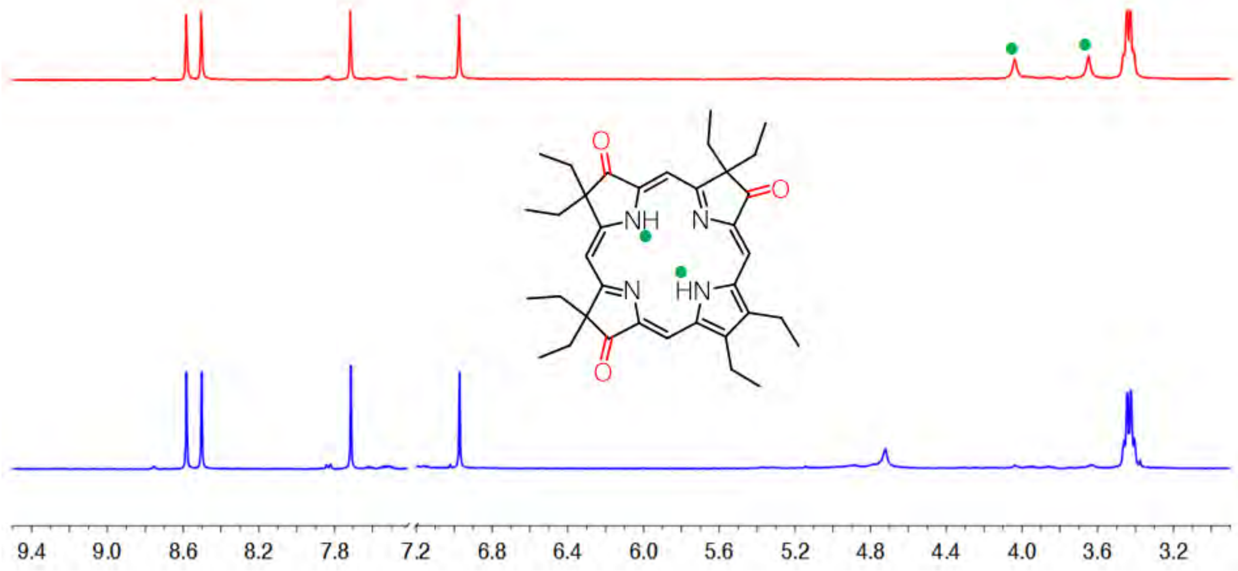

Figure S16. ^{1}H NMR spectrum (400 MHz, DMSO-D$_6$ at 25 °C) of **8-O^{2,7,13}** before (red color) and after (blue color) D_2O exchange.

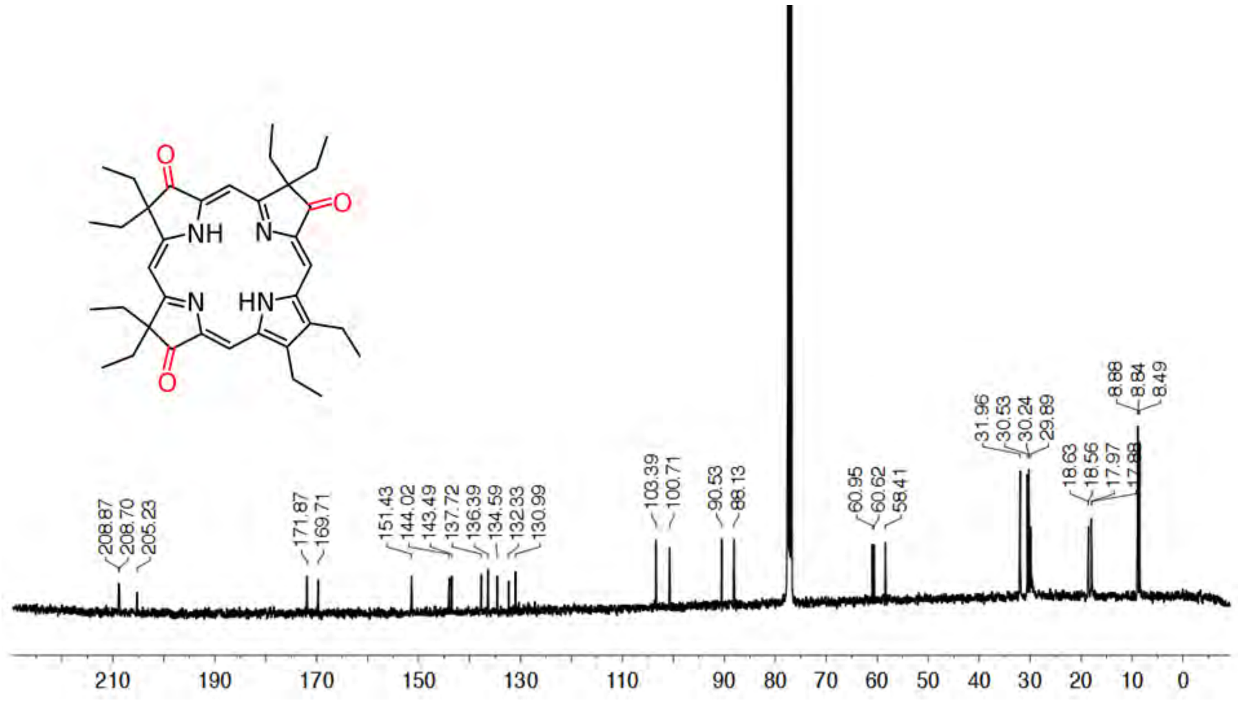

Figure S17. ^{13}C NMR spectrum (125 MHz, $CDCl_3$ at 25 °C) of **8-O^{2,7,13}**.

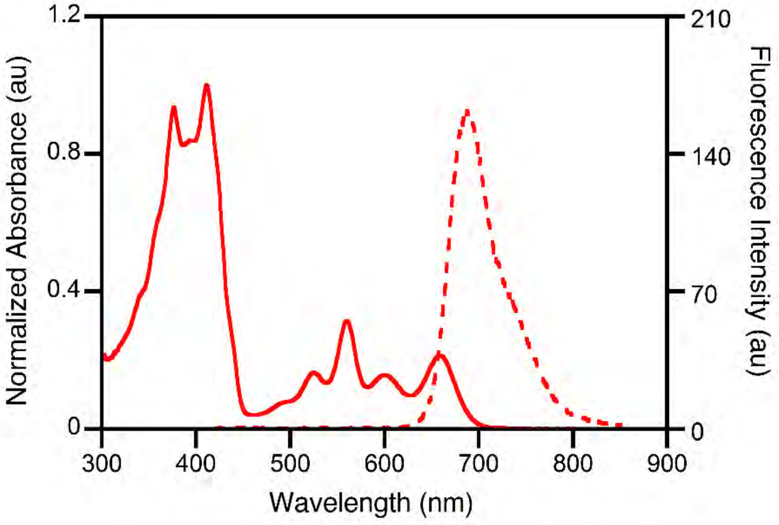

Figure S18. UV-vis (CH_2Cl_2) absorption (solid line) and fluorescence emission (CH_2Cl_2) spectra (broken line) of **8-O^{2,7,13}**.

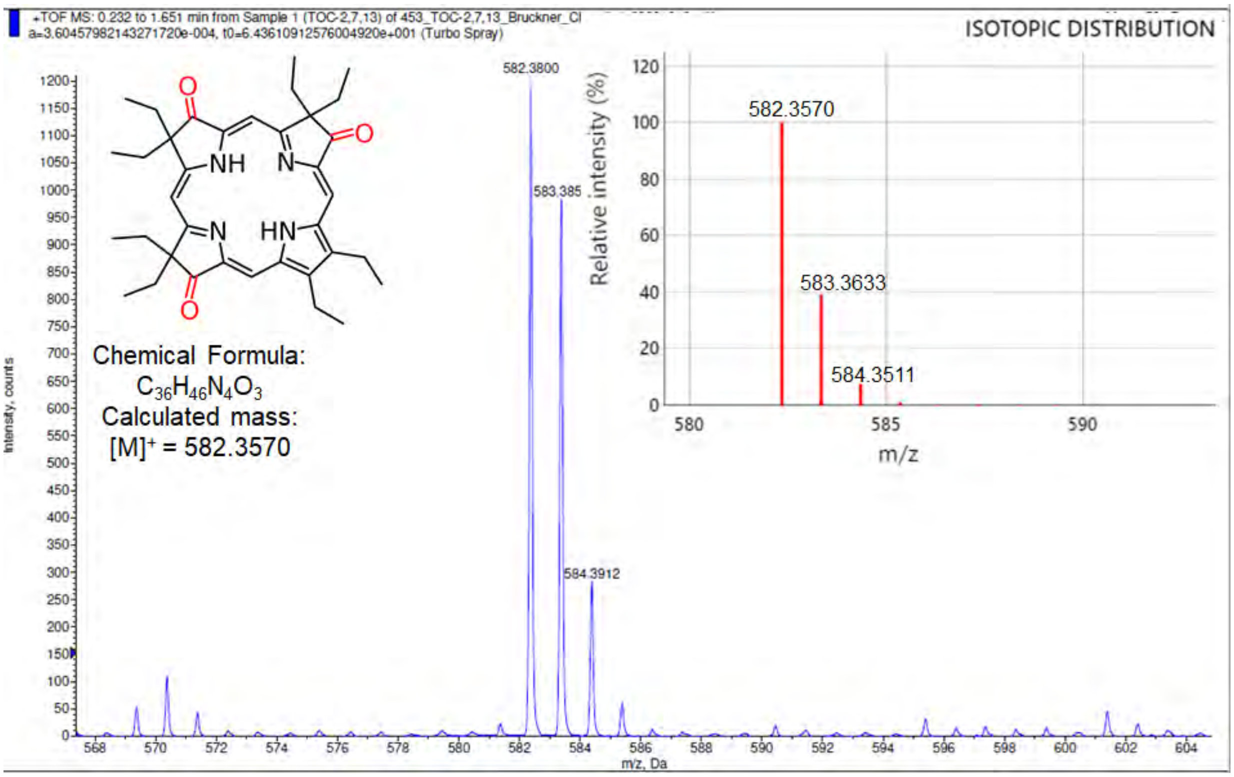

Figure S19. HR-MS Spectrum (ESI+, 100% CH_3CN, TOF) of **8-O^{2,7,13}**.

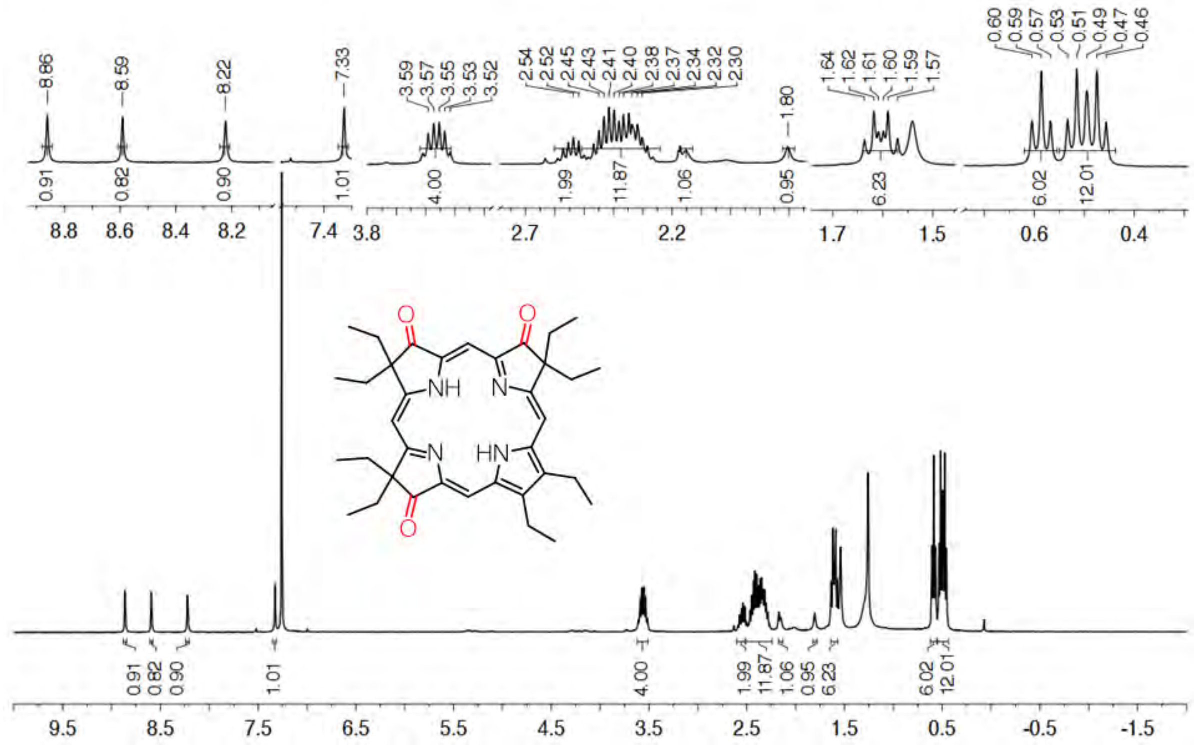

Figure S20. ^1H NMR spectrum (400 MHz, $CDCl_3$ at 25 °C) of **8-O^{2,8,18}**.

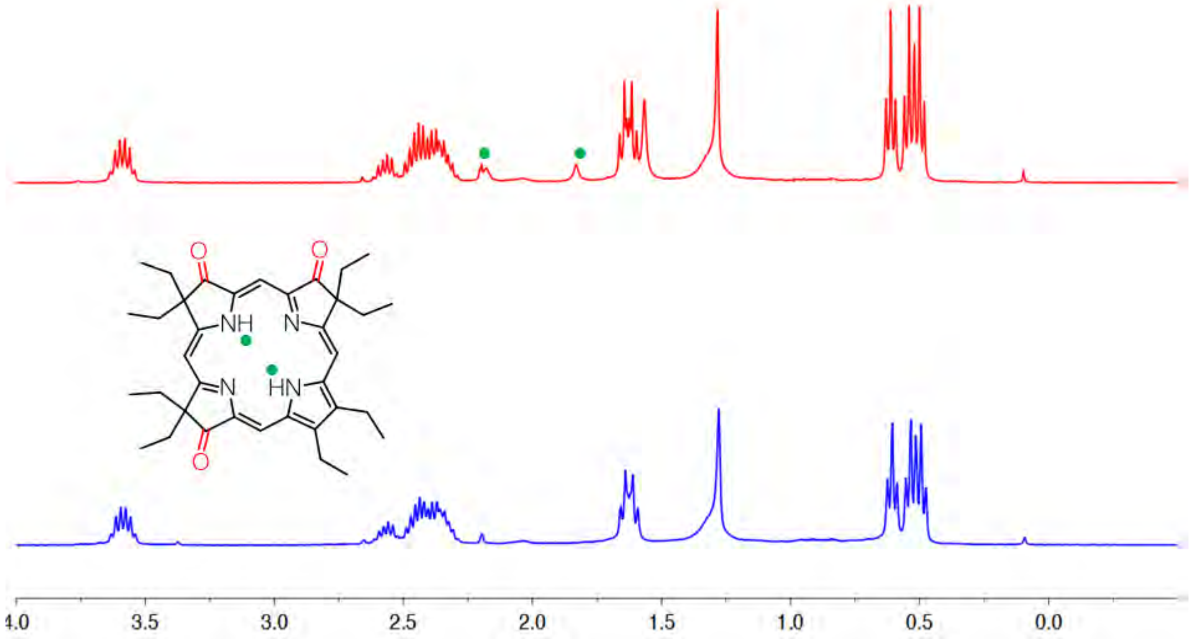

Figure S21. ^1H NMR spectrum (400 MHz, DMSO-D_6 at 25 °C) of **8-O^{2,8,18}** before (red color) and after (blue color) D_2O exchange.

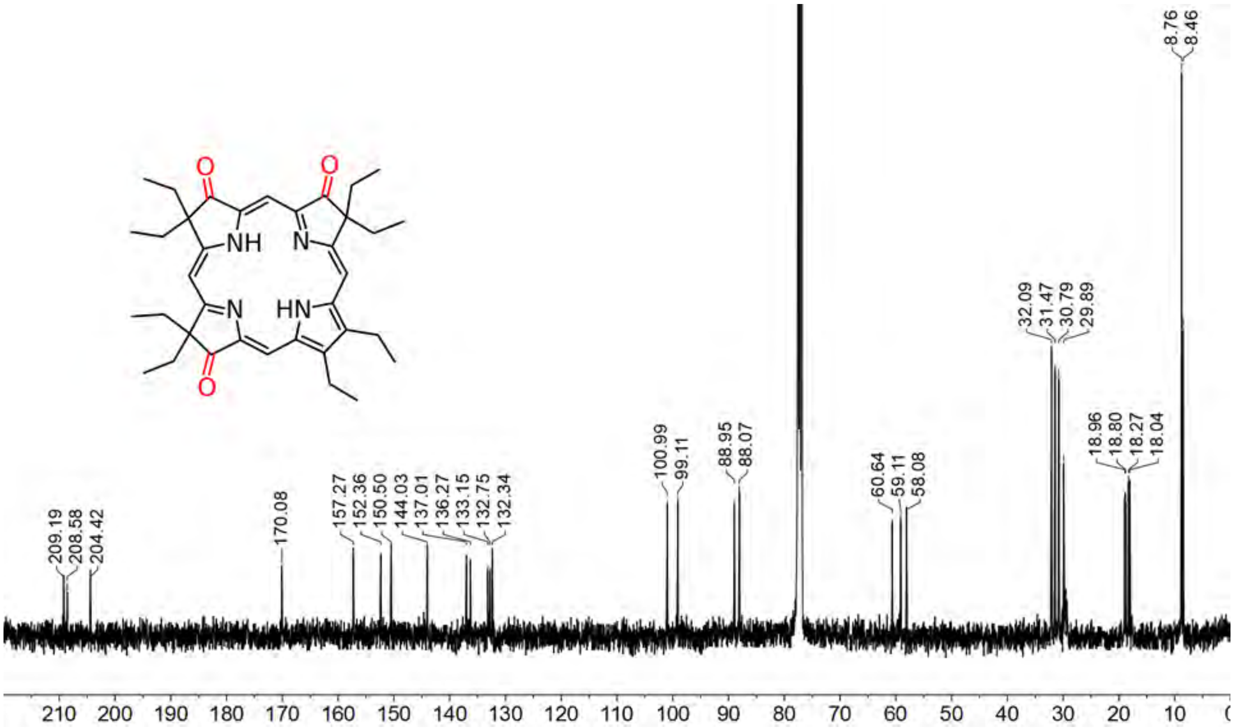

Figure S22. ^{13}C NMR spectrum (125 MHz, $CDCl_3$ at 25 °C) of **8-O^{2,8,18}**.

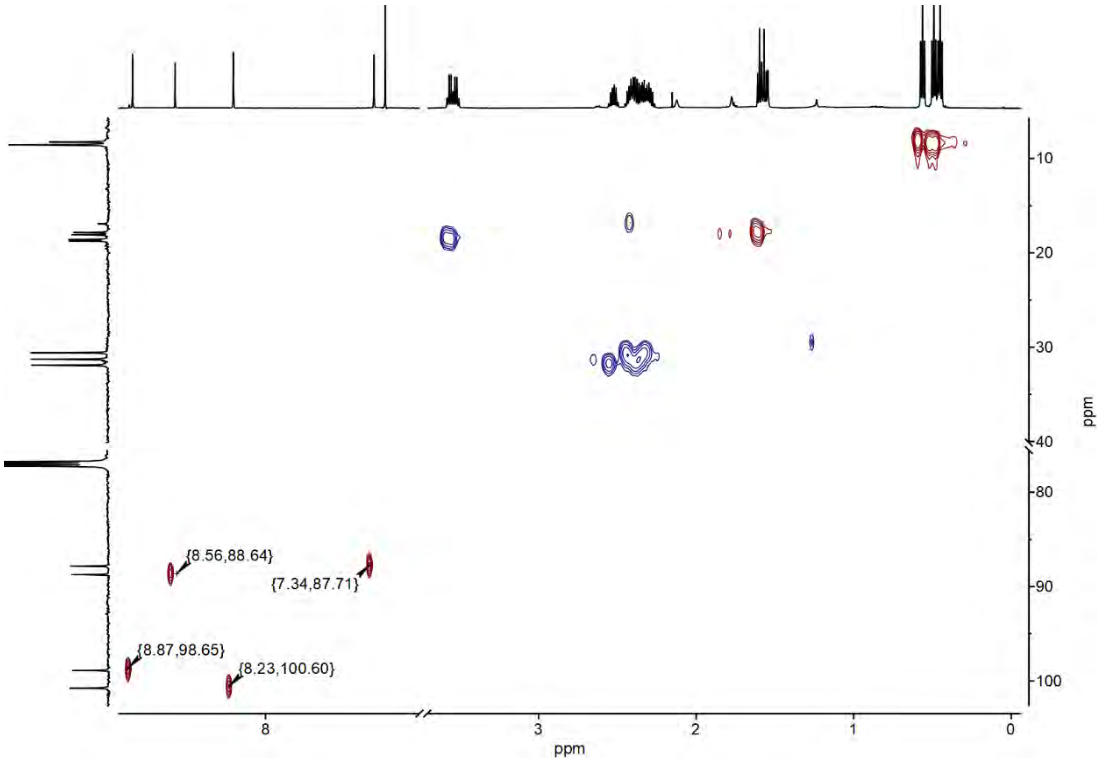

Figure S23. HSQC NMR spectrum ($CDCl_3$, 25 °C) of **8-O^{2,8,18}**.

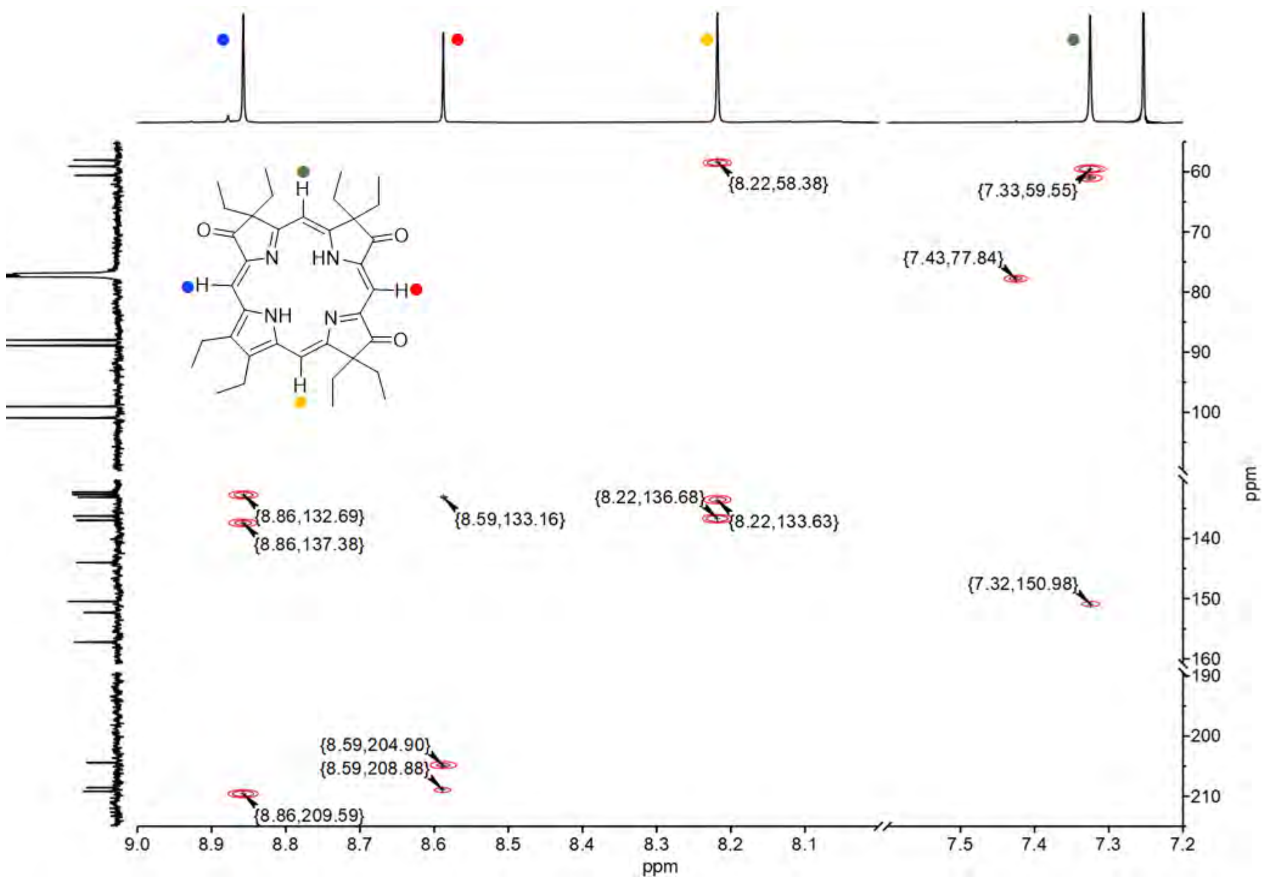

Figure S24. HMBC NMR spectrum ($CDCl_3$, 25 °C) of **8-O^{2,8,18}**.

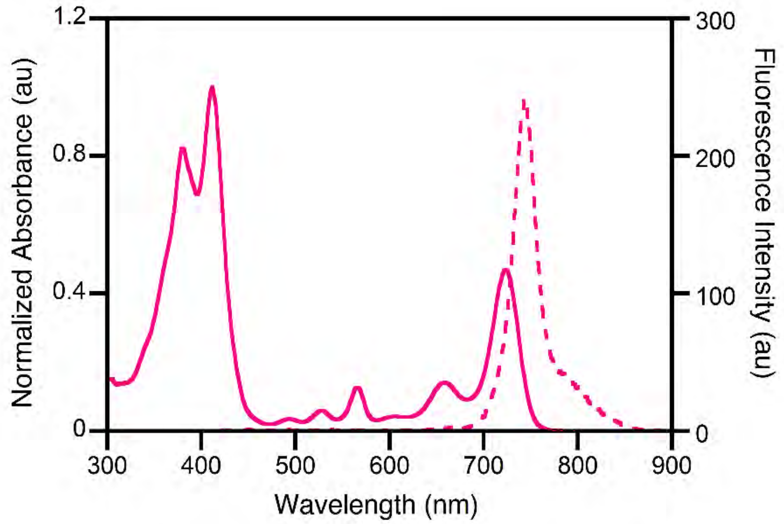

Figure S25. UV-vis (CH_2Cl_2) absorption (solid line) and fluorescence emission (CH_2Cl_2) spectra (broken line) of **8-O^{2,8,18}**.

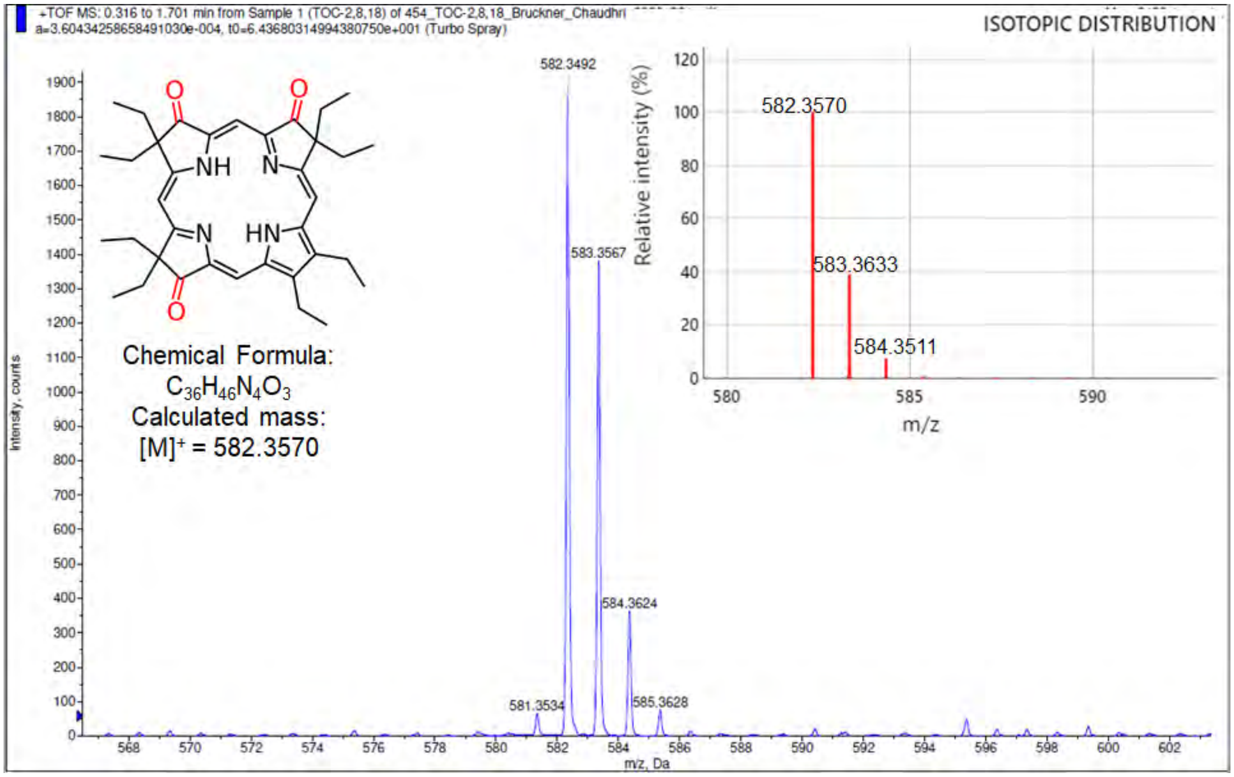

Figure S26. HR-MS Spectrum (ESI+, 100% CH_3CN, TOF) of **8-O**^{2,8,18}.

Zn(II)-Complexes of Trioxopyrrocorphins

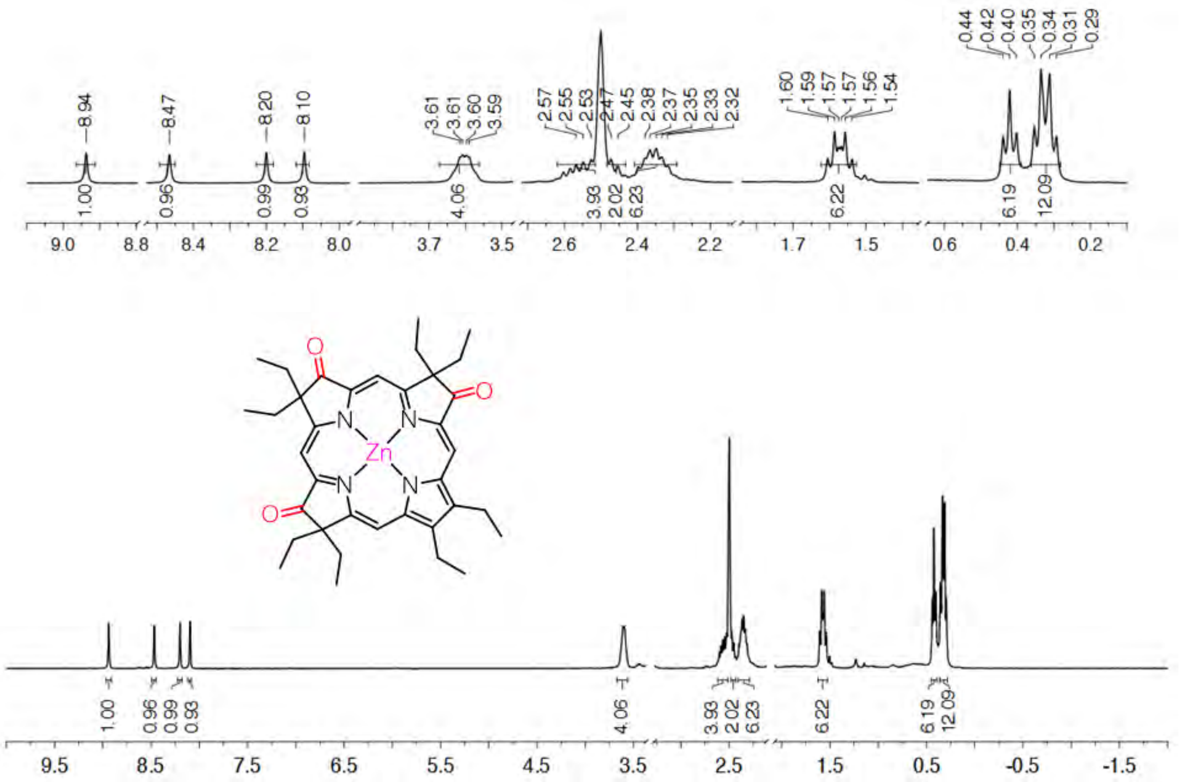

Figure S27. ^{1}H NMR spectrum (400 MHz, DMSO-d$_6$ at 25 °C) of **Zn(II)-8-O^{2,7,12}**.

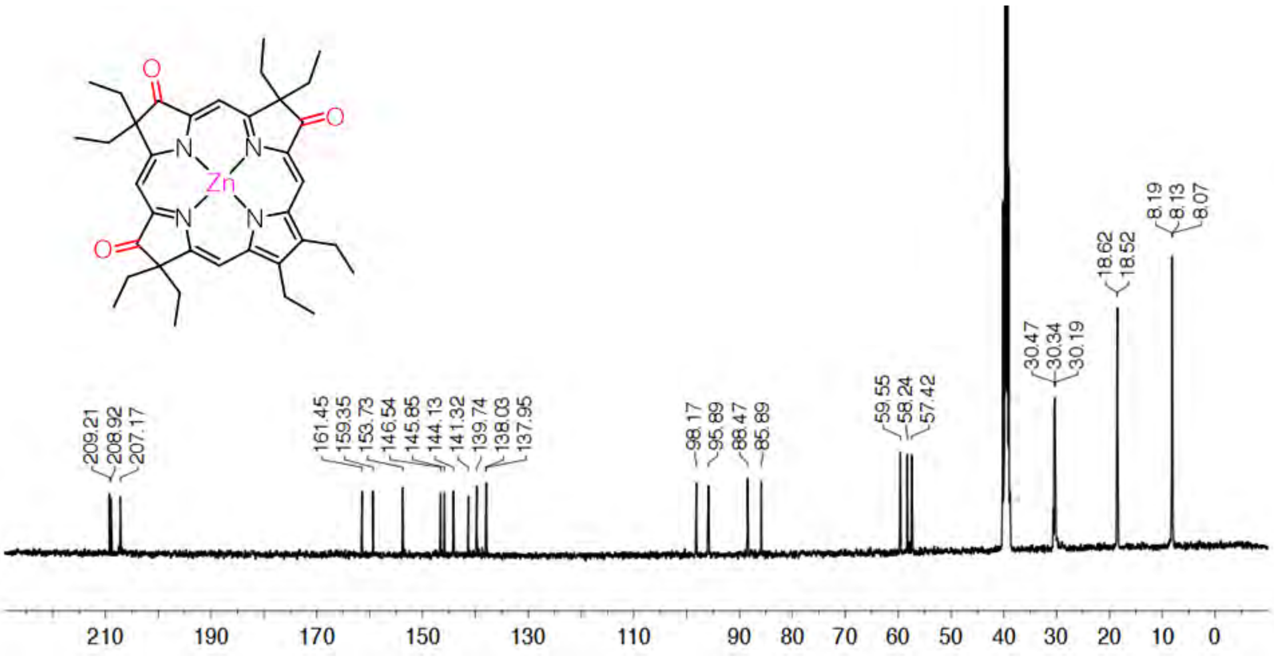

Figure S28. ^{13}C NMR spectrum (101 MHz, DMSO-d$_6$ at 25 °C) of **Zn-8-O^{2,7,12}**.

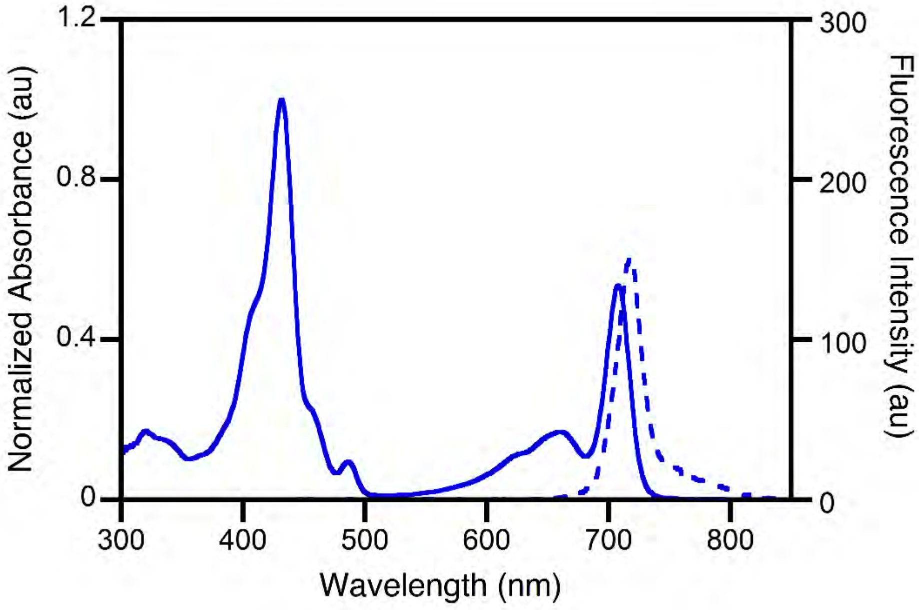

Figure S29. UV-vis (CH_2Cl_2) absorption (solid line) and fluorescence emission (CH_2Cl_2) spectra (broken line) of **Zn-8-O^{2,7,12}**.

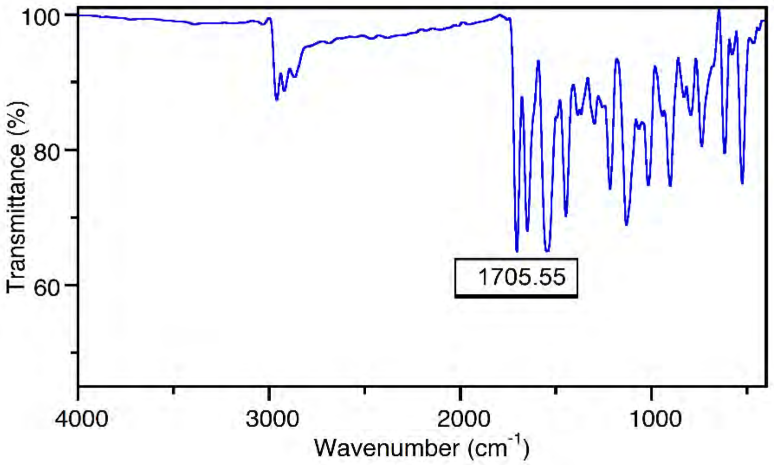

Figure S30. FT-IR Spectrum (neat, ATR) of Zn-**8-O^{2,7,12}**.

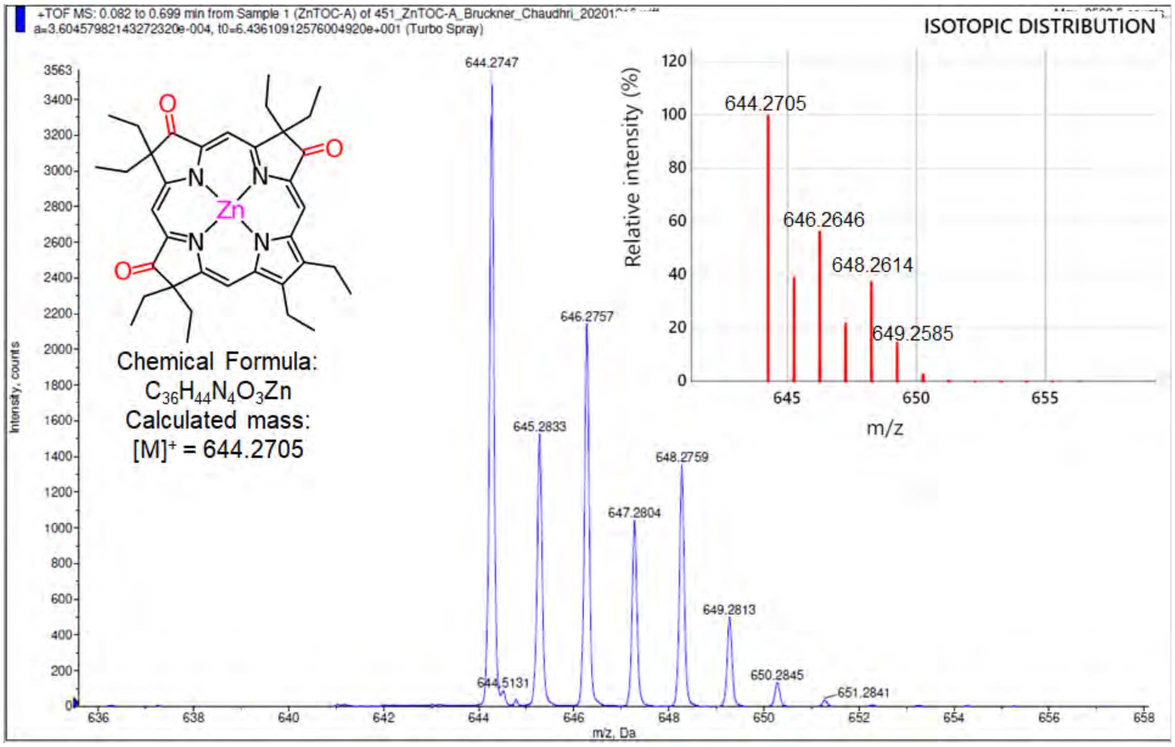

Figure S31. HR-MS Spectrum (ESI+, 100% CH_3CN, TOF) of **Zn-8-O^{2,7,12}**.

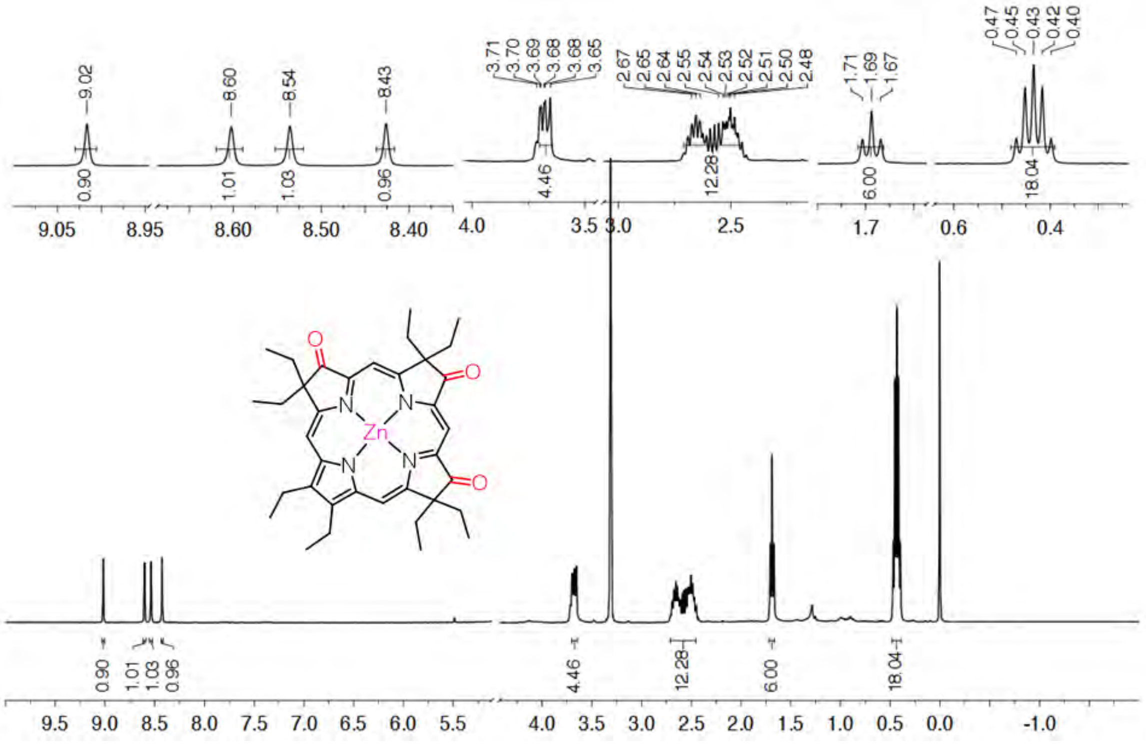

Figure S32. ^{1}H NMR spectrum (400 MHz, methanol-d_4 at 25 °C) of **Zn(II)-8-O^{2,7,18}**.

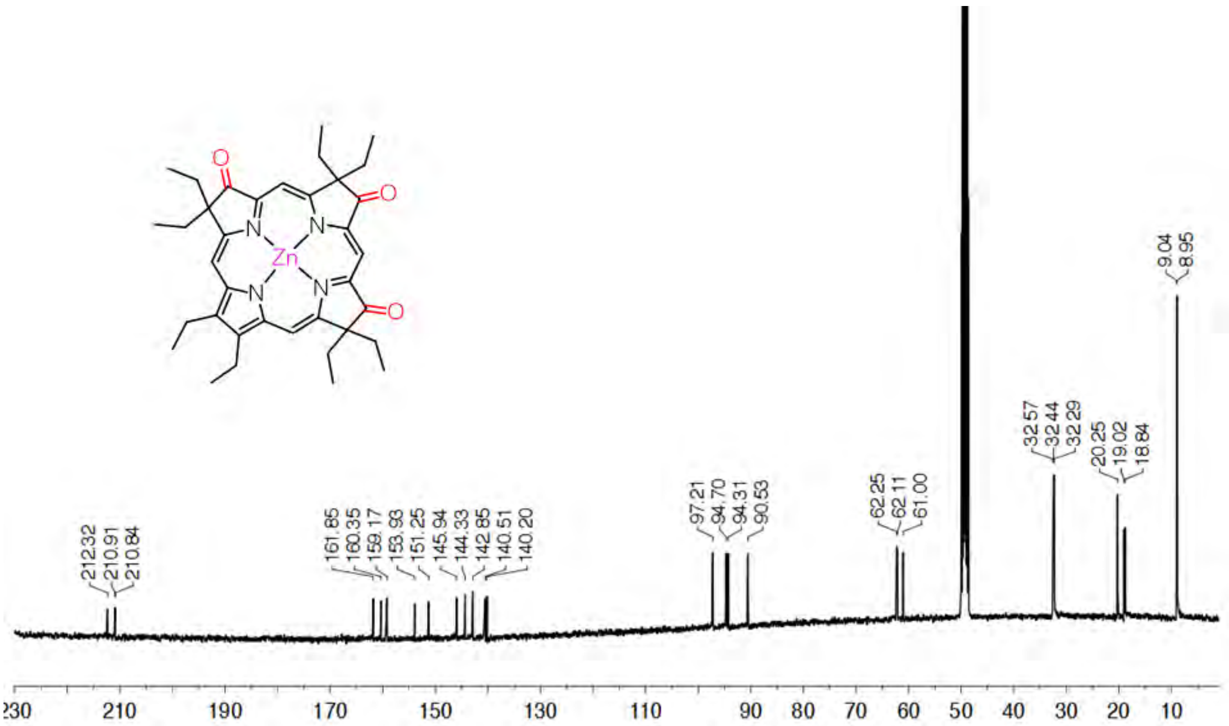

Figure S33. ^{13}C NMR spectrum (101 MHz, methanol-d$_4$ at 25 °C) of **Zn(II)-8-O^{2,7,18}**.

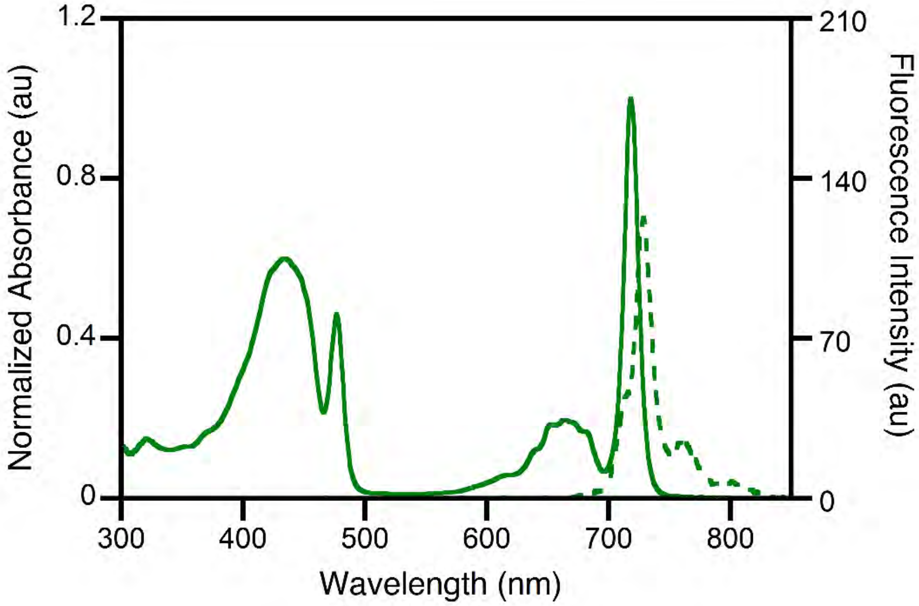

Figure S34. UV-vis (CH_2Cl_2) absorption (solid line) and fluorescence emission (CH_2Cl_2) spectra (broken line) of **Zn(II)-8-O^{2,7,18}**.

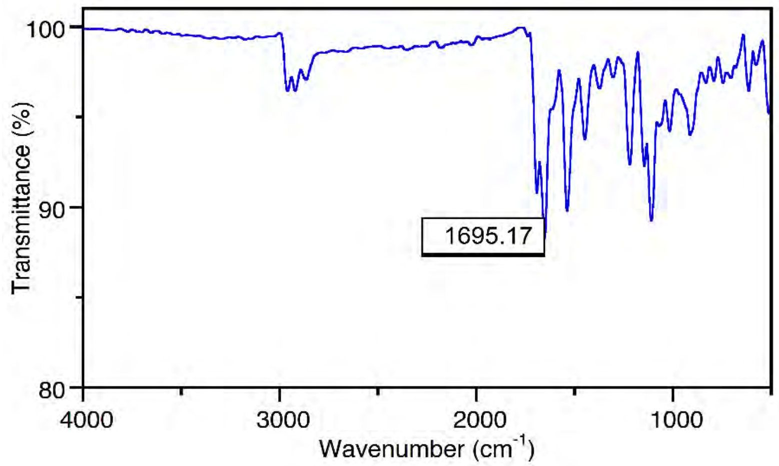

Figure S35. FT-IR Spectrum (neat, ATR) of **Zn(II)-8-O[2,7,18]**.

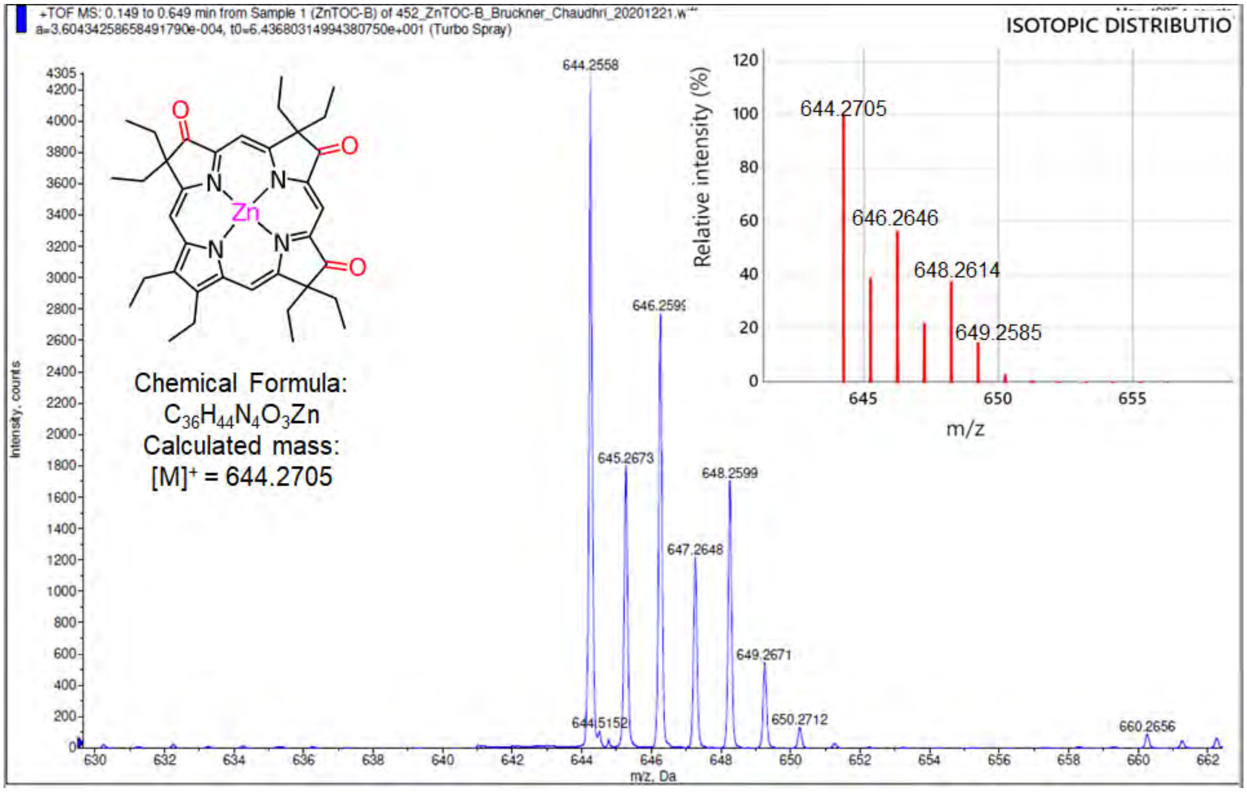

Figure S36. HR-MS Spectrum (ESI+, 100% CH_3CN, TOF) of **Zn(II)-8-O[2,7,18]**.

Halochromic Properties of Trioxopyrrocorphins

For linear graphs, the slope of the graph is taken as the Hill coefficient (η), reflecting the stoichiometry of the event causing the optical change tracked. The graphs returned for the acid titration (TFA in CH_2Cl_2) of the triketones studied here are decidedly non-linear, suggesting the presence of multiple binding events. Thus, it was not possible for us to calculate the Hill coefficient for these reactions. .

The non-linear graphs required a third-order polynominal for best fit ($r^2 > 0.994$).

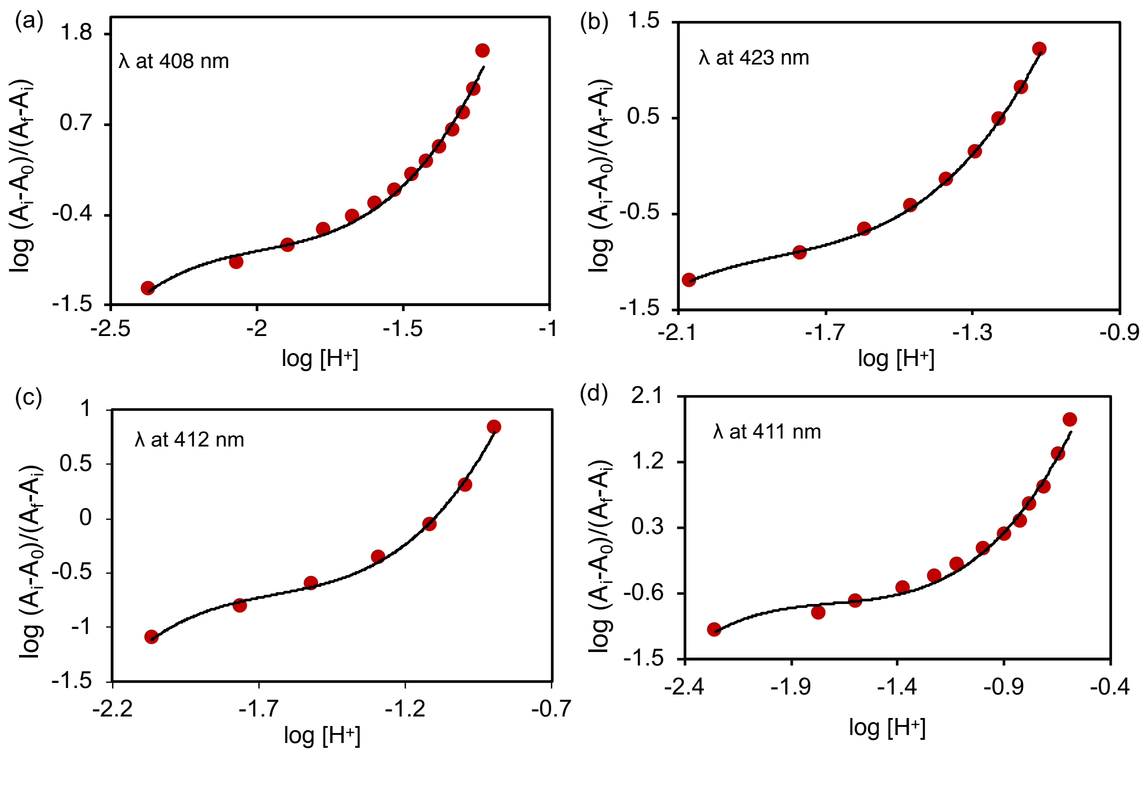

Figure S37. Hill plots for the acid (TFA) titrations of (a) **8-O^{2,7,12}**, (b) **8-O^{2,7,18}**, (c) **8-O^{2,7,13}**, and (d) **8-O^{2,8,18}** in CH_2Cl_2.

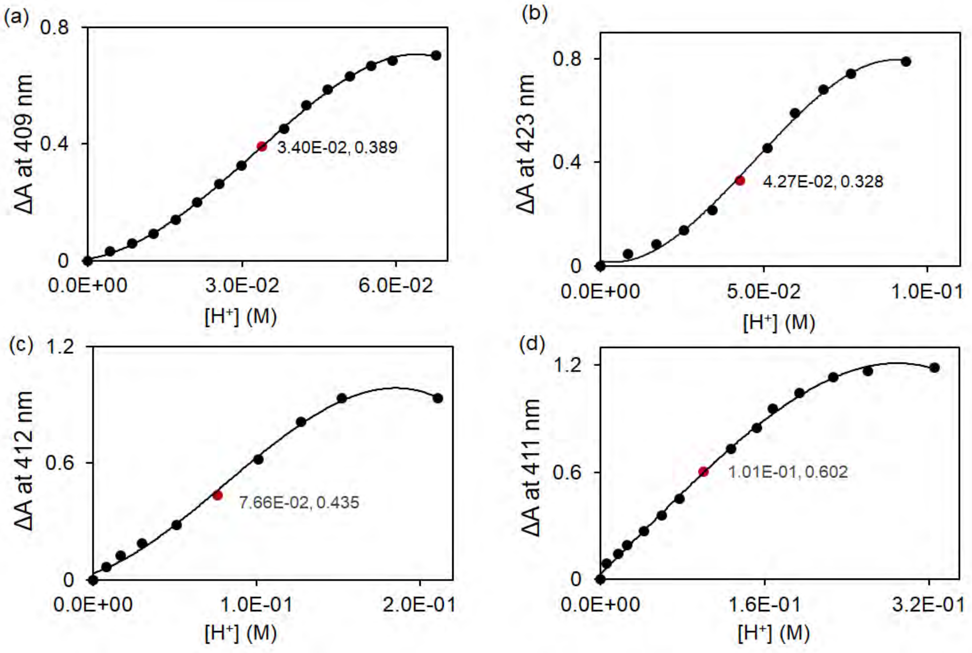

Figure S38. Plot showing total [TFA] to reach 50% protonation for (a) **8-O**$^{\mathbf{2,7,12}}$, (b) for **8-O**$^{\mathbf{2,7,18}}$, (c) **8-O**$^{\mathbf{2,7,13}}$ and (d) **8-O**$^{\mathbf{2,8,18}}$ (in CH_2Cl_2)

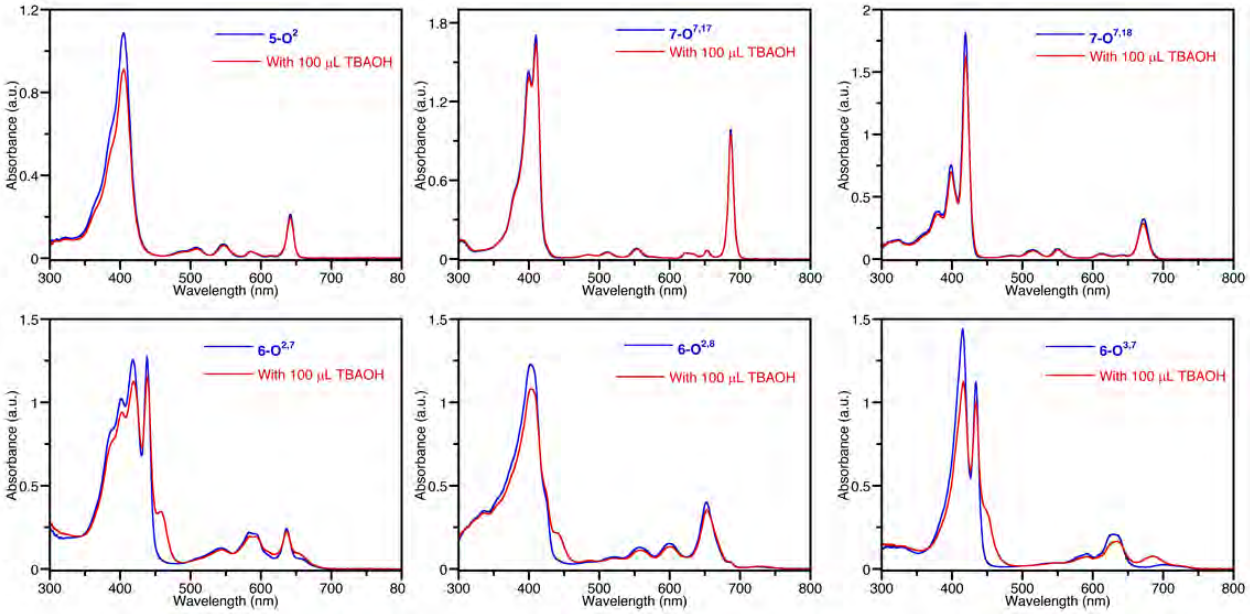

Figure S39. UV-Vis (CH_2Cl_2, blue traces and CH_2Cl_2 + TBAOH, red traces) of the mono-oxo and dioxochlorins indicated. Sufficient TBAOH was added to a sample recorded in red to achieve maximum changes (saturation), with dilution errors < 2%.

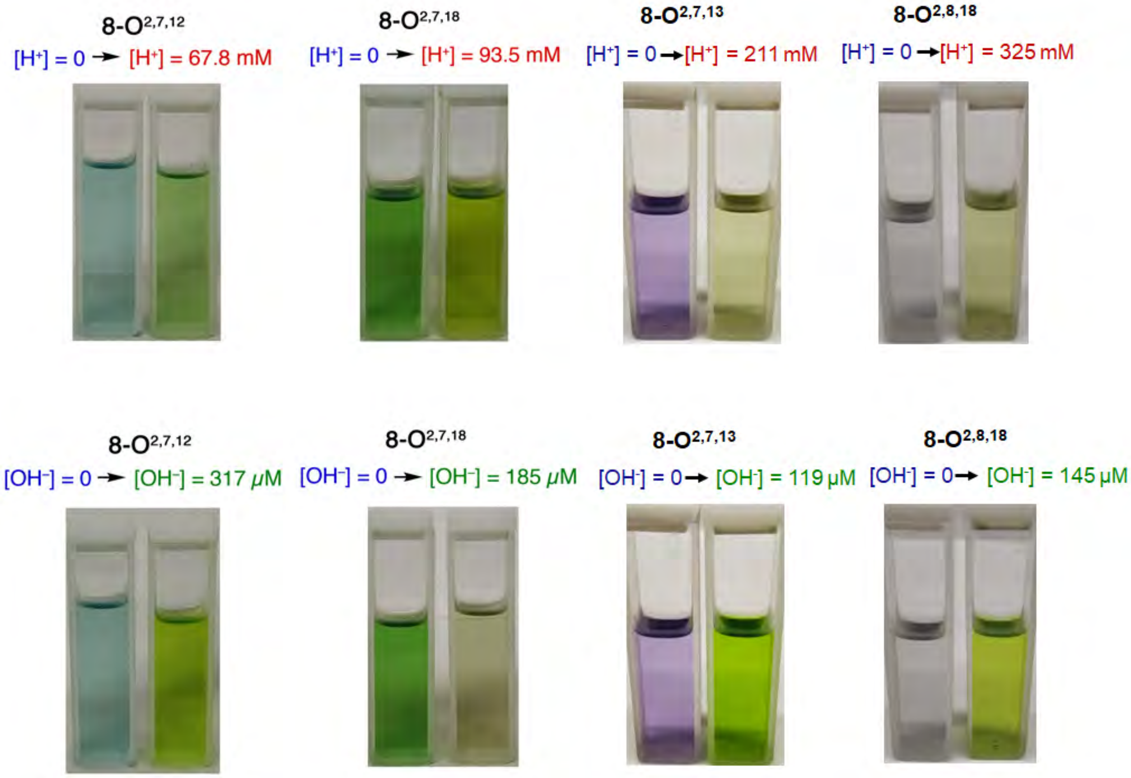

Figure S40. Photographs of the cuvettes containing the solutions indicated, before and after the addition of acid/base.

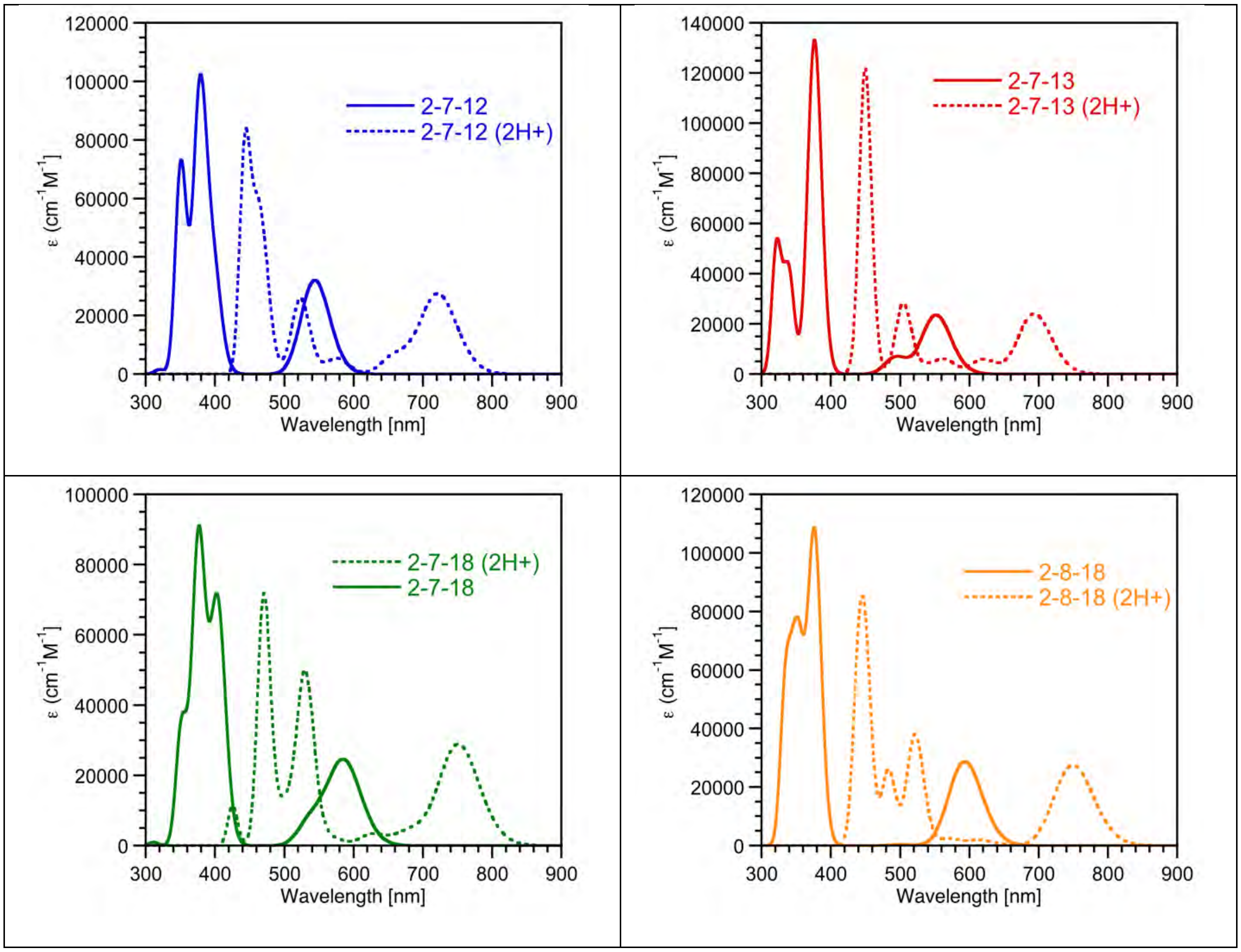

Figure S41. Comparison of the computed UV-vis spectra for the neutral (solid trace) and bisprotonated (broken trace) triketones indicated. The spectra were not shifted to account for systematic errors.

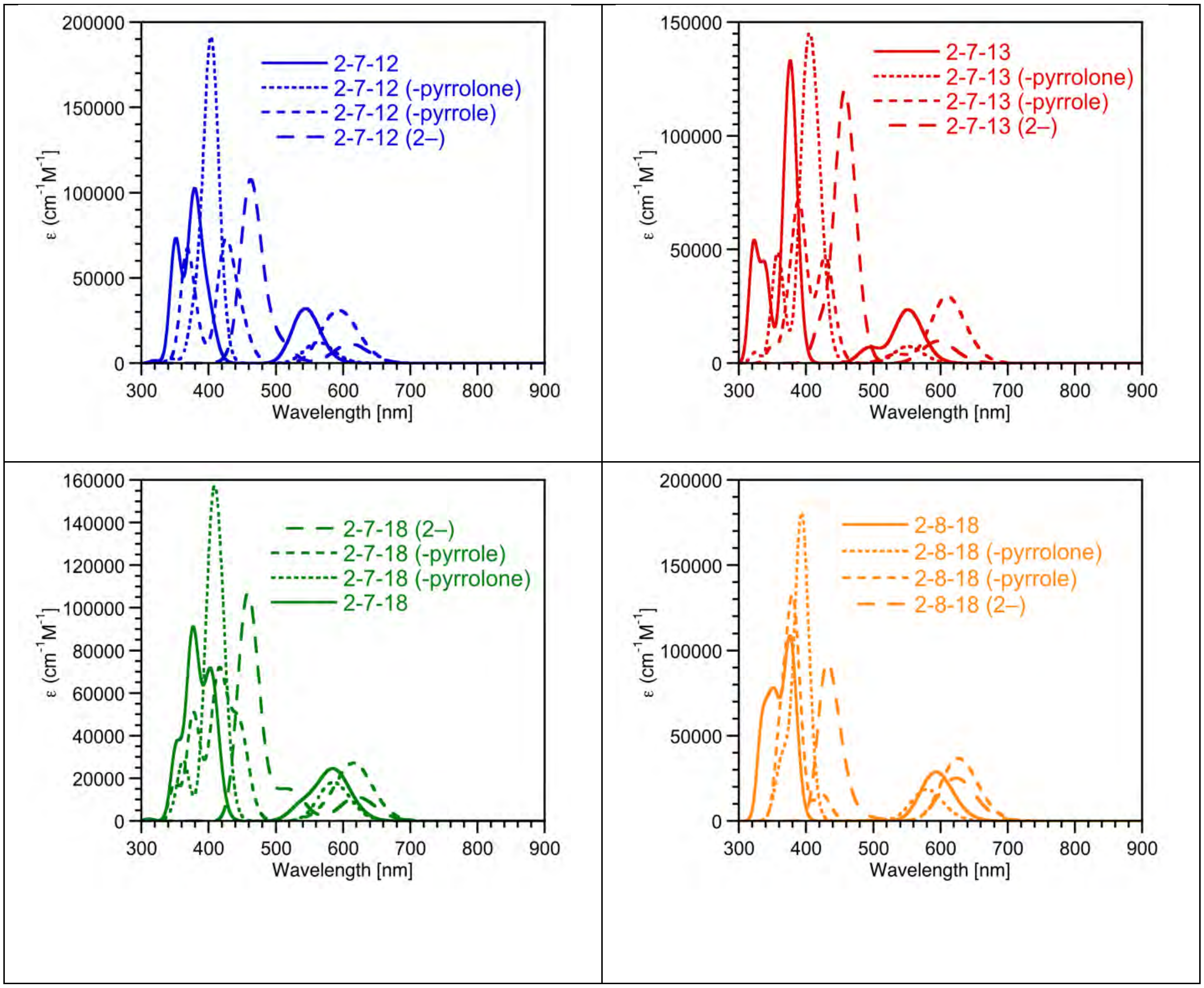

Figure S42. Comparison of the computed UV-vis spectra for the neutral (solid trace) and deprotonated at pyrrole (dotted trace), deprotonated at pyrroline (broken trace), and bis-deprotonated (dashed trace) triketones indicated. The spectra were not shifted to account for systematic errors.

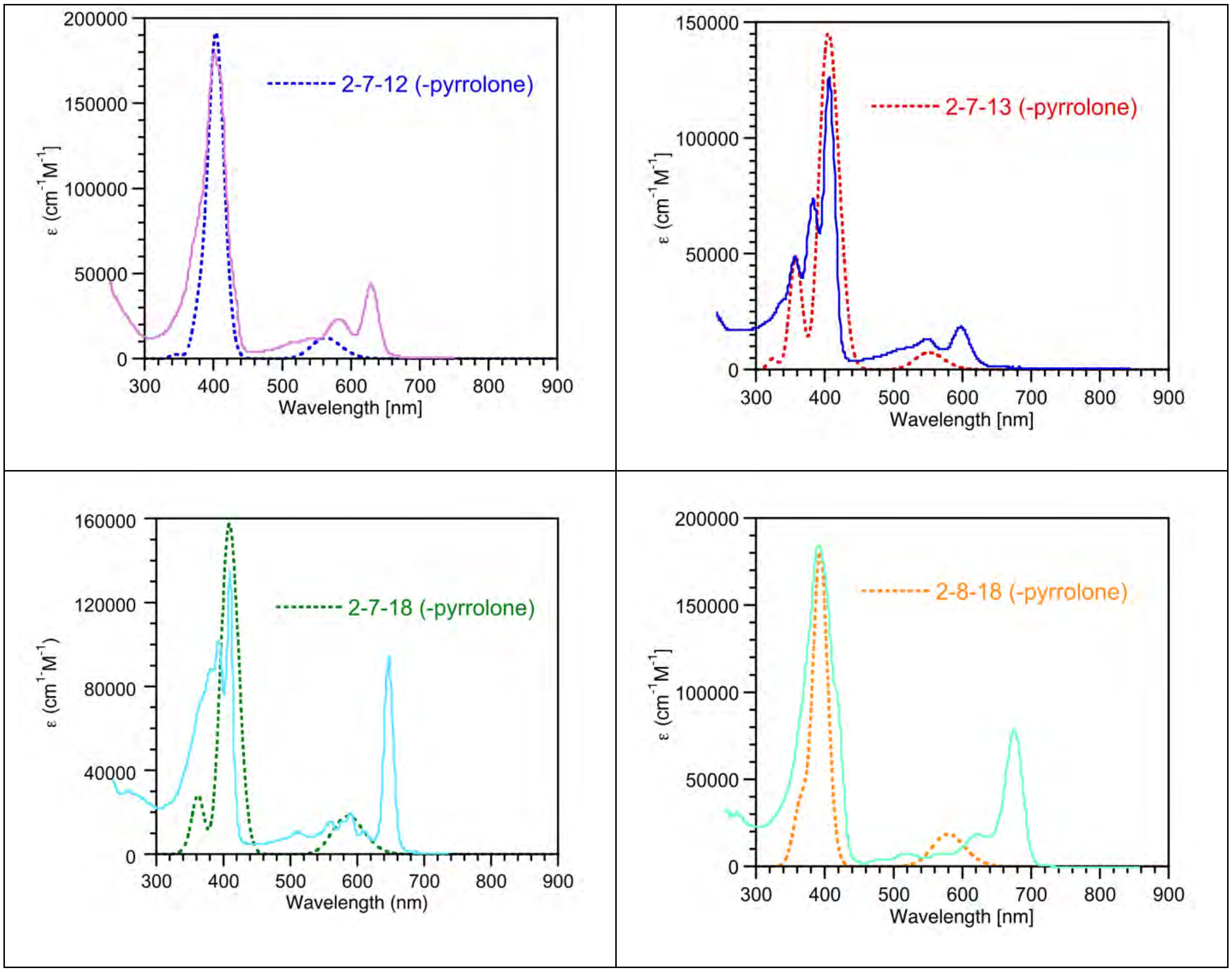

Figure S43. Comparison of the computed UV-vis spectrum (broken line) for the mono-deprotonated (at pyrrolone) species of the triketone indicated with the experimental spectrum at high [OH$^-$] (solid line). The experimental spectra were visually shifted for best fit. ***Note:*** We do not suggest that the species at high [OH$^-$] is indeed mono-deprotonated at the pyrrolone.

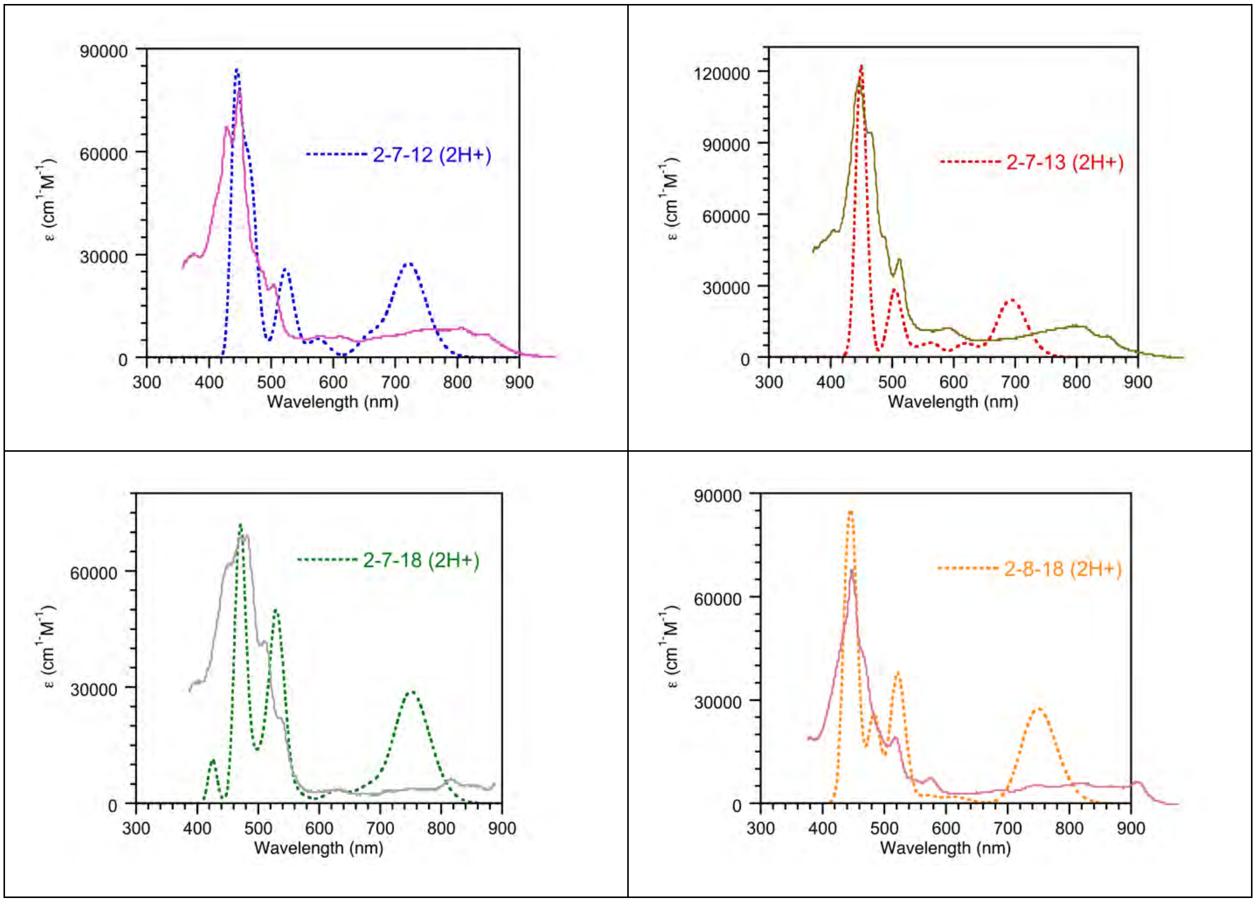

Figure S44. Comparison of the computed UV-vis spectrum (broken line) for the bis-protonated species of the triketone indicated with the experimental spectrum at high [TFA] (solid line). The experimental spectra were visually shifted for best fit. ***Note:*** We do not suggest that the species at high [TFA] is indeed the deprotonated spectrum computed.

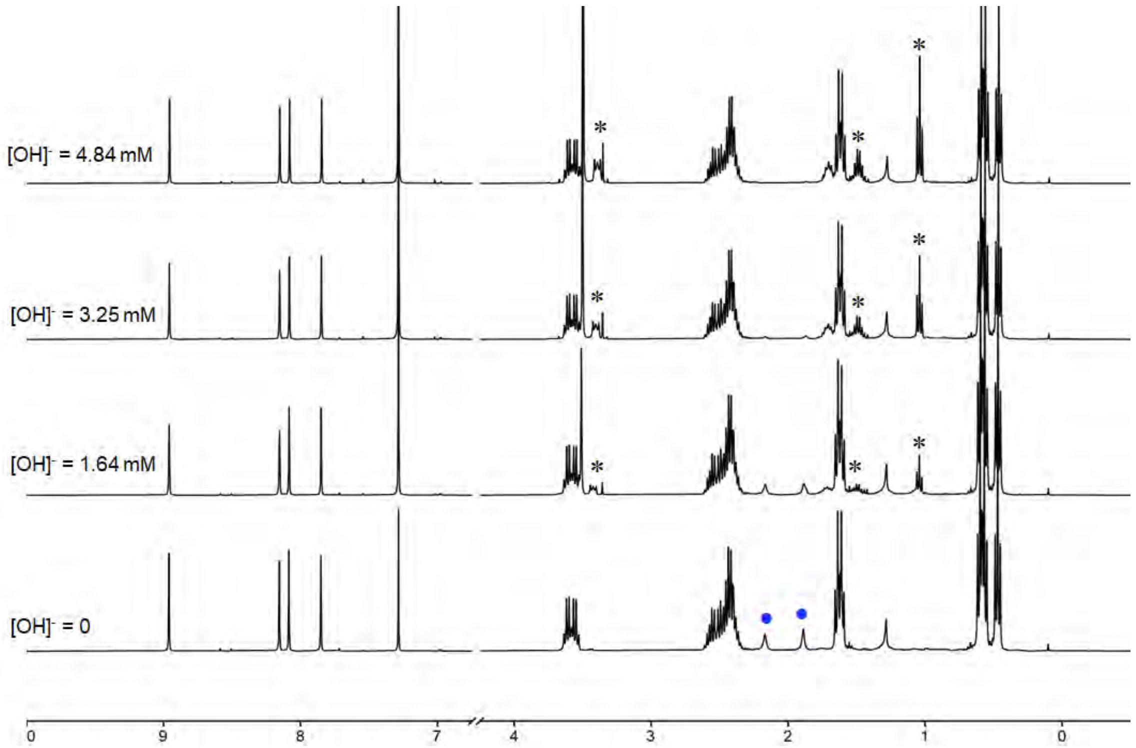

Figure S45. ^{1}H NMR spectral titration of **8-O^{2,7,12}** (0.024 M in $CDCl_3$) with TBAOH (0.166 M in $CDCl_3$). Blue spots indicative of inner core protons. ∗ indicates the signals raised from TBAOH.

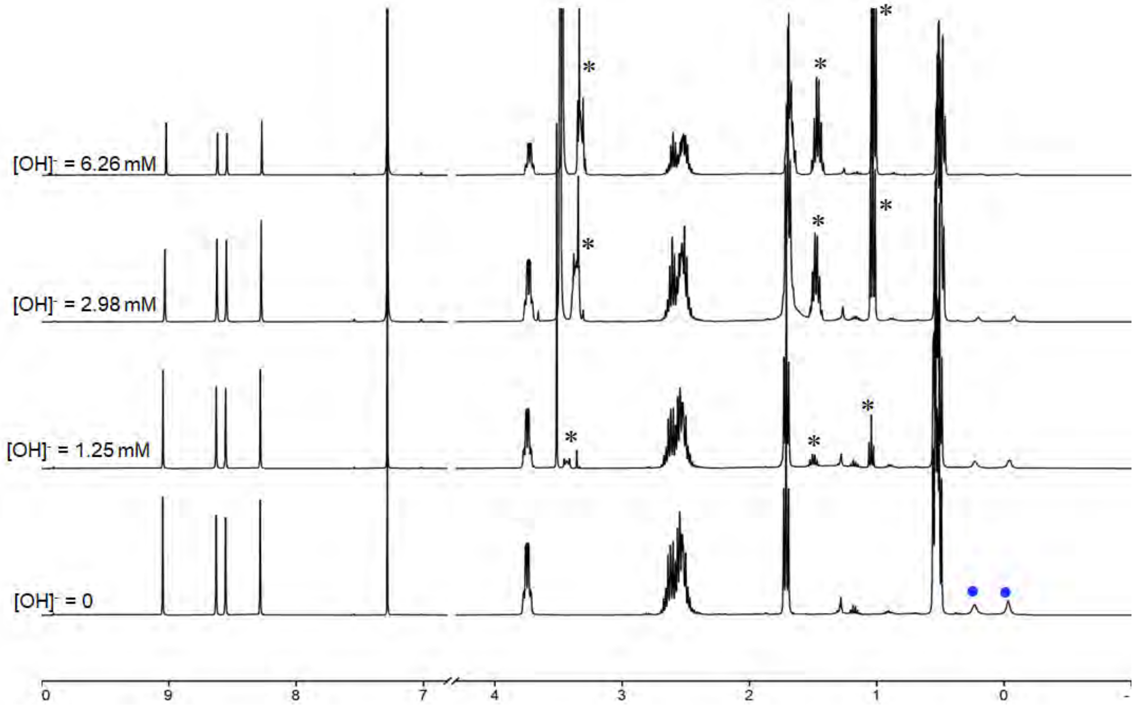

Figure S46. ^{1}H NMR spectral titration of **8-O^{2,7,18}** (0.021 M in $CDCl_3$) with TBAOH (0.091 M in $CDCl_3$). Blue spots indicative of inner core protons. ∗ indicates the signals raised from TBAOH.

X-Ray Crystallography

Data for **8-O^{2,7,18}Zn·pyridine** were collected on a Bruker Quest diffractometer with a fixed chi angle, a Mo Kα wavelength (λ = 0.71073 Å) sealed tube fine focus X-ray tube, single crystal curved graphite incident beam monochromator, and a Photon II area detector. Data for **8-O^{2,7,18}** and **8-O^{2,7,12}Zn·pyridine** were collected on a Bruker Quest diffractometer with kappa geometry, a Cu Kα wavelength (λ = 1.54178 Å) I-μ-S microsource X-ray tube, laterally graded multilayer (Goebel) mirror for monochromatization, and a Photon III C14 area detector. Both instruments were equipped with an Oxford Cryosystems low temperature device and examination and data collection were performed at 150 K. Data were collected, reflections were indexed and processed, and the files scaled and corrected for absorption using APEX3[3] and SADABS[4]. The space groups were assigned using XPREP within the SHELXTL suite of programs[5,6] and solved by direct methods using ShelXS[6] and refined by full matrix least squares against F^2 with all reflections using Shelxl2018[7] and the graphical interface Shelxle[8]. H atoms attached to carbon, boron and nitrogen atoms as well as hydroxyl hydrogens were positioned geometrically and constrained to ride on their parent atoms. C-H bond distances were constrained to 0.95 Å for aromatic and alkene C-H moieties, and to 0.99 and 0.98 Å for aliphatic CH_2 and CH_3 moieties, respectively. N-H bond distances were constrained to 0.88 Å. O-H distances of alcohols were constrained to 0.84 Å. Methyl CH_3 and hydroxyl H atoms were allowed to rotate but not to tip to best fit the experimental electron density. $U_{iso}(H)$ values were set to a multiple of $U_{eq}(C)$ with 1.5 for CH_3 and OH, and 1.2 for C-H, CH_2 and N-H units, respectively.

[3] Bruker (2019). Apex3 v2019.1-0, SAINT V8.40A, Bruker AXS Inc.: Madison (WI), USA.

[4] Krause, L., Herbst-Irmer, R., Sheldrick, G.M. & Stalke, D. (2015). *J. Appl. Cryst.* **48**, 3-10.

[5] SHELXTL suite of programs, Version 6.14, 2000-2003, Bruker Advanced X-ray Solutions, Bruker AXS Inc., Madison, Wisconsin: USA.

[6] Sheldrick, G.M. A short history of SHELX. *Acta Crystallogr A.* **2008**, *64(1)*, 112–122.

[7] a) Sheldrick, G.M. University of Göttingen, Germany, **2018**. b) Sheldrick, G.M. Crystal structure refinement with SHELXL. *Acta Crystallogr Sect C Struct Chem.* **2015**, *71(1)*, 3–8.

[8] Hübschle, C.B., Sheldrick, G.M. & Dittrich, B. ShelXle: a Qt graphical user interface for SHELXL. *J. Appl. Crystallogr.* **2011**, *44(6)*, 1281–1284.

Additional data collection and refinement details, including description of disorder (where present) can be found in the sections below. Complete crystallographic data, in CIF format, have been deposited with the Cambridge Crystallographic Data Centre. CCDC 2064957-2064959 contain the supplementary crystallographic data for this paper. These data can be obtained free of charge from The Cambridge Crystallographic Data Centre via www.ccdc.cam.ac.uk/data_request/cif.

Details to the X-Ray Crystal Structure of 8-$O^{2,7,18}$

The structure is metrically pseudo-tetragonal, but has actual monoclinic Pc symmetry. It is twinned by pseudo-orthorhombic symmetry (β = 90.196(2)°) and was refined as a 2-component twin (twin law 1 0 0, 0 -1 0, 0 0 -1). The twin ratio refined to 0.894(2) to 0.106(2).

Several diethyl methylene moieties were refined as disordered. The units were restrained to have geometries similar as another well-defined diethyl methylene moiety, and U^{ij} components of ADPs were restrained to be similar for atoms closer to each other than 2.0 Å. Subject to these conditions the occupancy ratios refined to 0.850(16) to 0.150(16) for the moiety of C13A, to 0.665(15) to 0.335(16) for that of C8B, and to 0.797(10) to 0.203(10) for that of C13B.

Two small voids of around 68 $Å^3$ (2% of the cell volume) between molecules were occupied by disordered semi-liquid solvate molecules. No substantial electron density peaks were found in the solvent accessible voids (less than 0.55 electron per $Å^3$) and the residual electron density peaks are not arranged in an interpretable pattern. The cif and fcf files were thus corrected for using reverse Fourier transform methods using the SQUEEZE routine (P. van der Sluis & A.L. Spek (1990). Acta Cryst. A46, 194-201) as implemented in the program Platon. The resultant files were used in the further refinement. (The FAB file with details of the Squeeze results is appended to the cif file). The Squeeze procedure corrected for two times 13 electrons within the

solvent accessible voids, indicating disordered and/or partially occupied methanol (one of the crystallization solvents).

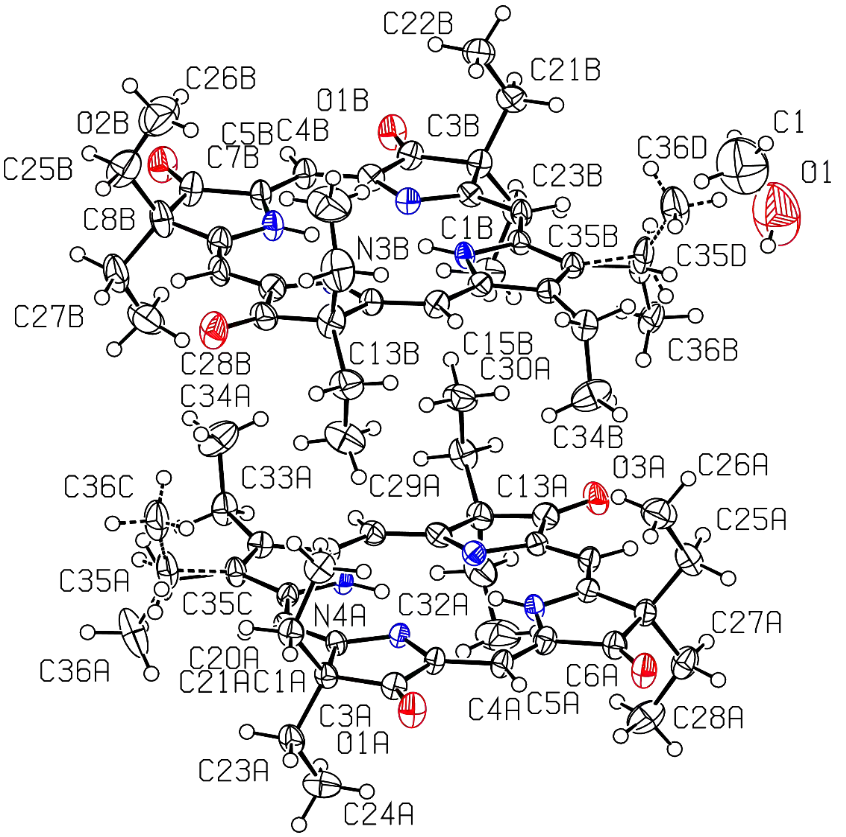

Figure S47. Probability ellipsoid representation of triketone **8-O[2,7,18]** oblique view, displacement parameters at the 50% probability level.

Table S1. Experimental Details to the X-ray Diffraction Analyses of **8-O[2,7,18]**.

	**8-O[2,7,18]**
**Crystal data**	
Chemical formula	$C_{36}H_{46}N_4O_3 \cdot 0.323(CH_4O)$
M_r	593.12
Crystal system, space group	Monoclinic, *Pc*
Temperature (K)	150
a, *b*, *c* (Å)	12.2252 (4), 12.1333 (4), 22.9886 (8)
β (°)	90.0171 (16)
V (Å^3)	3409.9 (2)
Z	4

$F(000)$	1279.3
D_x (Mg m^{-3})	1.155
Radiation type	Cu $K\alpha$
No. of reflections for cell measurement	9928
θ range (°) for cell measurement	3.6–79.0
μ (mm^{-1})	0.59
Crystal shape	Flake
Colour	Green
Crystal size (mm)	0.14 × 0.12 × 0.03
**Data collection**	
Diffractometer	Bruker AXS D8 Quest diffractometer with PhotonIII_C14 charge-integrating and photon counting pixel array detector
Radiation source	I-μ-S microsource X-ray tube
Monochromator	Laterally graded multilayer (Goebel) mirror
Detector resolution (pixels mm^{-1})	7.4074
Scan method	ω and phi scans
Absorption correction	Multi-scan, *SADABS* 2016/2: Krause, L., Herbst-Irmer, R., Sheldrick G.M. & Stalke D., *J. Appl. Cryst.* 48 (2015) 3-10.
T_{min}, T_{max}	0.683, 0.754
No. of measured, independent and observed [$I > 2\sigma(I)$] reflections	36871, 13171, 12382
R_{int}	0.043
θ values (°)	θ_{max} = 79.9, θ_{min} = 1.9
$(\sin \theta/\lambda)_{max}$ (Å^{-1})	0.639
Range of h, k, l	h = -15→13, k = -15→13, l = -28→29
**Refinement**	
Refinement on	F^2
$R[F^2 > 2\sigma(F^2)]$, $wR(F^2)$, S	0.046, 0.120, 1.06
No. of reflections	13171
No. of parameters	851
No. of restraints	99
H-atom treatment	H-atom parameters constrained
Weighting scheme	$w = 1/[\sigma^2(F_o^2) + (0.0686P)^2 + 0.1612P]$ where $P = (F_o^2 + 2F_c^2)/3$
$(\Delta/\sigma)_{max}$	< 0.001
$\Delta\rho_{max}$, $\Delta\rho_{min}$ (e Å^{-3})	0.42, -0.20
Absolute structure	Flack x determined using 5161 quotients [(I+)-(I-)]/[(I+)+(I-)] (Parsons, Flack and Wagner, Acta Cryst. B69 (2013) 249-259).
Absolute structure parameter	0.46 (10)
CCDC	2064958

Details to the X-Ray Crystal Structure of 8-$O^{2,7,18}$Zn·pyridine

Two crystallographically independent molecules are present in the lattice. The two molecules are related to each other by a pseudo B-centering lattice translation. Exact translational symmetry is broken by modulation of one ethyl group (C33, C34), which is oriented differently in the two molecules. Identical orientations of the ethyl groups is mutually incompatible due to a close contact. Ethyl groups are disordered over two orientations with the major moiety of each molecule related by B-centering to the minor moiety of the other molecule.

Exact translational symmetry is also broken by disorder of one Et_2C moiety in molecule B. Disorder is absent in molecule A.

Major and minor moieties of each disordered segment were restrained to have similar geometries. ADPs of atoms C17A and C17C, and C17B and C17D were constrained to be each identical. U^{ij} components of ADPs for disordered atoms closer to each other than 2.0 Å were restrained to be similar. Subject to these conditions the joined occupancy ratio for the ethyl disorder (C17, C33, C34) refined to 0.824(3) to 0.176(3). The disorder ration for the Et_2C moiety of molecule B refined to 0.509(4) to 0.491(4).

Table S2. Experimental Details to the X-ray Diffraction Analyses of **8-O^{2,7,18}Zn·pyridine**.

	**8-O^{2,7,18}Zn-pyridine**
**Crystal data**	
Chemical formula	$C_{41}H_{49}N_5O_3Zn$
M_r	725.22
Crystal system, space group	Triclinic, $P\bar{1}$
Temperature (K)	150
a, *b*, *c* (Å)	12.0722 (6), 18.1055 (9), 19.2232 (10)
α, β, γ (°)	67.041 (2), 88.923 (2), 78.976 (2)
V (Å^3)	3790.2 (3)
Z	4
F(000)	1536
D_x (Mg m^{-3})	1.271
Radiation type	Mo *K*α
No. of reflections for cell measurement	9309
θ range (°) for cell measurement	2.5–33.0
μ (mm^{-1})	0.69
Crystal shape	Block
Colour	Green
Crystal size (mm)	0.41 × 0.23 × 0.17
**Data collection**	
Diffractometer	Bruker AXS D8 Quest diffractometer with PhotonII charge-integrating pixel array detector (CPAD)
Radiation source	fine focus sealed tube X-ray source
Monochromator	Triumph curved graphite crystal
Detector resolution (pixels mm^{-1})	7.4074
Scan method	ω and phi scans
Absorption correction	Multi-scan, *SADABS* 2016/2: Krause, L., Herbst-Irmer, R., Sheldrick G.M. & Stalke D., *J. Appl. Cryst.* 48 (2015) 3-10.
T_{min}, T_{max}	0.694, 0.747
No. of measured, independent and observed [$I > 2\sigma(I)$] reflections	277906, 29002, 22766
R_{int}	0.045
θ values (°)	θ_{max} = 33.2, θ_{min} = 2.0
(sin θ/λ)$_{max}$ (Å^{-1})	0.771
Range of *h*, *k*, *l*	*h* = -18→18, *k* = -27→27, *l* = -29→29

**Refinement**	
Refinement on	F^2
$R[F^2 > 2\sigma(F^2)]$, $wR(F^2)$, S	0.058, 0.168, 1.08
No. of reflections	29002
No. of parameters	1029
No. of restraints	284
H-atom treatment	H-atom parameters constrained
Weighting scheme	$w = 1/[\sigma^2(F_o^2) + (0.0597P)^2 + 4.6782P]$ where $P = (F_o^2 + 2F_c^2)/3$
$(\Delta/\sigma)_{max}$	0.001
$\Delta\rho_{max}$, $\Delta\rho_{min}$ (e Å^{-3})	1.33, -1.40
Extinction method	*SHELXL2018*/3 (Sheldrick 2018), $Fc^*=kFc[1+0.001xFc^2\lambda^3/\sin(2\theta)]^{-1/4}$
Extinction coefficient	0.0031 (5)
CCDC	2064959

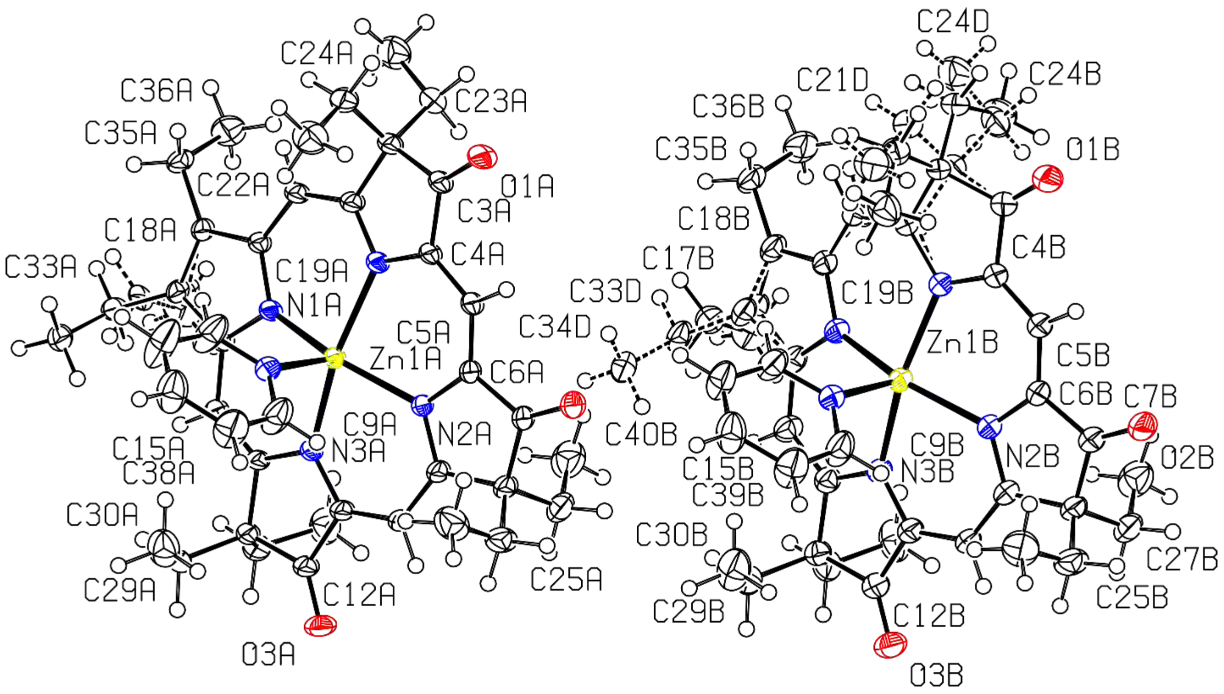

Figure S48. Probability ellipsoid representation of **8-O^{2,7,18}Zn·pyridine** oblique views. Anisotropically refined displacement parameters shown at the 50% probability level.

Details to the X-Ray Crystal Structure of 8-O^{2,7,12}Zn·pyridine

A solvate area is occupied by either methanol or water. The major methanol fraction is internally hydrogen bonded to form tetramers. The minor water fraction, consists of two times three water molecules, in part H-bonded to one of the keto oxygen atoms and among themselves. Methanol C-O bond distances were restrained to be similar in length.

Two water O atoms, O5B and O6B, were restrained to be at least 2.80(2) Å apart. Water H atom positions were initially refined while a damping factor was applied and O-H and H···H distances were restrained to 0.84(2) and 1.36(2) Å, respectively. Some water H atom positions were further restrained based on hydrogen bonding considerations and an anti-bumping restraint was applied to avoid close H···H contacts. In the final refinement cycles the damping factor was removed and water H atoms were constrained to ride on their carrying oxygen atom. U^{ij} components of ADPs for disordered atoms closer to each other than 2.0 Å were restrained to be similar. Subject to these conditions the occupancy ratio refined to 0.781(6) to 0.219(6).

Table S3. Experimental Details to the X-ray Diffraction Analyses of **8-O^{2,7,12}Zn·pyridine**.

	**8-O^{2,7,12}Zn-pyridine**
**Crystal data**	
Chemical formula	$C_{41}H_{49}N_5O_3Zn·1.562(CH_4O)·0.657(H_2O)$
M_r	787.09
Crystal system, space group	Triclinic, $P\bar{1}$
Temperature (K)	150
a, *b*, *c* (Å)	10.7675 (10), 11.1454 (13), 17.315 (2)
α, β, γ (°)	89.825 (5), 87.094 (6), 82.139 (5)
V (Å^3)	2055.7 (4)
Z	2
F(000)	837.4
D_x (Mg m^{-3})	1.272

Radiation type	Cu *K*α
No. of reflections for cell measurement	7884
θ range (°) for cell measurement	4.7–80.0
μ (mm^{-1})	1.23
Crystal shape	Block
Colour	Black
Crystal size (mm)	0.19 × 0.17 × 0.11
**Data collection**	
Diffractometer	Bruker AXS D8 Quest diffractometer with PhotonIII_C14 charge-integrating and photon counting pixel array detector
Radiation source	I-μ-S microsource X-ray tube
Monochromator	Laterally graded multilayer (Goebel) mirror
Detector resolution (pixels mm^{-1})	7.4074
Scan method	ω and phi scans
Absorption correction	Multi-scan, *SADABS* 2016/2: Krause, L., Herbst-Irmer, R., Sheldrick G.M. & Stalke D., J. Appl. Cryst. 48 (2015) 3-10
T_{min}, T_{max}	0.608, 0.754
No. of measured, independent and observed [$I > 2\sigma(I)$] reflections	19177, 8185, 6640
R_{int}	0.050
θ values (°)	θ_{max} = 80.2, θ_{min} = 4.7
$(\sin \theta/\lambda)_{max}$ ($Å^{-1}$)	0.639
Range of *h*, *k*, *l*	$h = -12\rightarrow13$, $k = -14\rightarrow13$, $l = -20\rightarrow22$
**Refinement**	
Refinement on	F^2
$R[F^2 > 2\sigma(F^2)]$, $wR(F^2)$, S	0.053, 0.143, 1.05
No. of reflections	8185
No. of parameters	527
No. of restraints	60
H-atom treatment	H-atom parameters constrained
Weighting scheme	$w = 1/[\sigma^2(F_o^2) + (0.0569P)^2 + 2.3554P]$ where $P = (F_o^2 + 2F_c^2)/3$
$(\Delta/\sigma)_{max}$	0.001
$\Delta\rho_{max}$, $\Delta\rho_{min}$ (e $Å^{-3}$)	0.88, -0.74
CCDC Number	2064957

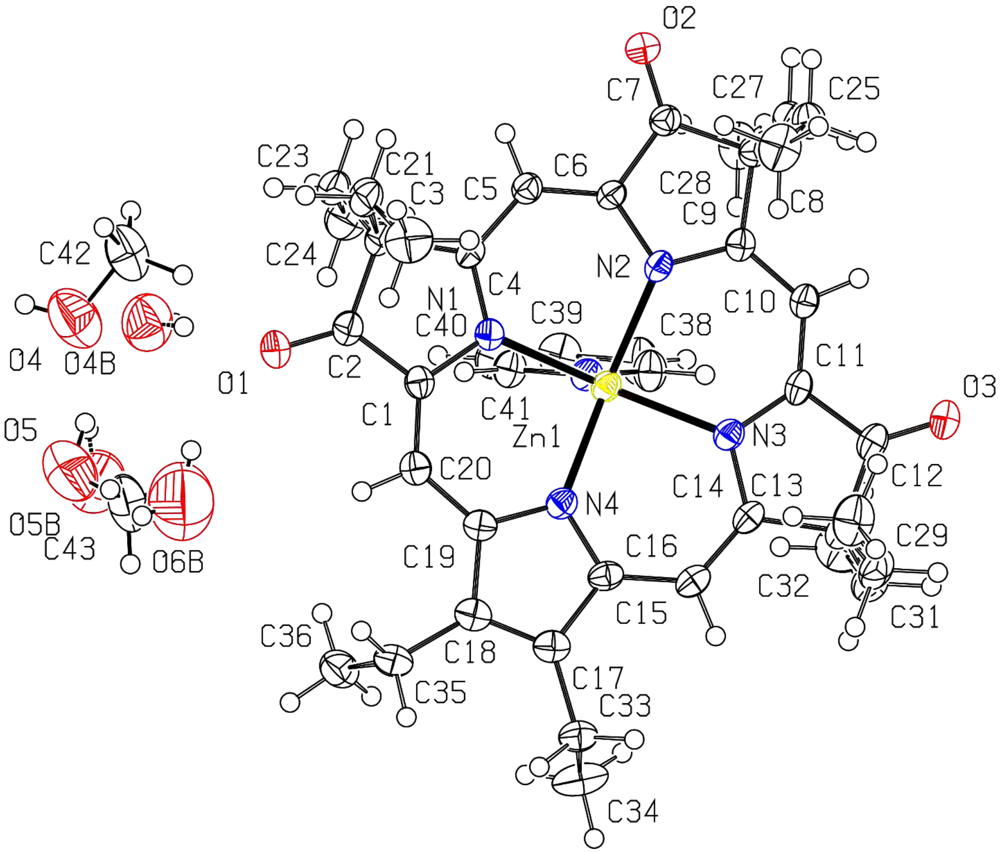

Figure S49. Probability ellipsoid representation of **8-O^{2,7,12}Zn·pyridine** top view. Anisotropically refined displacement parameters shown at the 50% probability level

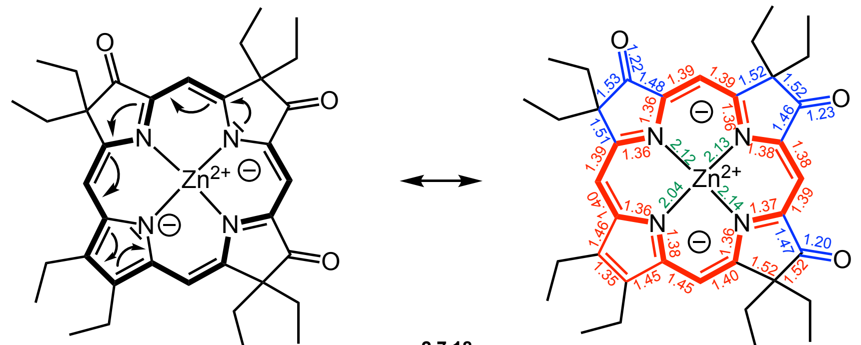

8-O^{2,7,18}Zn

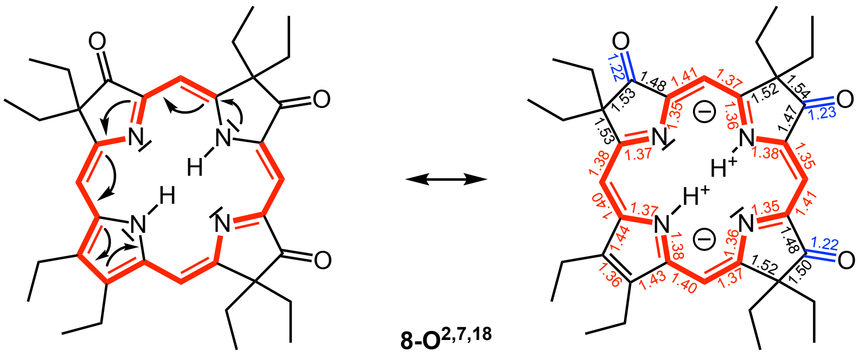

8-O^{2,7,18}

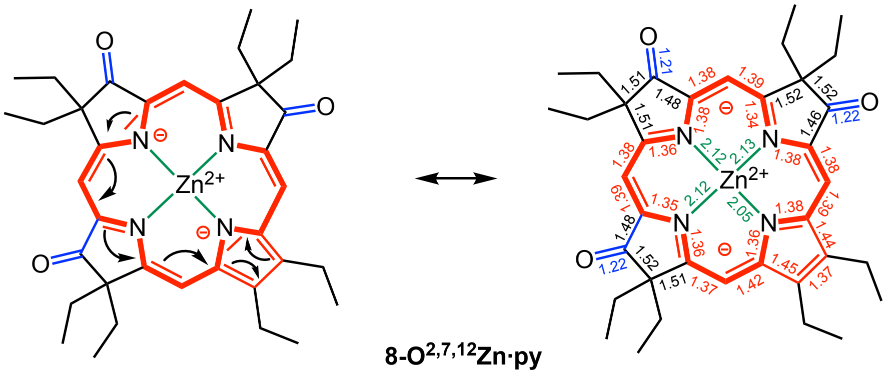

8-O^{2,7,12}Zn·py

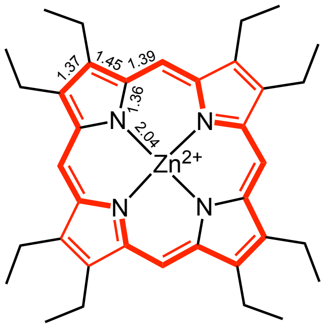

for comparison, bond lengths (in Å) in [octaethylporphyrinato]Zn(II) (CCDC code ALOKOB):

Figure S50. Bond length analysis of the structures indicated, and resonance structures deduced shown in red. Axial pyridine group and all ethyl substituents omitted for clarity. Coordination bonds: green; isolated double bonds: red; single bonds (> 1.44 Å): blue; conjugated double bonds (between 1.35 and 1.45 Å): red.

Computational Methods

The trioxopyrrocorphin isomers in the neutral, singly (mono-) and doubly (di-anionic) deprotonated, and doubly (di-cationic) protonated states were geometry optimized with the BHandHLYP approximate density functional[9] and a dev2-SVP basis set.[10] The absence of negative or imaginary frequencies in a harmonic vibrational analysis was used to confirm that each of the obtained structures resided in a local minimum on the potential energy surface.

Our primary objective for the computations was to provide insight into the regioisomeric-dependent aromatic nature of the trioxopyrrocorphins. We have found through calculations on related porphotrilactone structures, which will be published elsewhere, that the best agreement between theory and experiment for the magnetic criterion of aromaticity is obtained by performing geometry optimizations and nuclear magnetic resonance (NMR) computations with a density functional, like BHandHLYP, that contains 50% exact (Hartree-Fock) exchange. This conclusion is consistent with prior work[11] and motivated the choice over the level of theory for our studies.

NMR computations were performed on the four neutral trioxopyrrocorphin isomers at the BHandHLYP/def2-TZVP[9,10] level of theory. The difference in the average ^{1}H chemical shifts for the inner N-H and the outer *meso*-C-H protons ($\delta\Delta_{NH,meso}$) was computed as a measure of the diatropic ring current that could be directly compared to experiment. At the chosen level of theory, the comparison was found to be excellent, reproducing experimental $\delta\Delta_{NH,meso}$ values within 0.4 ppm. To build on this result, the Iso-chemical shielding surface approach (ICSS)[12] implemented in MultiWFN[13] was employed to visualize the spatial distribution of the ZZ

---

[9] Becke, A. D., A new mixing of Hartree–Fock and local density-functional theories. *J. Chem. Phys.* **1993,** *98*, 1372–1377.

[10] Weigend, F.; Ahlrichs, R., Balanced basis sets of split valence, triple zeta valence and quadruple zeta valence quality for H to Rn: Design and assessment of accuracy. *Phys. Chem. Chem. Phys.* **2005,** *7*, 3297–3305.

[11] Wannere, C. S.; Sattelmeyer, K. W.; Schaefer III, H. F.; Schleyer, P. v. R., Aromaticity: The Alternating C—C Bond Length Structures of [14]-,[18]-, and [22] Annulene. *Angew. Chem., Int. Ed.* **2004,** *43*, 4200–4206.

[12] (a) Klod, S.; Kleinpeter, E., *J. Chem. Soc., Perkin Transs 2* **2001,** 1893–1898. (b) Liu, Z.; Lu, T.; Chen, Q., *Carbon* **2020,** *165*, 468–475.

[13] Lu, T.; Chen, F., *J. Comp. Chem.* **2012,** *33*, 580–592.

component of the magnetic shielding tensor 1.0 Å above the macrocyclic plane. In the ICSS method, which is a three-dimensional generalization of the nucleus independent chemical shift (NICS) technique,[14] a cube of evenly spaced probe or phantom atoms is placed around the molecule of interest, and the magnetic shielding at these points is computed. A cube of 132,440 probe atoms was constructed using the default settings for a "medium" quality grid in MultiWFN for each trioxopyrrocorphin.

The NICS(0) and NICS(1) values are based on the isotropic magnetic shielding value and the NICS metrics follow the same trend as the difference in NH-meso chemical shifts (Table S4)

Table S4. Calculated NICS(0) and NICS(1) values for the trioxopyrrocorphins.

Trioxopyrrocorphins	δΔ (ppm)	NICS(0)	NICS(0)zz	NICS(1)	NICS(1)zz
**8-O^{2,7,13}**	4.18	-4.75	-7.32	-4.76	-11.79
**8-O^{2,7,12}**	5.99	-5.95	-11.12	-5.85	-15.31
**8-O^{2,8,18}**	6.01	-6.03	-11.38	-5.93	-15.37
**8-O^{2,7,18}**	8.48	-7.74	-16.68	-7.46	-20.20

Note: the molecules in the calculation were oriented in the yz plane, so we are reporting the xx component of the magnetic shielding tensor. We are using the "zz" label because the result is invariant to rotation, and either way, we are providing the tensor component perpendicular to the macrocyclic plane.

The UV-vis absorption spectra for each trioxopyrrocorphin in the neutral, singly or doubly de-protonated, and singly protonated states were computed with the hybrid PBE0 density functional[15] and a 6-31+G(d) basis set.[16] We have shown previously that this level of theory well reproduces the optical spectra of porphyrinoids bearing β-oxo functionalities.[17]

[14] Chen, Z.; Wannere, C. S.; Corminboeuf, C.; Puchta, R.; Schleyer, P. V. R., *Chem. Rev.* **2005,** *105*, 3842–3888.

[15] Adamo, C.; Barone, V., *J. Chem. Phys.* **1999,** *110*, 6158–6170.

All computations were performed with an ultrafine integration grid and tight self-consistent field (SCF) convergence as implemented in Gaussian 09, Rev. D.01.[18]

The coordinates of all computed structures are available as .pdf files in the ESI.

---

[16] (a) Hehre, W. J.; Ditchfield, R.; Pople, J. A., *J. Chem. Phys.* **1972,** *56*, 2257–2261. (b) Hariharan, P. C.; Pople, J. A., *Theor. Chim. Acta* **1973,** *28*, 213–222. (c) Clark, T.; Chandrasekhar, J.; Spitznagel, G. W.; Schleyer, P. V. R., *J. Comp. Chem.* **1983,** *4*, 294–301.

[17] Guberman-Pfeffer, M. J.; Lalisse, R. F.; Hewage, N.; Brückner, C.; Gascón, J. A., *J. Phys. Chem. A* **2019,** *123*, 7470–7485.

[18] Frisch, M. J.; Trucks, G. W.; Schlegel, H. B.; Scuseria, G. E.; Robb, M. A.; Cheeseman, J. R.; Scalmani, G.; Barone, V.; Petersson, G. A.; Nakatsuji, H.; Li, X.; Caricato, M.; Marenich, A. V.; Bloino, J.; Janesko, B. G.; Gomperts, R.; Mennucci, B.; Hratchian, H. P.; Ortiz, J. V.; Izmaylov, A. F.; Sonnenberg, J. L.; Williams; Ding, F.; Lipparini, F.; Egidi, F.; Goings, J.; Peng, B.; Petrone, A.; Henderson, T.; Ranasinghe, D.; Zakrzewski, V. G.; Gao, J.; Rega, N.; Zheng, G.; Liang, W.; Hada, M.; Ehara, M.; Toyota, K.; Fukuda, R.; Hasegawa, J.; Ishida, M.; Nakajima, T.; Honda, Y.; Kitao, O.; Nakai, H.; Vreven, T.; Throssell, K.; Montgomery Jr., J. A.; Peralta, J. E.; Ogliaro, F.; Bearpark, M. J.; Heyd, J. J.; Brothers, E. N.; Kudin, K. N.; Staroverov, V. N.; Keith, T. A.; Kobayashi, R.; Normand, J.; Raghavachari, K.; Rendell, A. P.; Burant, J. C.; Iyengar, S. S.; Tomasi, J.; Cossi, M.; Millam, J. M.; Klene, M.; Adamo, C.; Cammi, R.; Ochterski, J. W.; Martin, R. L.; Morokuma, K.; Farkas, O.; Foresman, J. B.; Fox, D. J. *Gaussian 09 Rev. D.01*, Wallingford, CT, 2013.

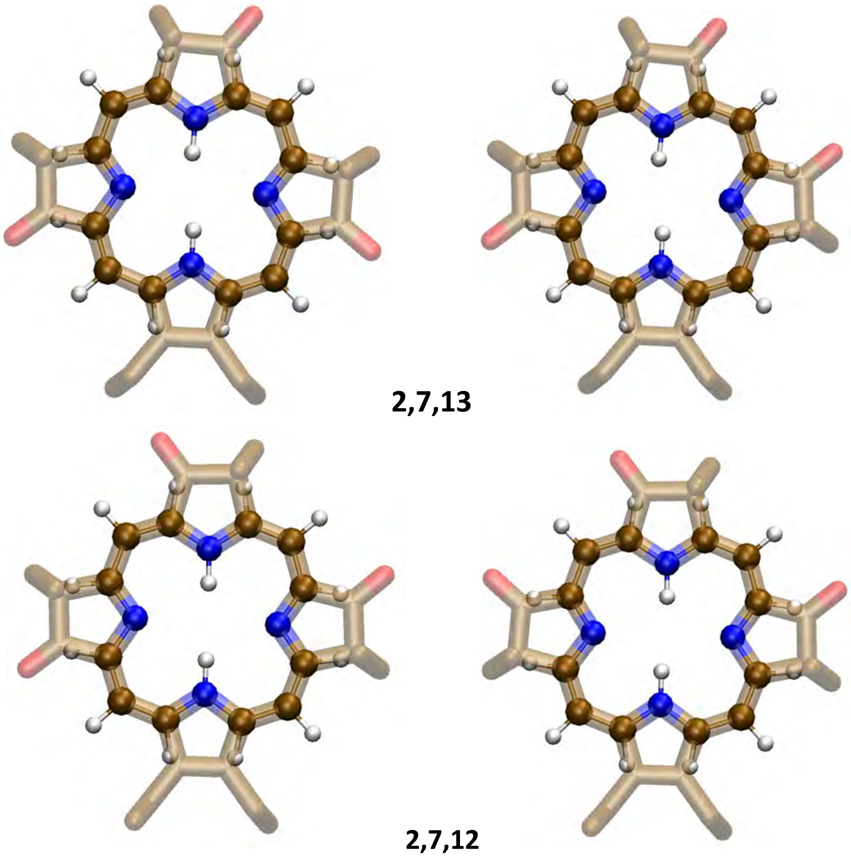

Figure S51. Overlay of the full computed structures of the triketones indicated and the 16-membered inner-inner-inner-inner resonance structure compound in which all atoms outside this macrocycle were replaced with hydrogen atoms.

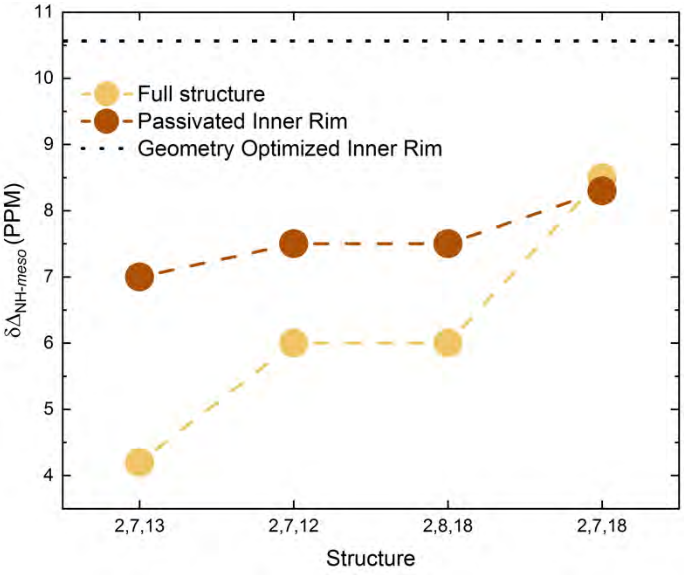

Figure S52. Computed differences in the average ^{1}H chemical shifts for the inner N-H and the outer *meso*-C-H protons ($\delta\Delta_{NH,meso}$) of the computed theoretical structures indicated above.
